# Multiomics and deep learning dissect regulatory syntax in human development

**DOI:** 10.1038/s41586-026-10326-9

**Published:** 2026-04-08

**Authors:** Betty B. Liu, Selin Jessa, Samuel H. Kim, Yan Ting Ng, Soon Il Higashino, Georgi K. Marinov, Derek C. Chen, Benjamin E. Parks, Li Li, Tri C. Nguyen, Austin T. Wang, Sean K. Wang, Meng How Tan, Serena Y. Tan, Michael Kosicki, Len A. Pennacchio, Eyal Ben-David, Anca M. Pasca, Anshul Kundaje, Kyle K. H. Farh, William J. Greenleaf

**Affiliations:** 1 Department of Bioengineering, Stanford University, Stanford, CA USA; 2 Department of Genetics, Stanford University, Stanford, CA USA; 3 Cancer Biology Program, Stanford University, Stanford, CA USA; 4 Medical Scientist Training Program, Stanford University, Stanford, CA USA; 5 Illumina Artificial Intelligence Laboratory, Illumina, Foster City, CA USA; 6 School of Chemistry, Chemical Engineering and Biotechnology, Nanyang Technological University, Singapore, Singapore; 7 Department of Computer Science, Stanford University, Stanford, CA USA; 8 Department of Pediatrics, Stanford University, Stanford, CA USA; 9 Basic Sciences and Engineering Initiative, Betty Irene Moore Children’s Heart Center, Lucile Packard Children’s Hospital, Stanford, CA USA; 10 Department of Ophthalmology, Stanford University, Stanford, CA USA; 11 Department of Pathology, Stanford University, Stanford, CA USA; 12 Environmental Genomics and System Biology Division, Lawrence Berkeley National Laboratory, Berkeley, CA USA; 13 US Department of Energy Joint Genome Institute, Berkeley, CA USA; 14 Comparative Biochemistry Program, University of California, Berkeley, CA USA; 15 Department of Applied Physics, Stanford University, Stanford, CA USA

**Keywords:** Development, Epigenomics

## Abstract

Transcription factors establish cell identity during development by binding regulatory DNA in a sequence-specific manner, often promoting local chromatin accessibility and regulating gene expression^1^. Mapping accessible chromatin offers critical insights into transcriptional control, but available datasets for human development are restricted to bulk tissue, single organs or single modalities^2^. Here we present the Human Development Multiomic Atlas, a single-cell atlas of chromatin accessibility and gene expression from 817,740 fetal cells across 12 organs, spanning 203 cell types and more than 1 million candidate *cis*-regulatory elements, many of which exhibit organ-specific in vivo enhancer activity. Deep learning models trained to predict accessibility from local DNA sequence unravel a comprehensive lexicon of motifs that influence accessibility, including composite motifs exhibiting distinct syntactic constraints that are predicted to mediate transcription factor cooperativity. We identify ‘hard’ syntactic rules requiring precise motif spacing and orientation, ‘soft’ rules allowing flexible motif arrangements, and ubiquitous motifs inhibiting accessibility. Model-based interpretation of genetic variants reveals that disruption of motifs with positive and negative effects is associated with concordant effects on gene expression. Our work delineates how motif syntax governs cell-type-specific chromatin accessibility and provides a foundational resource for decoding *cis*-regulatory logic and interpreting genetic variation during human development.

## Abstract (web summary)

The Human Development Multiomic Atlas catalogues single-cell accessibility and gene expression data from human fetal cells across 12 organs, enabling the inference of syntactic rules for motifs that govern cell-type-specific transcription factor binding and chromatin accessibility during human development.

## Main

During human development, the diversity of cell types arises through differential expression and activity of transcription factors, which integrate cell-intrinsic and -extrinsic signals to direct gene regulation^1^. Transcription factors bind specific sequences of DNA in *cis-*regulatory elements, often inducing local chromatin accessibility and altering the expression of proximal genes^2^. However, we lack a comprehensive view of the transcription factor motifs that drive chromatin state changes during human development, limiting our understanding of how transcription factor binding site organization—or syntax—contributes to regulation^3^.

Mapping chromatin accessibility using DNase I hypersensitive sites sequencing (DNase-seq) and assay for transposase-accessible chromatin using sequencing (ATAC–seq)^2,4^ has enabled inference of transcription factor activity via sequence motifs in human tissues^5,6^. However, bulk measurements obscure cellular heterogeneity, and most single-cell atlases have focused on individual organs^7,8^ or single omic modalities^9,10^. A multi-organ, multi-modal view is needed to capture the cell context specificity of *cis*-regulation and link chromatin state to transcriptional programs.

Chromatin accessibility often arises from cooperative transcription factor binding, either through direct interactions between transcription factors^11,12^ or through competition with nucleosomes^13–15^. These mechanisms respectively impose either hard (fixed) or soft (flexible) constraints on motif syntax. Yet the generality of such rules across human development is largely unknown. Furthermore, complex disease-associated genetic variants are enriched in the non-coding genome^16^, but our ability to predict the variants that disrupt regulatory activity in specific cell types remains limited.

Recent deep learning models trained to predict base-resolution chromatin accessibility profiles from local DNA sequence learn causal sequence features that influence accessibility^17–19^. Beyond de novo discovery of predictive motifs and transcription factor footprints, these models enable in silico interrogation of regulatory sequence syntax and non-coding genetic variants by predicting the quantitative effects of DNA sequence changes on accessibility^17,20^. Thus, deep learning models provide a powerful framework for decoding the logic of how transcription factor binding influences chromatin accessibility and linking sequence variation to disruption of *cis*-regulation.

Here, we present the Human Development Multiomic Atlas (HDMA), a multiomic, multi-organ single-cell atlas that profiles chromatin accessibility and gene expression in 12 human fetal organs. We mapped more than one million accessible regulatory elements, and demonstrated their ability to resolve organ-specific and cell-type-specific enhancer activity in vivo. We trained and interpreted deep learning models to predict cell-type-resolved accessibility, defined a lexicon of regulatory sequence motifs driving accessibility, and inferred predictive motif instances across the genome per cell type. Interrogation of motif syntax uncovered both hard and soft syntactic constraints. Finally, we prioritized disease-associated variants that are likely to perturb regulatory function during development. HDMA provides a foundational resource for decoding *cis*-regulatory syntax, linking sequence variation to gene regulation, and understanding how DNA sequence influences transcriptional regulation during human development.

## A multiomic human development atlas

**Fig. 1:**
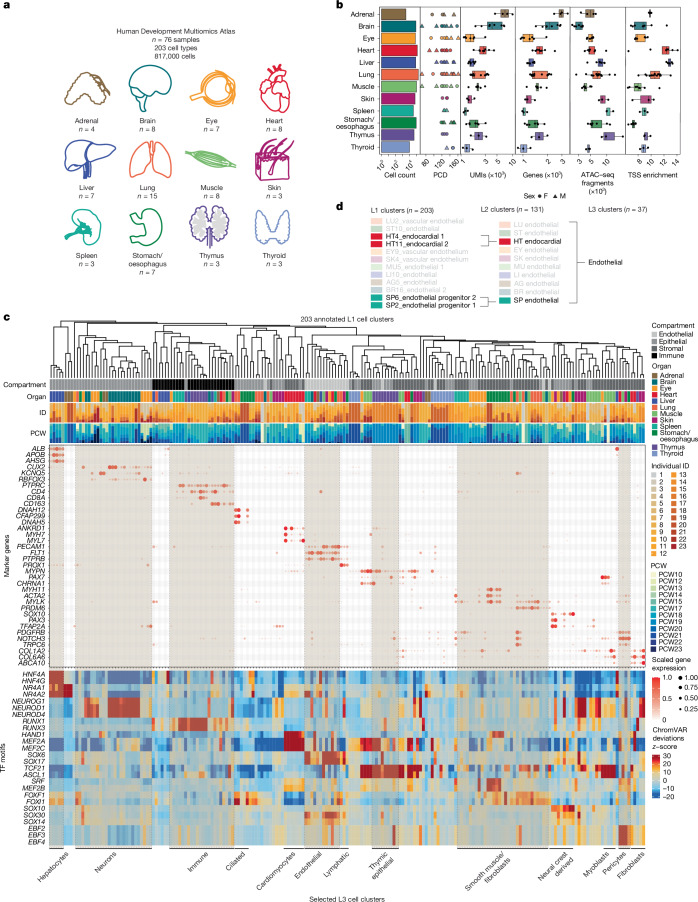
A multiomic, multi-organ atlas of human development. **a**, HDMA dataset overview and sample size distribution by organ. **b**, Sample metadata and quality control metrics for RNA-sequencing and ATAC–seq modalities, with samples ranging from post-conception weeks (PCW) 10–23. The number of biologically independent samples per organ is shown in **a**. PCD, post-conception days. **c**, Key marker gene expression and transcription factor motif chromVAR deviation *z*-score for 203 hierarchically clustered cell types. TF, transcription factor. **d**, An example of different levels of cell annotation for endothelial cells. AG, adrenal gland; BR, brain; EY, eye; HT, heart; LI, liver; LU, lung; MU, muscle; SK, skin; SP, spleen; ST, stomach/oesophagus.

**Extended Data Fig. 1:**
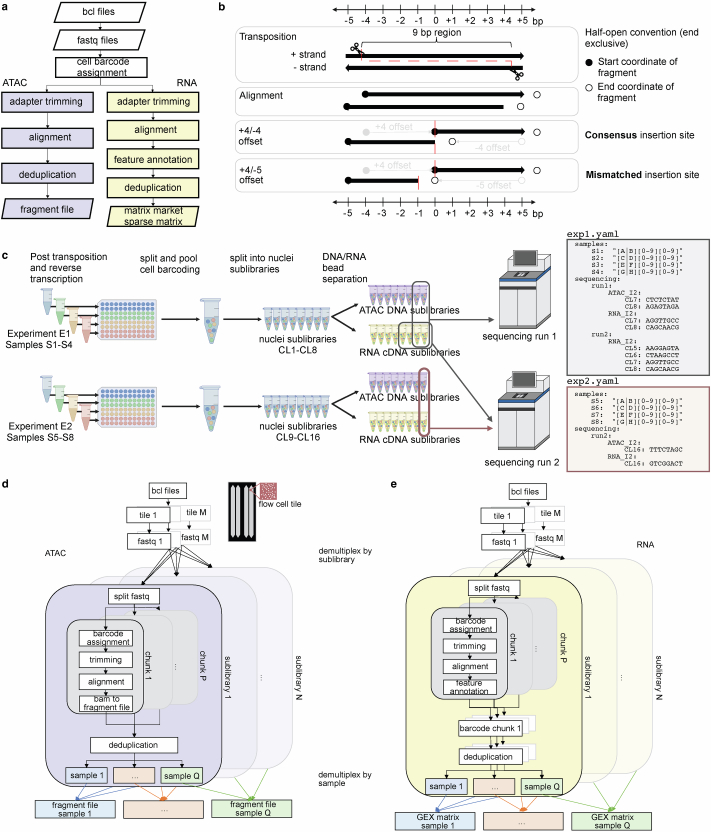
A highly parallelized, rapid and storage-efficient pre-processing snakemake pipeline to convert BCL files to ATAC fragment files and RNA sparse matrices. **a**) Overview of pre-processing workflow. Files are presented in parallelograms while processes are in rectangles. **b**) Schematic explaining the +4/−4 offset adopted by this pipeline to reach a consensus Tn5 insertion site. **c**) Definition of a SHARE-seq experiment, samples, nuclei sublibraries, ATAC and RNA sublibraries. Example snakemake configuration file snippets to process sequencing data from mixed experiments. Each experiment should be run in a separate snakemake directory. Detailed pre-processing workflow showing the parallelization behind the scenes for **d**) ATAC and **e**) RNA sequencing data.

**Extended Data Fig. 2:**
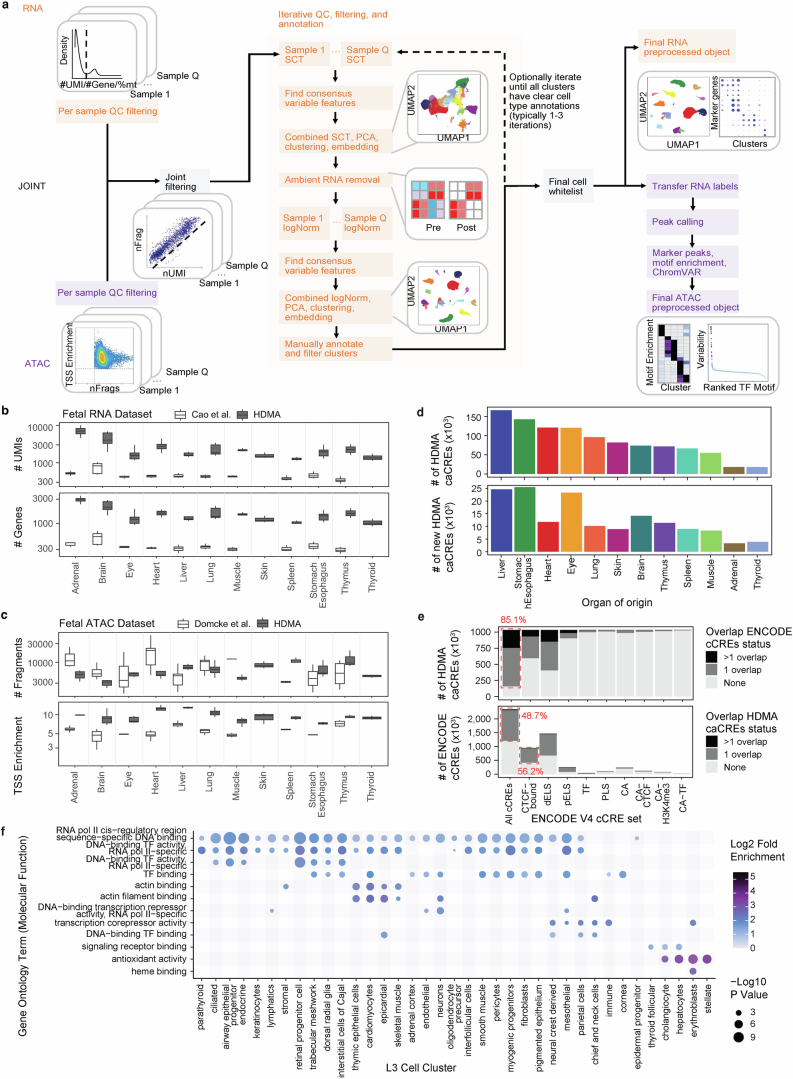
Cell type annotation workflow and regulatory elements landscape overview. **a**) Overview of the iterative cell type annotation pipeline converting fragment files to annotated ArchR project for ATAC data and sparse matrices to annotated Seurat project for RNA data. **b**) Comparison of the number of UMIs and number of genes detected per cell in RNA modality with Cao et al. and **c**) comparison of the number of fragments and TSS enrichment ratio per cell in ATAC modality with Domcke et al. The number of biologically independent samples per organ is shown in Fig. 1a. **d**) Organ origin of all HDMA caCREs and de novo HDMA caCREs. **e**) Overlap between HDMA caCREs and all or subsets of ENCODE v4 cCREs. dELS, distal enhancer-like signature. pELS, proximal enhancer-like signature. PLS, promoter-like signature. CA, chromatin accessible. **f**) Top Gene Ontology molecular function terms enriched in the highly linked genes (HLG) in each broad class cell cluster (L3 cell cluster), with statistical significance determined by a one-sided Fisher’s Exact Test.

We simultaneously profiled chromatin accessibility and gene expression from 817,740 fetal cells spanning 12 organs between post-conception weeks 10 and 23 using SHARE-seq^21^, a scalable split-and-pool combinatorial barcoding platform (Fig. 1a, Extended Data Figs. 1 and  2a and Supplementary Note 1). The resulting HDMA captures true multiomic measurements from the same cells, with improved transcription start site (TSS) enrichment, number of unique molecular identifiers (UMIs) and number of genes detected compared with previous multi-organ fetal ‘single-ome’ atlases^9,10^ (Fig. 1b, Extended Data Fig. 2b,c and Supplementary Table 1).

We annotated 203 distinct cell clusters (L1 clusters) using an iterative approach combining canonical compartment markers, known cell type markers and de novo marker genes from our single-cell transcriptomes (Methods). Cell identity was corroborated by significant transcription factor motif enrichments within cluster-specific accessible chromatin peaks (Supplementary Note 2).

Hierarchical clustering of pseudobulk expression profiles for the top marker genes revealed that cell types common to different organs—such as endothelial cells, fibroblasts and immune cells—often clustered together (Fig. 1c). Based on shared marker gene expression, we manually grouped related L1 clusters into 134 collapsed cell clusters (L2 clusters) and further into 37 broad cell classes (L3 clusters) (Fig. 1d and Supplementary Tables 2 and  3).

Global chromVAR^22^ deviation analysis identified transcription factor motifs with increased accessibility that were shared across L1 clusters of the same cell type (Fig. 1c). Compared with marker genes, transcription factor motif accessibility variations were generally less specific, with many motifs exhibiting pleiotropic accessibility patterns across cell types. For example, whereas expression of the *CUX2* marker gene was restricted to neurons, the top motif in neurons, *NEUROD1*, showed increased accessibility in neurons, fibroblasts and neural crest-derived cell types, reflecting motif degeneracy and context-dependent activity of transcription factors during development.

## Accessibility landscape of development

We catalogued the accessible regulatory landscape of human development by aggregating peaks independently called across the 203 L1 cell types. Iterative overlap of peaks defined a global set of 1,032,273 chromatin-accessible *cis*-regulatory elements (caCREs), each spanning 500 bp, collectively covering about 17% of the genome. Most caCREs had their highest normalized ATAC–seq signal in liver, eye, heart, and stomach and oesophagus (stomach/oesophagus) cell types (Extended Data Fig. 2d).

Comparison with the ENCODE v4 database^23^ revealed that 85.1% of HDMA caCREs overlapped previously characterized candidate CREs (cCREs) (Extended Data Fig. 2e). Notably, we recovered 48.7% of all ENCODE v4 sites, including 56.2% of CTCF-bound elements. Among the 14.9% of caCREs that did not overlap ENCODE cCREs, brain- and eye-derived elements were disproportionately represented, suggesting that single-cell profiling uncovers cell-type-specific regulatory elements that are absent from ENCODE bulk-derived datasets (Extended Data Fig. 2d).

**Fig. 2:**
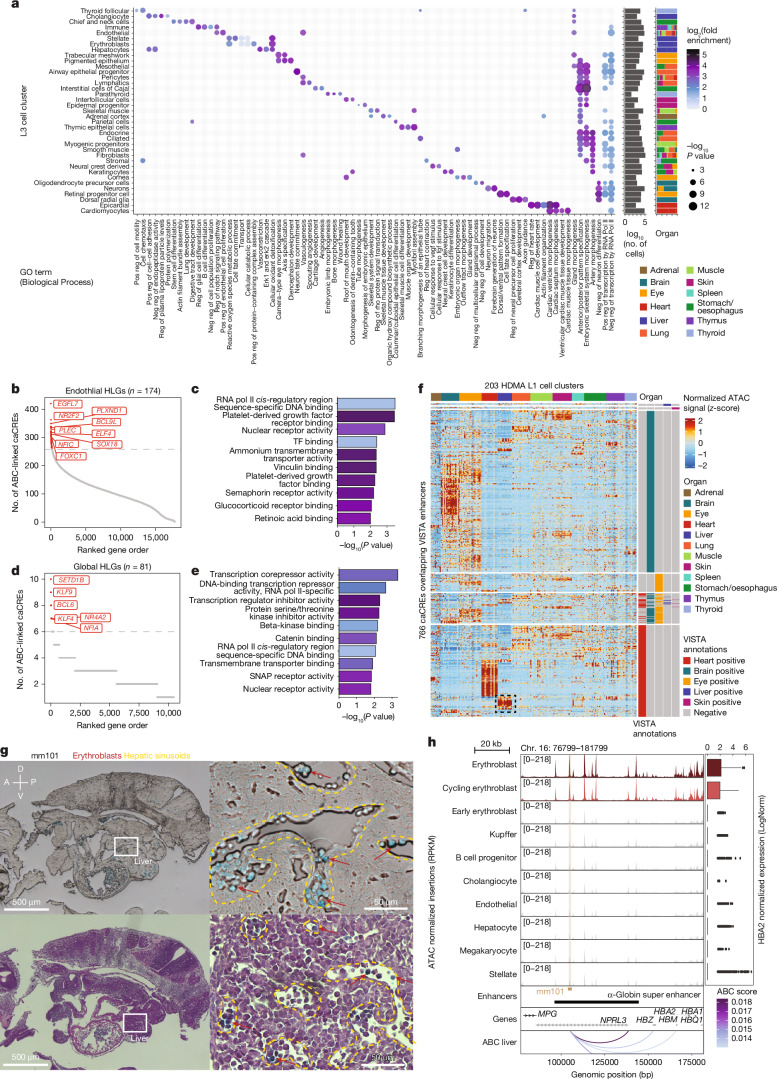
Connecting DNA regulatory elements to genes and resolving organ and cell-type specificity in experimentally validated enhancers. **a**, Top 5 GO Biological Process terms enriched in the HLG in each broad class cell cluster (L3 cell cluster). Pos, positive; neg, negative; reg, regulation. **b**,**d**, Endothelial (**b**) and global (**d**) HLGs with the most ABC-linked caCREs. **c**,**e**, GO Molecular Function term enrichment in endothelial HLGs (**c**) and global HLGs (**e**) with statistical significance determined by a one-sided Fisher’s exact test. **f**, Normalized and *z*-scored ATAC–seq signal in the HDMA caCREs overlapping VISTA enhancers. **g**, Bright-field (top) and H&E (bottom) staining images of VISTA embryo mm101 sections. Blue colour is from X-Gal staining, indicating where the enhancer is active. Sectioning and histology was performed on one whole mouse embryo carrying that enhancer. Imaging was repeated on two sections with similar results. A, anterior; D, dorsal; P, posterior; V, ventral. **h**, Accessibility at VISTA enhancer mm101 and ABC-linked α-globin gene (*HBA2*) expression in liver cell types. RPKM, reads per kilobase per million mapped reads.

We linked caCREs to genes using the activity-by-contact (ABC) model^24^ applied within each L1 cell cluster. For each broad L3 cluster, we aggregated ABC links from its constituent L1 clusters and filtered to obtain elements targeting genes with accessible promoters. In each L3 cluster, we defined highly linked genes (HLGs) as the top 1% of genes with the most ABC-linked caCREs (Supplementary Table 4). Gene Ontology (GO) analysis revealed that HLGs were enriched for cell-type-specific processes, such as ‘B cell proliferation’ in immune cells, ‘keratinocyte differentiation’ in keratinocytes and ‘camera-type eye morphogenesis’ in pigmented epithelial cells (Fig. 2a). Broad transcriptional regulation-related terms including ‘positive/negative regulation of transcription by RNA Pol II’ were consistently enriched across 25 out of 37 L3 clusters. Other broadly enriched developmental terms, such as ‘embryonic skeletal system morphogenesis’ and ‘anterior/posterior pattern specification’, were driven by HOX genes known to regulate developmental programs^25^. GO molecular function analysis confirmed that HLGs were enriched for transcription factor binding activity across 33 out of 37 L3 clusters (Extended Data Fig. 2f). For example, endothelial HLGs included canonical regulators (*NR2F2*, *ELF4*, *SOX18*, *NFIC* and *FOXC1*) and coregulators (*BCL9L*), along with genes related to endothelial functions (*EGFL7*, *PLXND1* and *PLEC*). The top GO terms enriched in endothelial HLGs included general transcription factor activity terms and endothelial-specific terms such as PDGFR binding (Fig. 2b,c).

To identify conserved regulation by caCREs across cell types, we filtered them to obtain caCRE–gene links consistently identified in all L3 clusters. These global HLGs (gHLGs), corresponding to the top 1% of genes with the most consistent ABC-linked caCREs, were also significantly enriched for transcription factor activity (Fig. 2d,e), and included ubiquitous regulators (*KLF9*, *KLF4* and *BCL6*). These 81 gHLGs constitute a set of ubiquitously highly regulated transcriptional regulators across human development, and indicate that transcription factors are among the most highly regulated genes in development, consistent with our previous observations in the developing human brain^7^ (Supplementary Table 4).

## Resolving regulatory element specificity

**Extended Data Fig. 3:**
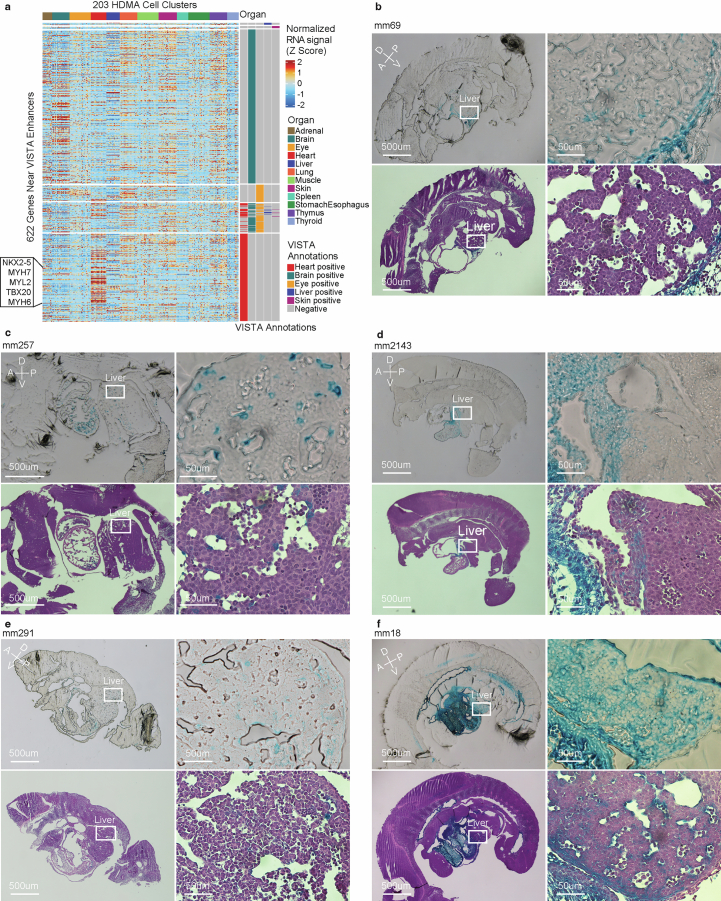
VISTA enhancer gene expression and organ specificity validation. **a**) Normalized and Z-scored expression of the nearest gene in HDMA for each VISTA enhancer, grouped by VISTA-annotated organ positivity. **b-f**) Bright-field (top) and H&E images (bottom) for 5 VISTA enhancers re-annotated as positive in the liver based on HDMA data. Blue colour is from X-Gal staining indicating where the enhancer is active. For each enhancer shown, sectioning and histology was performed on one whole mouse embryo carrying that enhancer. Imaging was repeated on at least 2 sections per embryo with similar results. A, anterior. P, posterior. D, dorsal. V, ventral.

To validate HDMA-derived enhancers, we examined caCREs that overlapped experimentally validated enhancers from the VISTA database, assayed by X-gal reporter staining at mouse embryonic day 11.5 (refs. ^26,27^). Among the 3,272 VISTA enhancers with hg38 coordinates, 851 overlap with organs profiled in HDMA, including 766 that intersect with the 1,032,273 caCREs. For VISTA enhancers annotated as active in brain, heart and eye, we observed strong enrichment of both accessibility (one-sided Wilcoxon rank-sum test, *P* = 10^−437^ for brain, *P* = 10^−452^ for heart and *P* = 9.11 × 10^−48^ for eye; AUROC probability = 0.72 for brain, 0.75 for heart and 0.59 for eye) (Fig. 2f) and gene expression (one-sided Wilcoxon rank-sum test, *P* = 1.04 × 10^−38^ for brain, *P* = 1.23 × 10^−166^ for heart and *P* = 2.77 × 10^−23^ for eye; AUROC probability = 0.57 for brain, 0.67 for heart and 0.57 for eye) (Extended Data Fig. 3a) in the HDMA cell type clusters from the corresponding organs.

Surprisingly, several enhancers previously annotated as heart-specific exhibited strong accessibility exclusively in liver cell types in our data (Fig. 2f, dashed black box). Given the natural colour and position of the mouse liver directly beneath the heart, these cases may have been previously missed by visual inspection alone. Expert review of our top candidate enhancers postulated as active in the liver confirmed six enhancers (mm2143, mm69, mm291, mm101, mm257 and mm18) as active in both liver and heart^26^. Sectioning and imaging of X-gal-stained embryos validated liver activity for all six candidates (Fig. 2g and Extended Data Fig. 3b–f).

One candidate, mm101, overlapped the known α-globin super-enhancer locus^28^ and was linked by ABC analysis to the α-globin gene cluster (Fig. 2h). α-Globin is a subunit of fetal haemoglobin present exclusively in erythroblasts^29^. As the fetal liver is a site of erythropoiesis during development, we hypothesized that mm101 enhancer activity would be restricted to erythroblasts. Indeed, accessibility at the mm101 locus and expression of HBA2 were both increased specifically in liver erythroblasts and cycling erythroblasts (Fig. 2h). Histological analysis confirmed X-gal positivity in liver erythroblasts characterized by a globular morphology and characteristic darkened purple colour from haematoxylin and eosin (H&E) staining, which reside within hepatic sinusoids (Fig. 2g).

## The transcription factor motif lexicon

**Fig. 3:**
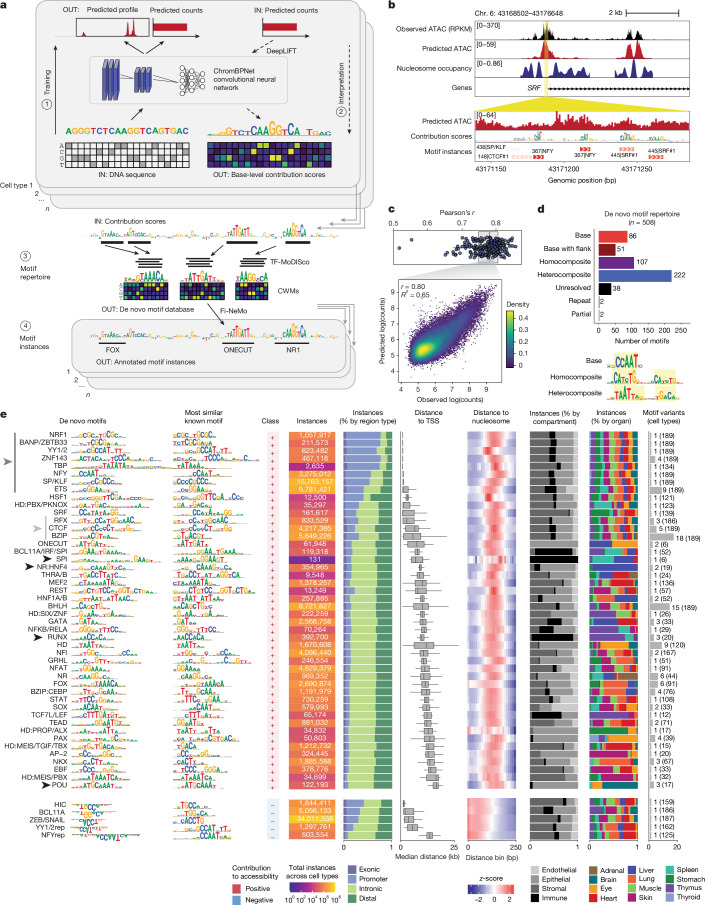
Repertoire of transcription factor motifs in human development. **a**, Overview of workflow. For each cell type, a ChromBPNet model is trained to predict accessibility from local DNA sequence. Models are interpreted to derive per-base scores representing contribution to accessibility. High-contribution regions are clustered within and across cell types into motifs, and motifs are used to annotate high-contribution regions in each cell type. CWM, contribution weight matrix. **b**, Example of the *SRF* locus in cardiomyocytes, showing observed and predicted chromatin accessibility, inferred nucleosome occupancy, per-base contribution scores and annotated motif instances. **c**, Top, distribution of Pearson correlation between predicted and observed log counts in peak regions per model (mean across five folds). Bottom, predicted versus observed log counts in peak regions for one cell type, one model fold. **d**, Summary and categorization of unique de novo motifs, and example motifs from select categories. **e**, Summary of base motifs, one row per broad group of base motifs. Left to right: de novo motif representation as a CWM, most similar known motif (position weight matrix (PWM)), direction of contribution to accessibility, total number of genomic instances across cell types, proportion of instances overlapping various genomic features, distribution of median distance of instances (per cell type) to nearest TSSs, proportion of instances from cell types in each tissue compartment, proportion of instances from cell types in each organ, and number of motif variants within each broad motif group. For each broad motif, the number of cell types for which hits were identified is indicated in parentheses, and corresponds to the number of cell types included in each box plot distribution. Arrowheads indicate motifs that are referred to in the text: ubiquitous promoter-dominant motifs (dark grey), ubiquitous distal motifs (light grey) and tissue-specific distal motifs (black).

**Extended Data Fig. 4:**
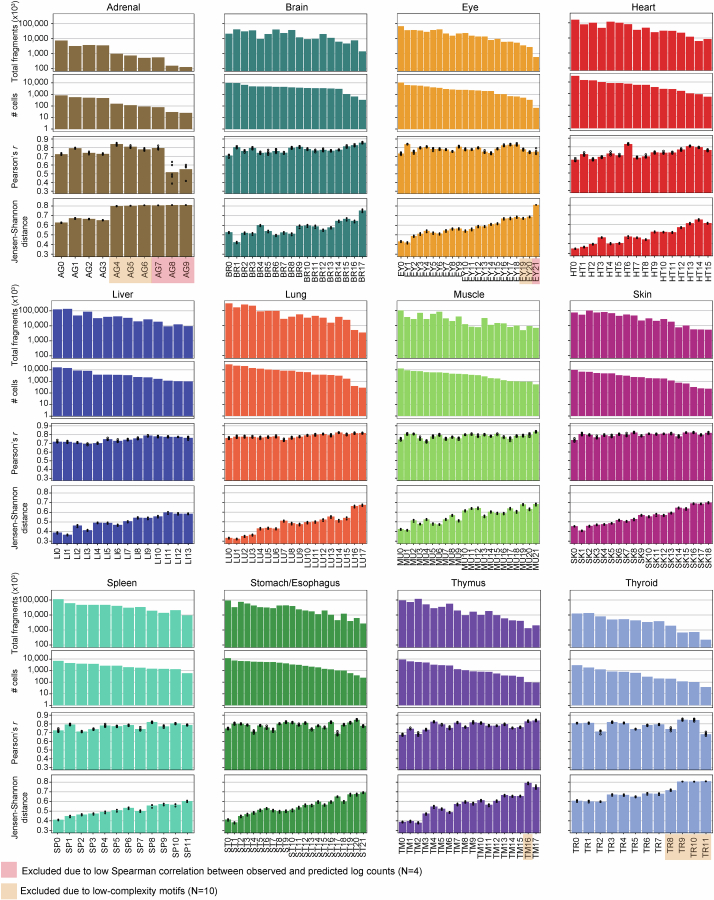
Quality control and performance of ChromBPNet deep learning models per cell type. For each cell type, the total number of fragments and cells is shown, along with the Pearson’s correlation coefficient between predicted and observed log counts and the Jensen-Shannon Distance between predicted and observed accessibility profiles. For Pearson’s correlation and Jensen-Shannon distance, bars represent the mean across five model folds, and points represent the performance per fold. Models excluded from downstream analysis due to low correlation between observed and predicted counts (n = 4) are indicated in red, and models excluded due to learning predominantly low-complexity sequence motifs (n = 10) are indicated in orange.

**Extended Data Fig. 5:**
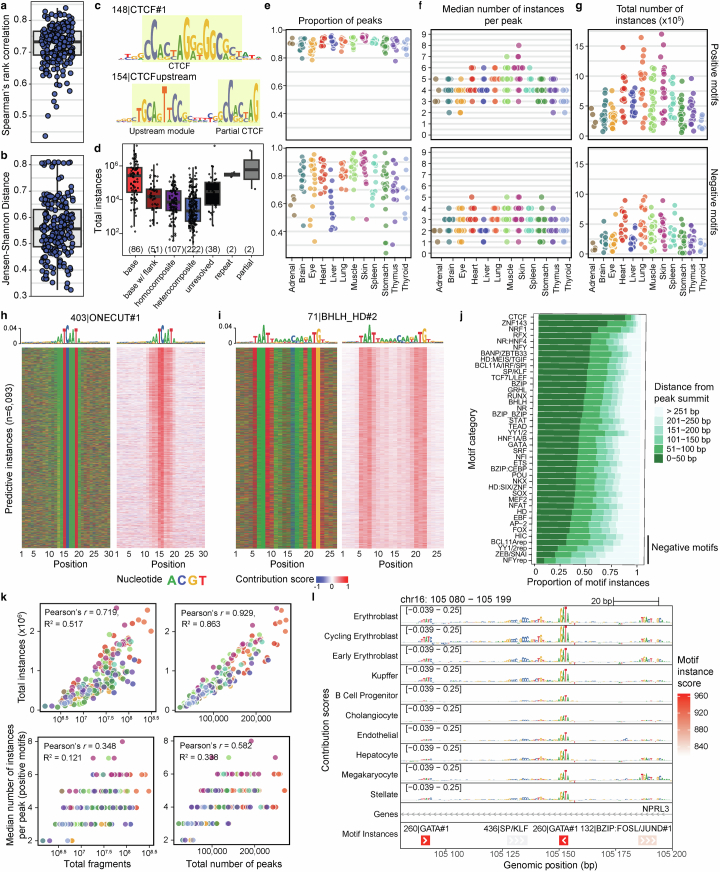
Modeling of sequence determinants of chromatin accessibility. **a**) Distribution of Spearman correlation between predicted and observed log counts in peak regions per model (mean across five folds) shown for n = 199 cell types. **b**) Distribution of Jensen-Shannon Distance between predicted and observed accessibility profiles in peak regions per model (mean across five folds) shown for n = 199 cell types. **c**) Example contribution weight matrix (CWM, neural network-derived motif representation) for distinct CTCF upstream and CTCF motifs. **d**) Total number of motif instances across all cell types for the 508 lexicon motifs, grouped by motif category. Each point is one motif, with the number of motifs per category indicated in parentheses. **e**) Proportion of peaks with at least one positive or one negative motif instance called, per cell type. **f**) Median number of motif instances per peak per cell type, for all peaks with instances called. **g**) Total number of motif instances per cell type. **h**) Illustrative representations of all motif instances for ONECUT motif in liver hepatocytes. Heatmaps indicating nucleotide composition (left) and contribution scores (right) for 30 bp centered at all 6,093 motif instances. **i**) Same as (h), for a BHLH, HD composite motif. **j**) Proportion of total motif instances across cell types at each distance from peak summits, for each motif category, sorted by proportion of instances within 50 bp of peak summits. **k**) Relationship between total number of motif instances and median positive motif instances, and total number of fragments or peaks for a given cluster. **l**) Contribution scores of GATA motif instances within mm101 enhancer in liver cell types.

To identify *cis*-regulatory sequence features predictive of chromatin accessibility, we trained deep convolutional neural networks (ChromBPNet) to model the shape and magnitude of ATAC–seq profiles from the local DNA sequence^17,18^. For each cell type, we trained models to predict total read counts and base-resolution distribution of reads in 1,000-bp windows in peaks and background regions using 2,114 bp of local sequence context as input (Fig. 3a and Methods). We trained fivefold cross-validated models for 203 cell types and retained models for 189 cell types (*n* = 945 total models) that achieved a median Pearson correlation of 0.78 between predicted and observed log-read counts in held-out peaks in test chromosomes (Fig. 3b,c, Extended Data Figs. 4 and  5a,b and Supplementary Table 5).

We investigated which *cis*-regulatory sequence features influence accessibility. We expect these sequences to correspond to sites bound by transcription factors, which protect nucleotides they occlude from Tn5 cleavage, but promote local accessibility in their vicinity^2^. We estimated the predictive importance of each nucleotide for accessibility separately in each cell type using DeepLIFT^30^, aggregated features into motifs using TF-MoDISco^31^, and clustered motifs across cell types to yield a lexicon of 508 de novo motifs that are predicted to regulate chromatin accessibility across 189 cell types^30^ (Fig. 3a,d,e and Methods).

We annotated de novo motifs according to the transcription factors that are likely to bind them, typically at the transcription factor family level, given the motif similarity within families (Methods and Supplementary Table 6). Several motifs matched known transcription factor binding motifs in existing databases (base motifs) or known motifs with additional predictive flanking nucleotides (base with flanks) (Fig. 3d). The lexicon also recovered variants of canonical motifs, including both the core and upstream CTCF motifs, reflecting DNA contacts by distinct CTCF zinc-fingers^5,32^ (Extended Data Fig. 5c). More than half of the de novo motifs were composite motifs, comprising either two homotypic (homocomposites) or heterotypic sites (heterocomposites). A subset of 38 ‘unresolved’ motifs lacked similarity to known databases. Most motifs (*n* = 493, 97%) had positive contribution scores, indicating that they promote chromatin accessibility, whereas a subset had negative scores, suggesting that they reduce accessibility (Fig. 3e and Supplementary Table 6). We refer to these as ‘positive’ and ‘negative’ motifs, respectively, on the basis of their directional influence on accessibility.

Next, we used Fi-NeMo to identify predictive instances of each motif in accessible peaks of each cell type (Fig. 3a, Methods). On average, each cell type harboured 839,544 motif instances, with 2–8 instances per peak and 65% of instances within 150 bp of peak summits (Extended Data Fig. 5d–j). The number of motif instances scaled with the total number of peaks in each cell type, suggesting that deeper sequencing could recover additional motif instances (Extended Data Fig. 5k).

As an example, we revisited the mm101 enhancer active in fetal liver erythroblasts. Model interpretation revealed two high-scoring GATA motif instances within the enhancer (Extended Data Fig. 5l), consistent with the essential role of GATA1 in erythropoiesis and supporting its contribution to α-globin gene activation in this context^33^.

**Extended Data Fig. 6:**
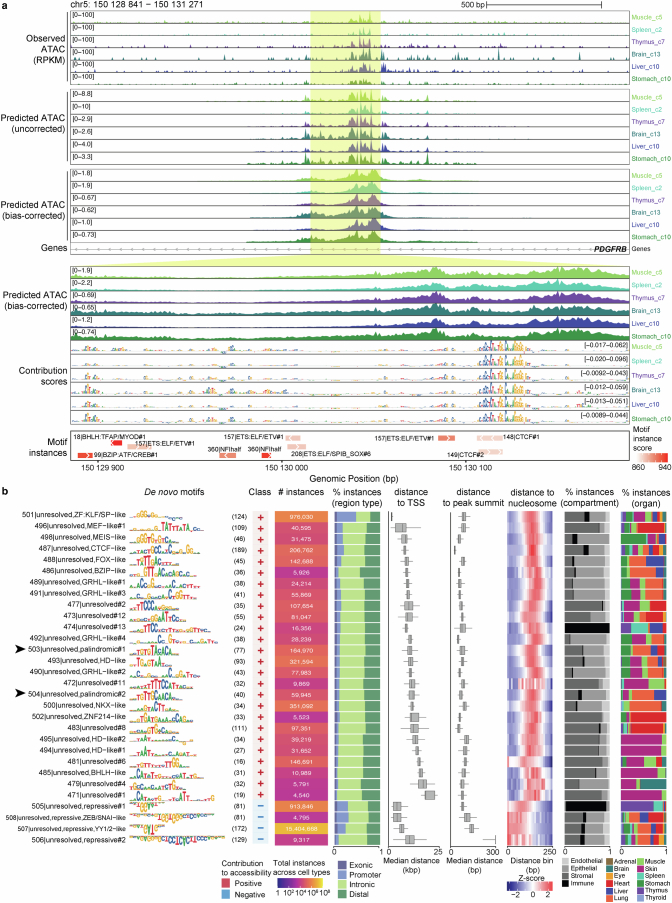
Example sequence contributions and sequence motifs learned from ChromBPNet models. **a**) Visualization of *PDGFRB* locus with observed, predicted uncorrected accessibility, and predicted bias-corrected accessibility for several endothelial cell types from different tissues. Zoomed region shows predicted bias-corrected accessibility and basepair-resolution contribution scores. **b**) Summary of unresolved de novo motifs not matching known motifs. From left: CWM representation, number of cell types for which hits were identified, motif class (based on contribution to accessibility), total number of instances across cell types, and distribution of motif instances by organ. Two palindromic unresolved motifs are indicated with arrowheads. The boxplot distributions are calculated over cell types in which motif hits were identified, indicated in parentheses.

We next stratified predictive motif instances by genomic context and cell type specificity, and computed distances to the nearest TSS and nucleosome dyad inferred using NucleoATAC^34^ (Fig. 3e). This analysis revealed a set of ubiquitous, promoter-dominant, TSS-proximal motifs, including NRF1, NFY, YY1 or YY2 (YY1/2), TBP, SP or KLF (SP/KLF), ETS and BANP. These motifs are generally CG-rich, consistent with the CpG-rich nature of promoters. NRF1 and BANP are known to be DNA methylation-sensitive transcription factors^35,36^, and several others have been implicated in transcription initiation^37–39^. By contrast, organ and cell-type-specific motifs were predominantly located in distal or intronic regions. These included SPI and RUNX (immune cells), POU (eye and brain) and HNF4 (liver) (Fig. 3e). In cell types such as endothelial cells, which arise in several organs, independently trained models learned consistent sequence contributions (Extended Data Fig. 6a), suggesting robust discovery of regulatory lexicons. We also identified two unresolved palindromic elements with restricted usage in stomach, lung and liver (Extended Data Fig. 6b).

**Extended Data Fig. 7:**
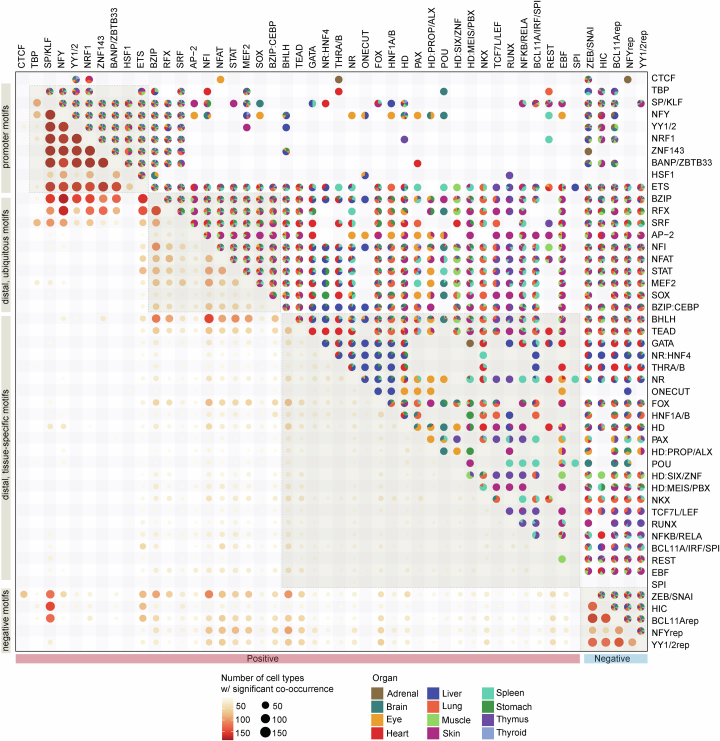
Motif co-occurrence analysis. Motif co-occurrence analysis between all base motifs at the broad annotation level. For each motif pair, the lower triangle represents the number of cell types where a significantly enriched co-occurence between motifs was detected, and the upper triangle represents the proportion of cell types from each organ where a significantly enriched co-occurrence between motifs was detected. Significantly co-enriched motifs were detected using one-sided Fisher’s Exact Tests.

To understand context-dependent combinatorial motif logic, we tested all base motif pairs in each cell type for significant co-occurrence of their motif instances (Extended Data Fig. 7). CTCF exhibited limited co-occurrence with other motifs, whereas promoter-dominant motifs were mutually co-enriched ubiquitously across tissues. We uncovered several tissue-dependent motif partnerships, such as ONECUT motifs co-enriched with HD and PAX motifs in eye cell types, and FOX and HNF1A or HNF1B motifs in the liver.

## Inferring distinct modes of transcription factor synergy

Combinatorial transcription factor binding at regulatory elements can enhance specificity and function, often through cooperative interactions. In DNA-mediated cooperativity, transcription factors with direct protein–protein interactions bind a specific composite recognition site at fixed arrangements, or, alternatively, DNA at such sites stabilizes weak interactions between transcription factors^11,12^. By contrast, nucleosome-mediated cooperativity arises from active or passive competition between transcription factors and nucleosomes^13,14^. We reasoned that DNA-mediated cooperativity imposes fixed spacing and orientation constraints on binding sites (hard syntax) within protein–protein interaction length-scales (<20 bp), whereas nucleosome-mediated cooperativity is compatible with flexible binding site organization (soft syntax) and longer distances between sites^40^ (20–150 bp).

**Fig. 4:**
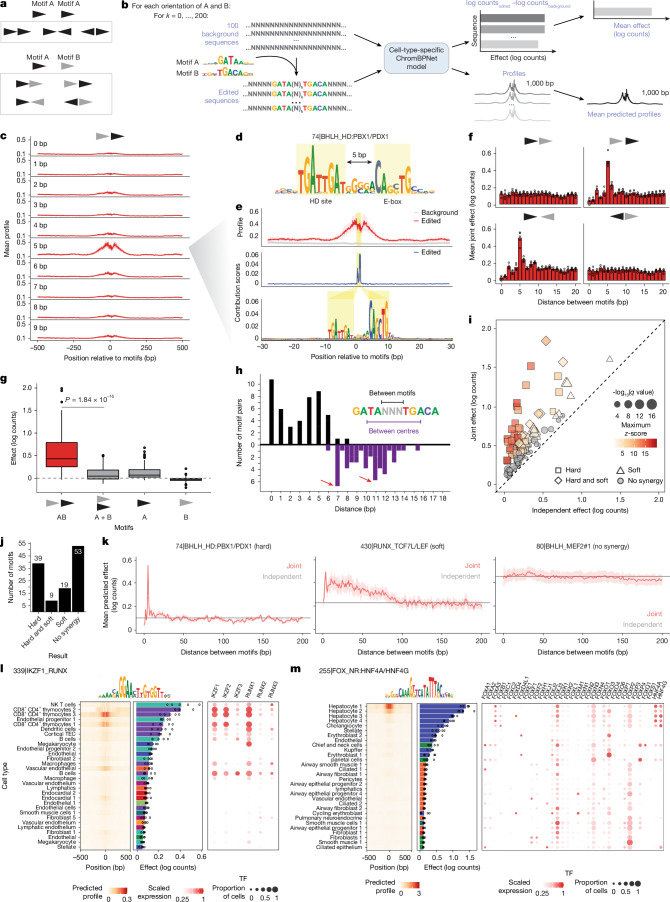
Systematic inference of transcription factor cooperativity. **a**, Unique orientations of one or two distinct motifs. **b**, Workflow for in silico marginalization. **c**–**g**, Synergy analysis for a composite motif with an HD site and BHLH site (E-box). **c**, Predicted profile when motifs are inserted at 0–9 bp distance, for one arrangement. Profiles are shown as mean ± s.d. across five folds. **d**, CWM for the composite motif. **e**, Predicted profile at 5 bp, and mean contribution scores after model interpretation of edited sequences with motifs inserted. Highlights indicated inserted nucleotides. **f**, Quantified predicted joint effects on accessibility of two motifs in log counts, per spacing and orientation. Each point represents one fold, and bar represents the mean. **g**, Predicted effects across *n* = 100 sequences. *P* value by one-sided Wilcoxon signed-rank test. **h**, Distance between motifs (top) and between motif centres (bottom) for all composite motifs inferred to have hard syntax preferences. **i**, Predicted joint effects (at optimal motif arrangement) and sum of independent effects for all motifs pairs tested. **j**, Number of composite motifs in each result syntax category. **k**, Predicted joint effects, compared with sum of independent effects, for three representative composite motifs. Effect curves are shown as mean ± s.d. across five folds. **l**, Predicted mean accessibility profile (left) and mean effect (middle) for in silico marginalization of the IKZF–RUNX composite motif in each cell type, and expression of transcription factors that could bind constituent motifs in each cell type (right). Error bars indicate s.d. across model folds. The 40 cell types with the highest predicted accessibility are shown. **m**, Same as **l**, for the FOX–HNF4 composite motif. NK, natural killer; TEC, thymic epithelial cell.

To systematically identify synergistic transcription factor interactions and syntactic constraints for each composite motif identified de novo, we implemented an in silico marginalization framework using our ChromBPNet models and the tangermeme package^41^, which exhaustively evaluates the joint effects of the two constituent motifs on chromatin accessibility across various spacing or orientation arrangements, compared with their independent effects^17,19,42^ (Fig. 4a,b and Methods). We repeated this analysis in every cell type, but focused our analysis on the cell type with the most predictive instances of the composite motif (Methods, Supplementary Note 3 and Supplementary Table 7). We defined synergy as a significant deviation from a log-additive model of independent motif effects, and further classified synergistic composite motifs as exhibiting hard or soft syntax on the basis of the motif distances and effect magnitude where joint effects exceeded additive expectations (Methods).

**Extended Data Fig. 8:**
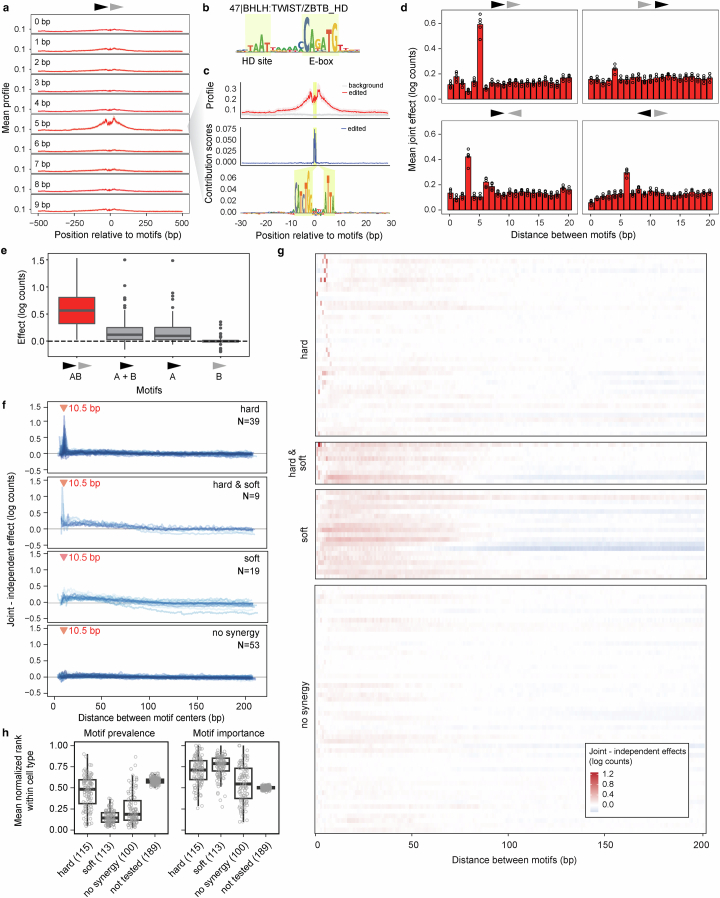
Motif synergy and syntax preferences. **a-e**) Synergy analysis for Coordinator-like composite motif. **a**) Predicted profile for Coordinator constituent motifs (BHLH, HD) inserted at 0-9 bp distance between motifs, for one arrangement. **b**) CWM for composite motif. **c**) Predicted profile at 5 bp distance between Coordinator motif constituents, and mean contribution scores after model interpretation of edited sequences with motifs inserted. **d**) Quantified effects in log counts, per spacing and orientation. Each point represents one fold, and the bar represents the mean across folds. **e**) Distribution of predicted effects across n = 100 sequences, for both motifs in their optimal arrangement, the sum of motifs A and B inserted independently, and motifs A and B alone. **f**) Difference between predicted joint effects for all composite motifs tested, and sum of independent effects. The best orientation for each motif is shown. **g**) Heatmap representation of joint minus independent effects as in (f). **h**) Distribution of mean normalized rank across motifs in each synergy group within each cell type, for motifs ranked based on prevalence defined by number of predictive instances (left) or importance defined by summed contribution scores across the motif CWM (right) (see Methods). Two-sided Wilcoxon rank-sum test p values for prevalence: hard vs soft (1.07 × 10^−26^), hard vs no synergy (7.15 × 10^−13^), hard vs not tested (1.33 × 10^−20^); and for importance: hard vs no synergy (5.34 × 10^−7^), hard vs not tested (1.33 × 10^−20^), soft vs no synergy (8.00 × 10^−13^), soft vs not tested (2.83 × 10^−20^). Only positive motifs were considered.

We recovered several known syntax-constrained composite elements. A de novo motif matching the recently described Coordinator element^43,44^—a composite of E-box and TAAT homeodomain motifs—showed strong synergy at a 5 bp head-to-tail arrangement (Extended Data Fig. 8a–e). Remarkably, this arrangement precisely matched the experimentally validated binding geometry of the Coordinator complex, in which TWIST1–TCF4 heterodimers and ALX4 bind cooperatively. X-ray crystallography showed that this strict motif spacing is necessary for stabilizing contacts between the TWIST1 backbone and an ALX4 side chain^43^. We predominantly detected this motif in skin cell types, consistent with its role in neural crest-derived mesenchyme. We observed a similar syntax-constrained synergistic effect for a distinct composite motif composed of an E-box and an alternative homeodomain site (TGATTGAT), predominantly found in muscle and thymus cell types (Fig. 4c–g).

Across all 138 de novo composite motifs tested, we identified 67 with significant synergy (Fig. 4h–j and Supplementary Table 7). Hard syntax motifs showed preferred spacings between motif centres around 7 bp and 11 bp—consistent with protein binding in adjacent or helical-turn offset DNA grooves—and exhibited spikes in predicted joint effects at specific arrangements (Fig. 4h,k, Extended Data Fig. 8f,g). By contrast, soft syntax motifs displayed broader distance preferences with more modest joint effects that decayed gradually to independent effects beyond about 100 bp (Extended Data Fig. 8f,g). Among hard syntax motifs, we recovered composite elements consistent with known dimeric transcription factor interactions, including SOX homodimers^45^, p53 homodimers^46,47^ and GATA–TAL heterodimers^48,49^ (Supplementary Note 3). Notably, the optimal spacing and orientation of the predicted p53 homocomposite motif matched the canonical 4 bp separation of its two NCATGN binding sites oriented head-to-tail^46,47^. We highlight several motif pairs with reported synergy but limited prior investigation of their spacing and orientation constraints—including RUNX–RUNX^50^, FOX–NR^51,52^ and ETS–NR^53^—which may represent novel syntax features in human fetal development. Screening all motif pairs across all cell types provided implicit negative controls; our strategy distinguished a small subset (8.9%) of predominantly cell-type-specific synergistic pairs from the mostly non-synergistic motif combinations (Supplementary Note 3).

Synergistic motif syntax was detected across most cell lineages. Approximately 60% of cell types revealed at least one hard syntax and one soft syntax composite motif pattern. Within each cell type, hard and soft syntax composite motifs exhibited higher total contribution scores compared to motifs without synergy and non-composite motifs, with hard syntax motifs also being significantly more prevalent, indicating greater predictive influence on chromatin accessibility (Extended Data Fig. 8h).

We next tested whether cooperative effects were cell-type-specific (Supplementary Note 3) by inserting motif pairs at their optimal arrangement into background sequences and predicted accessibility across all cell types. For example, an IKZF–RUNX composite motif drove accessibility only in a subset of thymus- and spleen-derived immune cell types (Fig. 4l). This pattern was consistent with the expression of IKZF and RUNX family transcription factors in those same cells, suggesting that cooperative effects are also constrained by the availability of constituent factors. Similarly, a FOX–HNF4 composite motif exhibited predicted accessibility specifically in hepatocytes, correlating with restricted expression of select FOX and HNF4 family members in these cells (Fig. 4m). Although limited detection by single-cell RNA sequencing of transcription factor genes that were expressed at low levels precludes comprehensive analysis, these examples suggest that motif cooperativity depends jointly on precise binding site syntax and cell-type-specific transcription factor expression.

## Ubiquitous motifs reduce accessibility

**Fig. 5:**
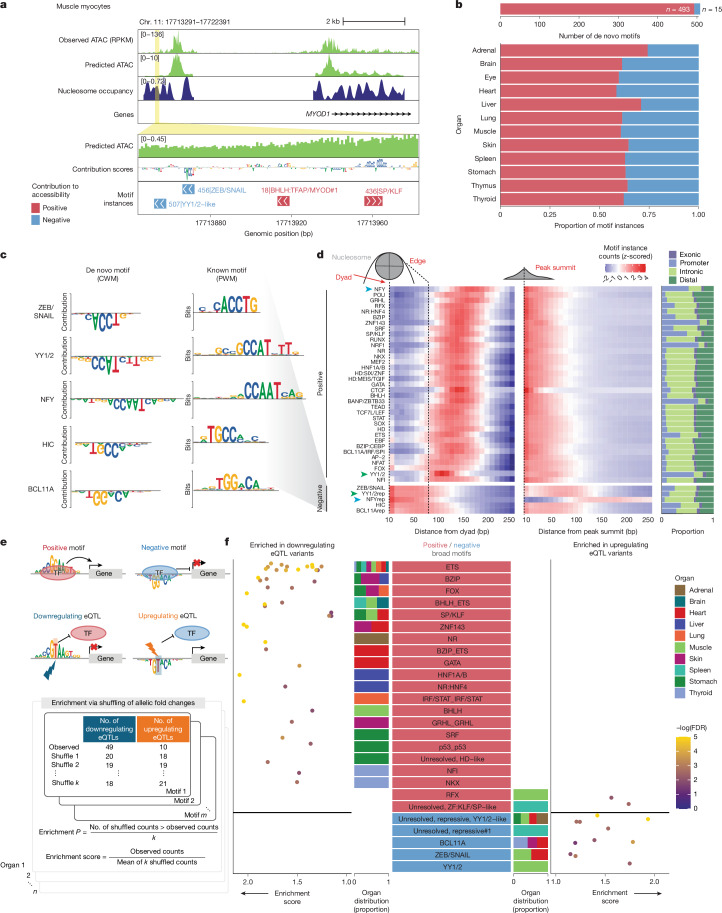
Ubiquitous motifs associated with reduced accessibility and directional eQTL effects. **a**, The *MYOD1* locus in myocytes with observed and predicted chromatin accessibility, inferred nucleosome occupancy, contribution scores and motif instance annotations showing an example of a negatively contributing ZEB/SNAIL motif. **b**, Breakdown of de novo motifs, and breakdown of motif instances for each organ, by positive and negative motifs. **c**, De novo motifs (as CWMs) for each negative motif category, and the most similar known PWM in external databases**. d**, Left and middle, heat maps indicating counts of motif instances in 10-bp bins from inferred nucleosome dyad positions (left) and peak summits (middle), *z*-scored across distance bins per motif in each heat map. Right, proportion of genomic instances overlapping various genomic features. Each row represents a broad group of base motifs, and only groups with at least 50,000 instances across bins are shown. **e**, Workflow for determining tissue-specific enrichment of different classes of fetal motifs with upregulating or downregulating eQTL variants. **f**, Results of eQTL variant analysis. For each broad motif category, points indicate enrichment scores of each unique motif from each organ, coloured by −log_10_ FDR values for downregulating eQTL variants (left) and upregulating eQTL variants (right). Stacked bar plots display the proportion of organs in which motif instances with enrichment originate. Only statistically significant enrichments are shown.

Most de novo motifs had positive contribution scores, but a small subset (15 motifs) were predicted to have negative effects (Fig. 5a,b). Negative motifs were unexpectedly widespread in accessible regions, with 2–5 predictive instances per peak and comprised more than one-third of all predictive motif instances in several organs (Fig. 5b and Extended Data Fig. 5e–g). Many matched known transcription factor families with repressive activity, including ZEB or SNAIL (ZEB/SNAIL), HIC, BCL11A, NFY and YY1/2, although their ubiquitous negative effects on accessibility have not been previously reported (Fig. 5c), whereas others did not match known databases (Extended Data Fig. 6b).

**Extended Data Fig. 9:**
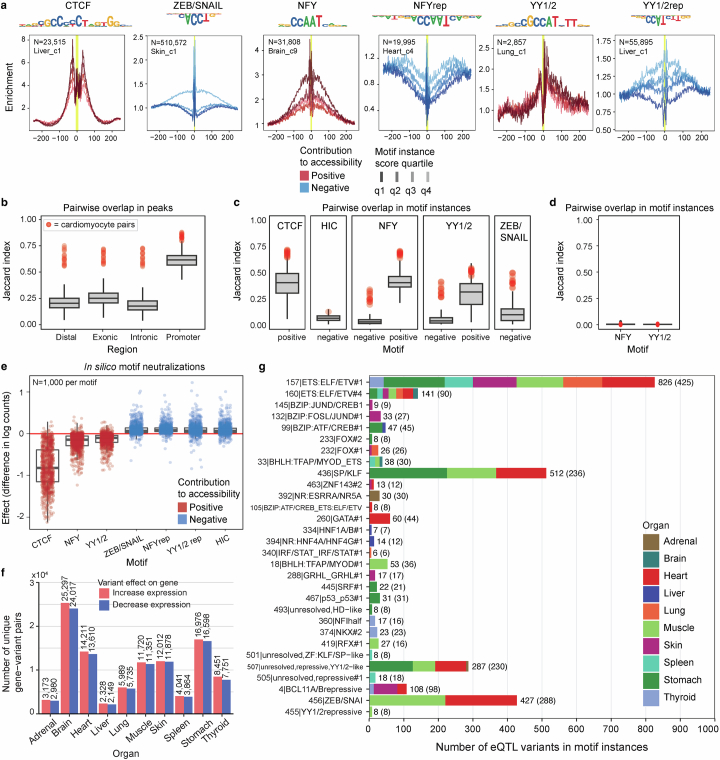
Analysis of negative motifs. **a**) Footprints at ChromBPNet-derived motif instances for select motifs. Motif instances were separated into four quartiles based on the Fi-NeMo hit correlation score, and footprinting performed separately for each. For each motif, footprinting was for the cell type with the most instances of the motif. Enrichment values represent insertion counts at each position within 500 bp windows around the center of each motif instance, divided by the mean insertion count in the outer 10% at each flank. **b-d**) Pairwise Jaccard indices were computed to quantify overlap in genomic regions between cell types. **b**) Pairwise comparisons of overlap in ChromBPNet training peaks, grouped by genomic feature annotation. The cell type pairs with the highest Jaccard indices correspond to different cardiomyocyte clusters. **c**) Pairwise comparisons of overlap in predictive motif instances, grouped by motif, for a select set of motifs. **d**) Pairwise comparisons of overlap in motif instances for the positive NFY motif with motif instances of the negative NFY motif in other cell types, and similarly for YY1/2. **e**) Summary of in silico ablations of motif instances in genomic regions. 1,000 regions per motif were tested, and y-axis represents the difference in predicted log counts between ablated and reference sequences, for one cell type (Heart_c3, fibroblasts), averaged over five folds. **f**) Number of unique eQTL variant-gene pairs examined per organ. **g**) Motifs significantly enriched in eQTL variants. Numbers state the total number of variants within all instances of the respective motif across all organs, with the total number of unique gene-variant pairs in parentheses.

Of note, NFY and YY1/2 motifs exhibit dual roles, with distinct motifs driving either positive or negative effects (Fig. 5c,d). Footprints aggregated across positive motif instances demonstrated canonical signatures, but reflected decreased accessibility at negative instances relative to flanking regions (Extended Data Fig. 9a). Thus, negative instances represent sites of reduced local accessibility within accessible chromatin regions. Negative motif instances showed lower concordance across cell types compared to positive motifs and a promoter element baseline, suggesting cell-type-specific regulation (Extended Data Fig. 9b,c). Overlap between positive and negative instances of the same motif in other cell types was rare, suggesting that distinct loci positively and negatively influence accessibility (Extended Data Fig. 9d). Negative motifs were enriched near nucleosome dyads and depleted near peak summits, in contrast to positive motifs, which tended to avoid dyads and cluster near summits (Fig. 5d and Extended Data Fig. 5j). We next performed in silico ablations of 1,000 motif instances of each motif and found that ablation of positive motifs led to reductions in predicted accessibility, whereas ablation of negative motifs led to small increases in predicted accessibility (Extended Data Fig. 9e). Thus, non-coding regions with identical sequence features can exhibit opposite contributions to accessibility.

To assess motif effects on downstream gene expression, we computed enrichment of fine-mapped variants in expression quantitative trait loci (eQTLs) from the Genotype-Tissue Expression (GTEx) dataset^54^ that overlapped predictive instances of positive and negative motifs from corresponding fetal organs (Fig. 5e and Methods). Variants that disrupt positive motifs should reduce gene expression, whereas variants that disrupt negative motifs should increase expression. For each fetal organ, we quantified enrichment per motif of upregulating and downregulating eQTL gene variant pairs from the relevant GTEx tissues, compared with a shuffled background set. Although a small fraction of eQTL variants overlapped our predictive motif instances (Extended Data Fig. 9f,g), the enrichments were consistent with our hypothesis. eQTLs that overlapped positive motifs were significantly enriched for downregulating variants (two-sided Fisher’s exact test, *P* = 9.91 × 10^−11^, odds ratio (OR) = 0.047), whereas eQTLs that overlapped negative motifs were enriched for upregulating eQTL variants (*P* = 8.01 × 10^−4^, OR = ∞) (Fig. 5f and Supplementary Table 8). We noted tissue-specific patterns, observing enrichment of variants in GATA motifs in heart^55^, HNF4 motifs in liver^56^ and NKX motifs in thyroid^57^ (Fig. 5f).

## Disease variants in regulatory elements

**Fig. 6:**
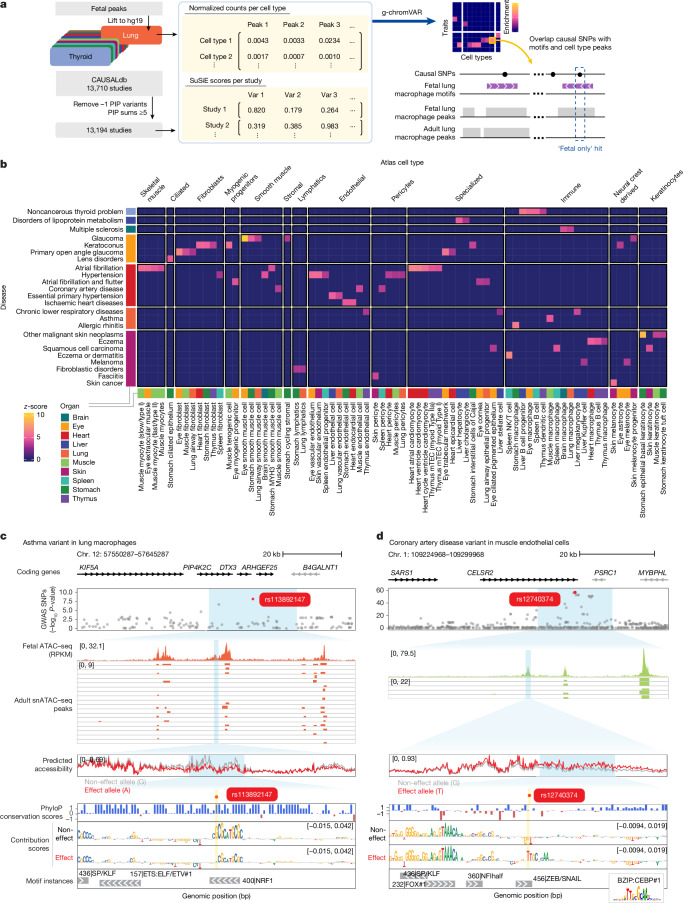
Disease causal variants overlap motifs in fetal-only peaks. **a**, Workflow for the identification of disease-relevant causal variants in motifs that are found in fetal but not adult peak sets. **b**, Top hits from g-chromVAR enrichment results showing only disease traits with the highest average *z*-scores among similar MeSH terms, and L2 fetal cell types with the highest total *z*-scores further grouped by L3 cell type annotations. **c**, rs113892147, an asthma variant, is a fetal-only hit in a positive NRF1 motif in fetal lung macrophages, which is predicted to reduce accessibility. Track are (top to bottom): coding genes, original GWAS *P* values for GWAS single nucleotide polymorphisms (SNPs), observed pseudobulk accessibility for fetal lung macrophage cluster, peaks from single-nucleus ATAC–seq (snATAC–seq) in adult lung macrophages, predicted accessibility for the non-effect and effect alleles, PhyloP conservation scores, per-base pair scores for contribution to predicted accessibility for non-effect and effect alleles, and the motif instances for the non-effect allele. **d**, rs12740374, a CAD variant, is a fetal-only hit overlapping a ZEB/SNAIL negative motif in muscle endothelial cells and predicted to increase accessibility through creation of a C/EBP site (the corresponding motif is shown as an inset at bottom right). Tracks are as in **c**.

Thousands of genome-wide association studies (GWASs) have linked genomic loci to complex phenotypes^58^, but it remains challenging to pinpoint causal variants and the relevant cell types that they affect. We thus interrogated whether accessible chromatin landscapes in fetal cell types are enriched for genetic variants associated with human disease (Fig. 6a). We intersected candidate *cis*-regulatory elements from HDMA with fine-mapped variants from CAUSALdb^59^, which aggregates credible sets across 1,483 GWASs spanning 349 distinct traits.

Using g-chromVAR^60^, we quantified enrichment of trait-associated variants across all fetal L2 cell types and identified 131 cell types with significant enrichment (false discovery rate (FDR) < 0.05) (Supplementary Table 9). To focus on disease-relevant signals, we curated a subset of 68 cell types enriched for variants from 79 studies across 36 Medical Subject Headings (MeSH) disease terms (Supplementary Table 10).

We observed that variant enrichments were predominantly cell-type-specific but organ-agnostic: similar cell types from different organs tended to be enriched for the same disease traits (Fig. 6b). For example, immune cell types were enriched for variants associated with thyroid disease^61^, dermatitis and eczema^62^, rhinitis^63^ and cancer^64^; myocytes were linked to atrial fibrillation risk^65,66^, whereas endothelial cells were enriched for variants associated with hypertensive diseases^67^; and specialized liver parenchymal cells, such as hepatocytes and cholangiocytes, were enriched for variants linked to lipid metabolism disorders^68^.

## Variant effect on local accessibility

To investigate how genetic variants perturb regulation, we prioritized high-confidence causal variants (SuSiE^69^ posterior inclusion probability (PIP) ≥ 0.8) that overlapped predictive motif instances in accessible elements of disease-enriched fetal cell types (Supplementary Table 11). To distinguish fetal-specific effects, we compared variant overlaps with HDMA caCREs against ATAC–seq peaks from matched adult cell types from ENCODE^70^ (Fig. 6a and Supplementary Table 12). We identified 28 hits as fetal-only and 80 hits present in both fetal and adult peak sets.

**Extended Data Fig. 10:**
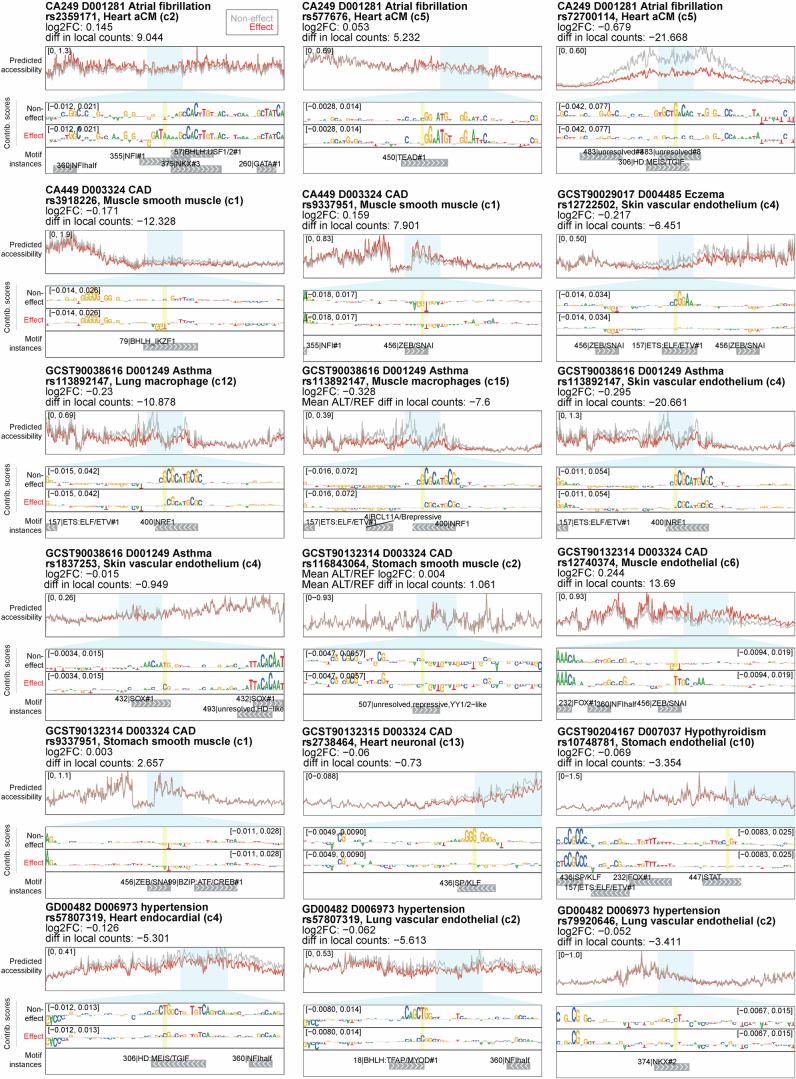
Prediction of variant effects in selected fetal-only hits. For each hit, predicted accessibility for the effect and non-effect alleles are shown for approximately +/− 200 bp around the variant site. Blue highlights indicate the region +/− 30 bp around the variant site displayed in the tracks below. Tracks, in order from top to bottom, represent per-basepair contribution scores for predicted accessibility for non-effect and effect alleles, and motifs called in the respective cell types (with the non-effect allele). Yellow highlights indicate the affected nucleotide. “log2FC” indicates the mean log2 fold change between predicted counts for the effect allele vs the non-effect allele. “Diff in local counts” indicates the difference in predicted read counts between the effect allele and non-effect allele in the 100 bp surrounding the variant. CAD: Coronary Artery Disease.

Using ChromBPNet models, we predicted effects of the 28 fetal-specific variants on local chromatin accessibility (Supplementary Table 13). Many fetal-only variants were predicted to alter accessibility by disrupting motifs (Extended Data Fig. 10). We highlight two such variants (Fig. 6c,d) that involve a positive NRF1 motif (*z*-score = 3.27, FDR = 2.09 × 10^−2^) associated with asthma^71^ in lung macrophages, and a negative ZEB/SNAIL motif (*z*-score = 3.45, FDR = 1.42 × 10^−2^) in muscle endothelial cells associated with coronary artery disease^72^ (CAD).

The asthma-associated fine-mapped variant (rs113892147, SuSiE PIP = 0.819) overlapped a conserved NRF1-binding motif^73^ within an accessible region in fetal lung macrophages (Fig. 6c), but lacked accessibility in adult lung macrophage samples. Installation of the effect A allele was predicted to reduce accessibility relative to the reference allele by the fetal lung macrophage ChromBPNet model, consistent with eQTL data linking the allele to reduced ARHGEF25 expression in adult lung (beta = −0.295,* P* = 6.9 × 10^−7^, Open Targets Platform^74^). Although the direct connection to asthma pathology remains unclear, this result highlights how fetal macrophage enhancers may shape asthma risk.

The CAD-associated variant (rs12740374, SuSiE PIP score = 0.855) overlapped a negative ZEB/SNAIL motif in fetal muscle endothelial cells (Fig. 6d). The effect G allele disrupted the weak negative ZEB/SNAIL motif and created a C/EBP binding site, leading to a predicted increase in accessibility. This locus (CELSR2–PSRC1–SORT1) is well-established in CAD risk^75^, and this variant enhances C/EBP binding and SORT1 expression in hepatocytes^76,77^. Our findings extend its potential role to endothelial cells, consistent with emerging models implicating vascular dysfunction in CAD pathogenesis. Notably, outside of the liver, rs12740374 is associated with increased expression of the nearby *CELSR2* (beta = 0.338, *P* = 1.2 × 10^−36^) and *PSRC1* (beta = 0.238, *P* = 4.6 × 10^−8^) genes in muscle but not in heart ventricle or atrial tissues (Open Targets Platform^74^), further highlighting the potential roles for non-cardiac cells in CAD.

## Discussion

Here we defined chromatin accessibility and transcriptomic landscapes in a multiomic atlas of human development. We resolved organ-specific and cell-type-specific enhancer activity in vivo, used a deep learning strategy to identify regulatory motifs, uncovered distinct modes of transcription factor cooperativity, and interpreted the impact of disease-associated genetic variants on the developmental chromatin landscape. Our work substantially extends the resolution and scope of regulatory mapping efforts in human development by uncovering nucleotide-resolved, cell-type-specific regulatory logic across multiple organs.

Our deep learning framework is complementary to classical motif discovery approaches such as motif enrichment from DNase footprinting^5^ or in vitro binding assays^11,12,78^. First, unlike footprinting methods that require deep sequencing, ChromBPNet models can be trained and interpreted on pseudobulk data from single-cell clusters with modest coverage, enabling robust, cell context-specific motif discovery in primary tissues. Our models learn likely causal sequence features that are directly predictive of chromatin accessibility^17^, and enable in silico perturbations for systematic interrogation of motif syntax, providing mechanistic insights. Our de novo motif analyses are consistent with prior findings that a limited set of core motifs regulate the majority of transcriptional initiation events^37–39,79^.

We revealed 67 motif pairs that exhibit synergistic effects on chromatin accessibility, with 48 showing strong preference for specific spacing and orientation—indicative of hard motif syntax which recalls the classic IFNβ ‘enhanceosome’ model^80^ and the AP-1–IRF4 (AICE) and ETS–IRF (EICE) composite elements with rigid binding architectures^81^. Prior systematic surveys of transcription factor cooperativity using in vitro systems identified similar spacing and orientation preferences^11,12^, but lacked in vivo resolution and often focused on composite motifs with fused and overlapping binding sites. The synergistic, tissue-specific composite motifs predicted by our analysis nominate motifs for mechanistic investigation, and support the view that densely packed, spatially constrained transcription factor interactions are a relatively common regulatory mechanism underlying developmental gene programs.

In parallel, the 27 motifs with soft syntax are consistent with biophysical models for indirect cooperativity, including mass-action models of transcription factor competition with nucleosomes, or recruitment of chromatin remodellers which evict nucleosomes^13,14^, consistent with single-molecule footprinting^14^, CAP-SELEX (consecutive affinity-purification systematic evolution of ligands by exponential enrichment) experiments^11^ and deep learning analysis of ChIP-exo (chromatin immunoprecipitation with exonuclease digestion) data^42^. Such syntactic flexibility may confer greater evolutionary robustness, allowing regulatory elements to maintain function while tolerating alteration in motif spacing, number or orientation.

Interpretation of deep learning models identified negative motifs which reduced accessibility, exhibited distinct positional biases towards nucleosome dyads and distal regions, and were significantly enriched for upregulating eQTL variants. Independent evidence supports the regulatory role of negative motifs: a ZEB2 repressor motif decreased enhancer activity in *Drosophila*^82^; a de novo variant creating a repressive NR2F1 motif reduced reporter expression in mouse brain^17^; and in the developing liver, abundant regions enriched for repressive motifs such as ZEB1, TCF4 and SNAI1 predicted lower enhancer activity and gene expression and were suggested to fine-tune regulation^83^. In our dataset, negative motifs included known repressors and previously uncharacterized sequence elements distinct from silencer elements^84^, suggesting a broader repertoire of repressive signals modulating accessibility. Future work will be needed to identify the proteins binding negative motifs, define their interactions with chromatin remodellers or nucleosomes, and understand how they modulate accessibility and expression.

Our observations that motifs for YY1/2 and NFY can function as either positive or negative regulators of accessibility is consistent with the known dual functions of YY1 as both an activator and repressor depending on context^85^ and the role of NFY as a histone-fold protein that maintains nucleosome-depleted regions at active regulatory elements^86^.

Although eQTL analysis highlighted that disruption of positive motifs typically decreases gene expression, consistent with a loss of activating inputs, two positive motifs—an RFX motif and a SP/KLF-like site—were enriched for upregulating eQTLs. These specific eQTLs may reflect affinity-optimizing variants, leading to enhanced transcription factor binding and gene expression^87^. Alternatively, these transcription factors may increase chromatin accessibility while repressing gene expression. Indeed, RFX family members regulate distinct gene sets across tissues and act as repressors at specific regulatory elements^88^.

Finally, we linked disease-associated variants to specific developmental cell types and predicted their effects on chromatin accessibility. Many variants associated with adult-onset traits were located in fetal-specific accessible regions, suggesting that genetic variants may influence disease by perturbing the development of relevant cell lineages, even if the affected chromatin elements become inaccessible in adult tissues. The asthma-associated variant in fetal lung macrophage suggests that early-acting variants could compromise the development of alveolar macrophages, which originate from fetal liver monocytes and persist into adulthood^89^. Alternatively, some variants may enable aberrant reactivation of fetal regulatory elements later in life and contribute to disease predisposition. These findings underscore the relevance of developmental contexts for disease aetiology.

Our study has limitations. Although our atlas captures a broad range of cell types, deeper sampling will be needed to fully resolve the full diversity in developmental tissues. Our data do not recapitulate organ-specific enhancer activity at all experimentally validated VISTA enhancers, perhaps because these enhancers were only assayed at mouse embryonic day 11.5, which is transcriptionally most similar to human post-conception week 5 (ref. ^90^), much earlier than the samples that we profiled. Moreover, the VISTA collection represents a small set of strong, tissue-specific enhancers, so observations from these elements may not generalize to the full set of annotated caCREs. Deep learning models trained on accessibility primarily capture the influence of sequence-specific DNA-binding proteins that modulate accessibility and may miss regulators that act downstream or independently of accessibility^17^. Furthermore, motif degeneracy within transcription factor families precludes definitive assignment of individual transcription factors to de novo motifs. Finally, although our variant effect predictions offer mechanistic hypotheses, experimental validation will be required to confirm the predicted effects on chromatin state and gene regulation.

Our work represents a comprehensive, multiomic single-cell atlas of human fetal development together with a predictive framework for decoding the *cis*-regulatory syntax that governs chromatin accessibility across cell types. We provide an extensive data resource, trained models, genome browser-compatible tracks and an efficient pre-processing pipeline to enable detailed exploration of regulatory and disease mechanisms across human development.

## Methods

### Ethics statement

De-identified tissue samples were collected at Stanford University School of Medicine from elective termination of pregnancy procedures with informed consent for the research use of tissues in observance of relevant legal and institutional ethical regulations. No demographic information was collected. Consent was obtained by the medical team. The relevant tissue sample processing and analyses were performed under protocol SCRO-796, approved by the Stem Cell Research Oversight Panel (SCRO) at Stanford.

### Sample collection and nuclei isolation

Tissue samples were delivered on ice and immediately stored in liquid nitrogen prior to processing. A multi-tissue compatible nuclei isolation protocol was developed to efficiently isolate stable nuclei for further library preparation. In brief, for a given sample, 100–200 mg of tissue was added directly into 1 ml of Nuclei Extraction Buffer (250 mM Sucrose, 25 mM KCl, 5 mM MgCl_2_, 20 mM HEPES-KOH, 65 mM β-glycerol, 0.5% IGEPAL CA-630, 1× protease inhibitor, 1 mM DTT, 0.2 mM Spermine, 0.5 mM Spermidine, 60 U ml^−1^ RNasin Plus, 2–5% normal goat serum) in a chilled 2 ml dounce homogenizer (Kimble 885300-0002) on ice. The sample was incubated for 10 min on ice. The sample was dounced 20 times each with pestle A then with pestle B. Sample was transferred to a DNA low binding tube. Three hundred µl additional Nuclei Extraction Buffer was used to rinse any remaining nuclei from dounce homogenizer. Sample was incubated with vertical rotation for 5 min at 4 °C. Sample was filtered using a 70-µm Flowmi strainer. Volume was adjusted with additional Nuclei Extraction Buffer to 1.2 ml total volume. Thirty-seven per cent formaldehyde was added to the sample for a 0.2% final formaldehyde concentration and incubated for 4 min at room temperature with vertical rotation. Fixation was quenched with 125 mM glycine for 8 min at room temperature with vertical rotation. Nuclei Extraction Buffer was added to the sample for a final volume of 1.4 ml. An iodixanol gradient was prepared to enrich nuclei from homogenate. In brief, 50% iodixanol solution was prepared from 60% iodixanol with the addition of 1 mM DTT, 60 U ml^−1^ RNasin Plus, and 2–5% normal goat serum. The sample was mixed with an appropriate amount of iodixanol for a final 22% iodixanol concentration. 44% iodixanol solution was layered below the sample. Then, a 22% iodixanol solution was gently added between the sample and the 44% iodixanol solution layer. The sample was centrifuged at 3,500*g* for 30 min at 4 °C with brakes off. The nuclei layer was separated with gentle pipetting for further processing.

### SHARE-seq library preparation

The full protocol is described in Supplementary Note 1, adapted from published SHARE-seq protocols^21,91^. In total, we processed 76 tissue samples derived from 23 individuals, across 12 tissue processing and SHARE-seq library preparation batches, where each batch corresponded to all samples of a given organ.

### Library sequencing

All DNA libraries were sequenced on a NovaSeq 6000 using 300-cycle S4 v1.5 reagent kits with XP workflow. Paired-end sequencing was run with a 96-99-8-96 configuration (Read1-Index1-Index2-Read2). We quantified DNA libraries using Qubit and Tapestation, then prepared library pools at 1.5 nM concentration for a final loading concentration of 300 pM. Sequencing was performed at the Stanford Genome Technology Center.

### VISTA embryo histology

We received X-Gal-stained and fixed whole mouse embryos in PBS from L. Pennachio^26,27^ and transferred them to 70% ethanol for storage. Paraffin embedding was performed by Histo-Tec Laboratory using a xylene-free dehydration protocol as xylene could dissolve the X-Gal stain. In brief, the embryos were sequentially dehydrated with 80%, 95%, 100%, 100% and 100% ethanol for 20 min each, followed by washes with 50:50, 80:20, 90:10 and 100:0 paraffin:alcohol mix for 20 min each to remove the ethanol. Subsequent embedding and H&E staining was performed with standard protocols on 5-μm sections.

### SHARE-seq data pre-processing

We developed a highly parallelized, rapid, and storage-efficient pre-processing Snakemake (v7.15.1)^92^ pipeline to convert BCL files from sequencers to ATAC fragment files and RNA sparse matrices (Extended Data Fig. 1). In brief, raw BCL files were first converted to FASTQ files using a custom script that parallelizes the bcl2fastq (v2.20.0.422, Illumina) conversion by flow cell tiles, parses the read cycles, and demultiplexes the raw FASTQ files into sublibraries based on sublibrary barcodes in the Index2 reads. For each sublibrary, we further split the FASTQ file into random chunks of 20 million reads.

Within each chunk of an ATAC–seq sublibrary, we performed barcode matching against the SHARE-seq barcode whitelist, allowing for 1 bp mismatch for each of the three rounds of 8 bp barcodes that make up a single-cell barcode, followed by Nextera adapter trimming with fastp (v0.23.2)^93^, genome alignment with Bowtie2 (v2.5.0)^94^, and conversion of the output BAM file to a more storage-efficient fragment file. We then merged the fragment files from all chunks of a sublibrary, deduplicated fragments per cell based on start and end coordinates, and demultiplexed the fragments into samples based on round 1 cell barcodes. Finally, for each sample, we merged the demultiplexed fragment files for that sample across all sublibraries to generate the final ATAC–seq fragment files (*.fragments.tsv.gz, *.fragments.tsv.gz.tbi).

Within each chunk of an RNA sublibrary, we performed barcode matching, 10 bp UMI parsing from Read2, and adapter trimming for Read1 only, followed by genome alignment with STAR (v2.5.4b)^95^, gene annotation with featureCounts (v2.0.1)^96^, and conversion of the output BAM file to a more storage-efficient TSV format. We then merged the annotated TSV files from all chunks of a sublibrary, split into 12 barcode chunks based on round 3 barcodes, deduplicated UMIs per cell per annotated gene per barcode chunk using UMI-tools (v1.1.2)^97^, demultiplexed the deduplicated TSV files into samples based on round 1 cell barcodes, and converted the TSV files into the Matrix Market Exchange format. Finally, for each sample, we merged the demultiplexed Matrix Market Exchange files for that sample across all sublibraries to generate the final RNA sparse matrix files (*.matrix.mtx.gz, *.features.tsv.gz, *.barcodes.tsv.gz).

On average, we can process a 10B-read NovaSeq run in under 4 h using an academic high performance computing cluster. This pipeline can be easily adapted to process other split-and-pool-based single-cell multiomic data. All libraries were aligned to the hg38 reference genome. The pipeline is available at https://github.com/GreenleafLab/shareseq-pipeline (stable release v1.0.0). Raw sequencing reads have been anonymized using BAMboozle^98^ prior to public deposition to protect donor privacy. The anonymization code is available on the ‘anonymize’ branch of the shareseq-pipeline GitHub repository.

It is well known that in ATAC–seq experiments, Tn5 transposase forms a homodimer with a 9-bp gap between the two Tn5 molecules, resulting in two insertions 9 bp apart on different DNA strands per accessible site^4,99^. When sequencing the DNA fragments using paired-end sequencing, the start and end positions need to be adjusted based on the insertion offset of Tn5 to reflect the true centre of the accessible site at the midpoint of the Tn5 dimer. To account for the Tn5 offset, previous ATAC–seq studies used a + 4/−5 offset approach where plus-stranded insertions are adjusted by +4 bp, and minus-stranded insertions by −5 bp. However, this in fact results in a 1 bp mismatch of the adjusted insertion sites between the two fragments sharing a single transposition event (Extended Data Fig. 1b). The discrepancy may stem from the end-exclusive coordinate system used by BAM and BED files, as the original +4/−5 convention is only correct if the output file is interpreted in a non-standard 0-based, end-inclusive genomic coordinate system. This mismatch does not affect most downstream ATAC–seq analysis that bins insertions on the scale of hundreds of base pairs, but it does affect base pair-sensitive analysis such as transcription factor footprinting and motif analysis. In this SHARE-seq pre-processing pipeline, we have adopted the +4/−4 offset instead, which results in a consensus insertion site. See example motifs generated from reads corrected by either of these offset schemes in supplementary figure 3a in ref. ^17^.

### SHARE-seq data QC and filtering

We performed per-sample quality control (QC) filtering by manually inspecting and thresholding the following metrics: (1) TSS enrichment ratio and number of fragments for ATAC–seq fragment files; (2) number of UMIs, number of genes and percentage of mitochondrial reads for RNA sparse matrices; (3) ratio of RNA UMIs versus ATAC–seq fragments to remove cells with low quality in one modality (Extended Data Fig. 2a). All sample filtering thresholds are summarized in Supplementary Table 1. No explicit batch effect correction was performed, as individual-specific effects are often confounded with temporal differences in cell type composition, making it challenging to separate these sources of variation.

### RNA normalization, ambient RNA removal, dimensionality reduction and clustering

We used Seurat (v4.3.0)^100^ in R (v4.1.2) to process filtered RNA sparse matrices into Seurat objects per organ^100^. We adopted an iterative dimensionality reduction and clustering workflow to sequentially annotate cell types and filter out additional low-quality clusters (Extended Data Fig. 2a). For each iteration, we first performed SCTransform v2 and variable feature selection on RNA raw counts of each sample, then selected the top 3,000 consensus variable features across samples using the SelectIntegrationFeatures function from Seurat, excluding mitochondrial genes, sex chromosomes genes, and cell cycle genes to minimize batch effects. We merged the raw RNA counts from per-sample objects into a single matrix, performed SCTransform v2 using consensus features, and used the DecontX function from the celda (v1.6.1) package^101^ on SCT-corrected counts to remove ambient RNA contamination per cell. The decontaminated counts were then split by sample, scaled to 10,000 UMIs per cell and log-normalized. Similar to the process mentioned above, we selected a list of top 3,000 consensus variable features from the per-sample variable features. Principal components analysis was performed on the merged object with the consensus features, followed by cell clustering using the Louvain algorithm at a resolution of 0.3 with 50 principal components and uniform manifold approximation and projection (UMAP) embedding. We then inspected each cluster and removed any low-quality clusters with significantly lower UMIs than other clusters, high levels of co-expression for different tissue compartment markers that are biologically impossible and suggestive of doublets (for example, high expression for both epithelial and endothelial compartment markers), or no clear cell type-defining marker genes. After removing cells in the low-quality clusters, we repeated the processing steps starting from RNA raw counts for each sample. This process was repeated until no more low-quality clusters were identified, which usually required one to three iterations. Cells in the final set of clusters passing this iterative QC were considered ‘whitelisted’. For each cell type cluster, marker genes were identified in a one-versus-all Wilcoxon rank-sum test versus all other clusters from the same organ, and filtered to obtain genes with a log_2_ fold change greater than 1.

All final cluster annotations are included as Supplementary Table 2. All cluster markers are summarized in Supplementary Table 3 and a subset is visualized in marker gene dot plots in Supplementary Note 2. All UMAP embeddings are included in Supplementary Note 2.

### ATAC–seq peak calling, motif enrichment and chromVAR

We used ArchR (v1.0.2)^102^ to process filtered ATAC fragment files into ArchR projects per organ. After filtering to the final whitelisted cell barcodes from the iterative RNA processing workflow and transferring the clustering and cell type annotations, we called peaks per cluster using Macs2 (v2.2.7.1)^103^, merged peaks into a single reproducible peak set per organ using ArchR’s iterative overlap strategy, and created a cell-by-peak matrix of fragment counts. We identified marker peaks per cluster using a Wilcoxon rank-sum test and performed transcription factor motif enrichment within the marker peaks with a cutoff of FDR ≤ 0.1 and log_2_(fold change) ≥ 0.5. We calculated chromVAR^22^ motif deviations across all clusters within each organ^22^. For both of these analyses, we used a curated cisBP motif set of 1,141 unique human transcription factor motifs described in refs. ^104,105^. We created a global ArchR project by merging all 12 per-organ ArchR projects and an HDMA global caCRE set by iteratively overlapping peak sets called from individual clusters across all organs^102,106^.

### Linkage of regulatory elements to genes with modified ABC model

We used the ABC approach to link caCREs to gene promoters. To ensure consistency of ABC enhancer regions as those in our HDMA global caCREs set, we adapted ABC^24^ to enable custom regions as inputs to the model. To create the custom region set, we used bedtools to merge the HDMA global caCREs set with the hg38 genome TSS set. Following the recommended scATAC workflow from the official ABC documentation (https://abc-enhancer-gene-prediction.readthedocs.io/en/latest/index.html), we used the pseudobulk ATAC–seq signal (without H3K27ac chromatin immunoprecipitation with sequencing (ChIP–seq)) as enhancer activity and estimated 3D contact frequency between enhancers and promoters using a power law function of genomic distance. ABC was run on each L1 cell cluster. The results were filtered to obtain enhancer–promoter links with an ABC score greater than 0.013, which corresponds to a 70% recall rate from the benchmark CRISPR dataset. Our modified ABC workflow is available at: https://github.com/GreenleafLab/ABC-Enhancer-Gene-Prediction-CustomRegions (commit b3d2156).

### VISTA enhancer analysis

We filtered the results to obtain VISTA-validated enhancers (accessed 24 January 2024) that originated from humans or have a human sequence homologue and annotated as X-Gal positive in any organs present in HDMA, overlapped with HDMA global caCREs, and retained VISTA enhancers with a minimum of 75% (375 bp) overlap. If multiple caCREs overlapped the same VISTA enhancer, we chose the caCRE with the highest ATAC–seq signal, based on previous observations that VISTA enhancers often have a much smaller core element and enhancer activity does not depend on all regulatory elements within an enhancer^107^. ATAC–seq signal was scaled and log-normalized per L1 cluster then *z*-scored across clusters per enhancer. For each organ, a one-sided Wilcoxon rank-sum test was performed to calculate the statistical significance of the HDMA ATAC–seq signal enrichment in caCREs overlapping VISTA enhancers annotated as positive in that organ. For example, to test the significance of brain ATAC–seq signal enrichment, we first subsetted to HDMA brain clusters only, then compared ATAC–seq signal in caCREs overlapping VISTA brain-positive enhancers and caCREs overlapping VISTA brain-negative enhancers using a one-sided Wilcoxon rank-sum test. The effect size was calculated as the *W* statistic/(*n*_1_ × *n*_2_), where *n*_1_ is the number of caCREs in the brain-positive group and *n*_2_ is the number of caCREs in the brain-negative group. This effect size represents the AUROC probability that a given caCRE in the brain-positive group will have higher ATAC signal than a caCRE in the brain-negative group. Similarly, for the RNA data, we first identified the nearest gene for each caCRE overlapping a VISTA enhancer, scaled and log-normalized the raw RNA expression counts per L1 cluster, and then *z*-scored expression values across clusters per enhancer. An analogous Wilcoxon rank-sum test was performed for each organ to assess the statistical significance of the HDMA RNA signal enrichment in VISTA positive enhancers. To avoid numerical underflow to zero at machine precision in Wilcoxon rank-sum tests, *P* values were calculated on the log_10_ scale and reported accordingly.

### Preparation of input regions for ChromBPNet models

To define genomic regions for training ChromBPNet models, we performed a second round of peak calling to obtain a lenient set of accessible regions. First, pseudobulk fragment files for each of the 203 L1 cell type clusters were generated by concatenating fragments from the SHARE-seq ATAC–seq modality for all cells in that cluster, from all samples. For each pseudobulk, we then derived pseudoreplicates. For each fragment, starts and ends (corresponding to Tn5 insertion sites) were randomly allocated to each of two pseudoreplicate files, and pseudoreplicate files were also concatenated into a total-pseudoreplicate file. Macs2 (v2.2.9.1) was used to call peaks on all three pseudoreplicate files with parameters: -p 0.01–shift −75–extsize 150–nomodel -B–SPMR–keep-dup all–call-summits. Only peaks called on the total-pseudoreplicate which overlapped peaks called in both pseudoreplicates were retained. Peaks overlapping the GRCh38 ENCODE blacklist (ENCODE accession ENCFF356LFX) were excluded. Peak coordinates were adjusted to 1,000 bp centred at the Macs2 peak summit. Pseudoreplicates were only used for peak calling, and pseudobulk fragment files were used for downstream model training.

We used the ChromBPNet package (https://github.com/kundajelab/chrombpnet, commit a5c231) and followed the workflow described^17^. We used the command chrombpnet prep nonpeaks to define background regions that match the GC content of peak regions. For each cell type, we used a fivefold cross-validation scheme, where each fold (designated 0 to 4) comprised a different set of training, validation, and test chromosomes, with each chromosome in the test set of at least one fold. We used the default human chromosome folds provided with ChromBPNet^108^.

### ChromBPNet model training

ChromBPNet models are supervised convolutional neural networks trained to use 2,114-bp one-hot-encoded DNA sequence in peaks and background regions to predict the accessibility profile (as a probability distribution) and total natural log counts (as a scalar value) in the central 1,000-bp window of input regions. ChromBPNet models use a pre-trained bias model, and then explain the residual accessibility not captured by Tn5 enzyme bias. We trained a bias model to learn the enzymatic bias in the SHARE-seq setting using fold 0 for the heart L1 cluster 0 pseudobulk, with bias threshold factor -b 0.4 using the chrombpnet bias pipeline which also performs model interpretation using DeepLIFT. We confirmed that the bias model learned the Tn5 motifs but not transcription factor motifs, and used this bias model to subsequently train ChromBPNet models for five folds for each of the 203 L1 cell types (1,015 models total) using the chrombpnet pipeline command with the GRCh38 reference genome from ENCODE (fasta: https://www.encodeproject.org/files/GRCh38_no_alt_analysis_set_GCA_000001405.15/, with chromosome sizes from ENCODE accession ENCFF667IGK).

### ChromBPNet model evaluation

Models were evaluated based on the Pearson and Spearman correlations between predicted and observed log counts in peaks and the Jensen–Shannon distance between predicted and observed profiles in peaks, for peaks on held-out test-set chromosomes (Supplementary Table 5 and Supplementary Note 2). Models for 4 cell types where the Spearman correlation for any fold was less than 0.5, generally corresponding to pseudobulks with low coverage, were excluded. To generate the average predicted accessibility tracks across folds for peak regions (representing counts per base), for each region, the mean predicted profile logits across folds were processed with the softmax function to convert them to probabilities, then scaled by the exponentiated mean predicted log counts across folds.

### ChromBPNet model interpretation

We performed model interpretation to determine the extent to which each nucleotide was predictive for accessibility. We ran the chrombpnet interpret command which uses the DeepLIFT^30^ algorithm to compute contribution scores for each nucleotide in the 2,114-bp input windows with respect to the predicted counts. Contribution scores were derived for each model fold for all peak regions, and the mean computed across folds. The averaged predicted accessibility profiles and contribution scores were converted to bigWig files, as well as used for all analyses and figures.

### De novo motif discovery and assembly of the motif lexicon

Assembly of the de novo motif lexicon required three steps: (1) de novo motif discovery per cell type; (2) collapsing similar motifs across cell types; and (3) motif annotation.

1. First, for de novo motif discovery on sequences driving chromatin accessibility, we used TF-MoDISco^31^, which, in brief, identifies seqlets, corresponding to short spans of contiguous high positive-importance or high negative-importance nucleotides, and clusters them into recurrent 30 bp patterns. We used the implementation in the TF-MoDISco-lite package (https://github.com/kundajelab/tfmodisco, v2.0.7) on the mean contribution scores for each cell type, sampling 1,000,000 seqlets for both positively and negatively contributing seqlets (parameter -n 1,000,000), and using the default behaviour to search for seqlets in the central 400 bp of input regions. Each de novo motif is represented by a 4 × 30 CWM, computed as the mean contribution score per position per nucleotide across its seqlets, and a 4 × 30 position probability matrix (PPM), computed by normalizing the nucleotide frequencies per position across its seqlets. We manually inspected CWMs learned in each cell type, and the ChromBPNet models and motifs for ten cell types which predominantly learned low-complexity motifs were excluded from downstream analysis. This resulted in 189 cell types (945 models total) passing quality control and used throughout our study, which collectively learned 6,362 motifs including both positively contributing and negatively contributing motifs.
2. Next, we automatically consolidated the 6,362 motifs into a non-redundant set. We first derived clusters of motifs which were broadly similar. CWMs were trimmed by removing positions where the total contribution across nucleotides was less than 30% of the maximum total contribution among all positions^17^. PPMs were trimmed to the same positions as the trimmed CWMs, and converted to position frequency matrices (PFMs) by multiplying PPMs by the total number of seqlets associated with each motif. PFMs from all cell types in each organ were first leniently clustered, separately for positive and negative motifs, using the gimmemotifs package^109^ (v0.18.0, with command gimme cluster -t 0.8), which returns an average PFM for each motif cluster. Average PFMs from across organs were then subjected to a second round of clustering with gimmemotifs. Within each broad motif cluster, we then collapsed the constituent CWMs originating from individual cell types using the SimilarPatternCollapser functionality in TF-MoDISco, which merges together similar motifs using the same method it does for seqlets during initial motif discovery in step (1). This procedure resulted in 834 motifs.
3. Finally, we performed motif quality control, and annotated and categorized each motif. For annotation, we used TOMTOM^110^ (v4.11.2) to compute similarities between the 834 de novo motif CWMs and a curated set of 5,193 known transcription factor binding site position weight matrices (PWMs) from JASPAR, CIS-BP, and other studies (obtained from https://resources.altius.org/~jvierstra/projects/motif-clustering-v2.1beta/), using the command tomtom -no-ssc -oc.–verbosity 1 -text -min-overlap 5 -mi 1 -dist pearson -evalue -thresh 10.0. For every motif, we manually inspected the most similar PWMs to assign a provisional label, typically at the transcription factor family level. We further collapsed highly similar motifs missed by the clustering approach, retaining the motif with the highest number of seqlets across cell types. We flagged 107 motifs which were low-complexity, noisy, or dominated by CG dinucleotides for exclusion. We categorized motifs as ‘base’ if they matched known PWMs; ‘base with flanks’ if they matched known PWMs but exhibited additional high-contribution nucleotides; and ‘homocomposite’ or ‘heterocomposite’ if the motifs clearly matched two similar or distinct known motifs, respectively. Motifs that did not resemble known PWMs were labelled as ‘unresolved’. After exclusion of low-quality motifs, this resulted in a set of 508 non-redundant motifs used for downstream analysis (Supplementary Table 6). Motif labels have the naming scheme ‘<unique_ID > |<family > :<subfamily > /<alternative_subfamily > #<index > ’. The unique ID is a value from 0 to 508 (one ID corresponding to the low-quality motifs was filtered out). The index is used to distinguish separate motifs which each match the same known motif, typically representing subtle variations in nucleotide preferences or flanks. Composite motifs used a similar naming scheme, with labels for constituent motifs separated by underscores. Motifs were also assigned a broad label (for example, ‘CTCF’ and ‘CTCFupstream’ motifs shared a broad ‘CTCF’ label), used throughout figures and analyses to aggregate results.

### Identification of predictive motif instances

To identify genomic instances of de novo motifs in each cell type, we used the Fi-NeMo package (https://github.com/kundajelab/finemo_gpu, v0.23, commit b81876d), which performs motif scanning in a contribution-aware manner. In brief, Fi-NeMo fits a sparse linear regression model for each peak region to minimize the difference between the contribution scores in each region, and a reconstruction of the scores from a weighted combination of trimmed input CWMs. In the coefficient matrix, a non-zero coefficient at a certain location indicates a motif instance in that location. For each cell type, the output of Fi-NeMo is a BED file of predictive instances across all peaks, representing short regions which both match motifs by sequence and have relatively high absolute contribution scores. We first ran Fi-NeMo with all 834 clustered CWMs, with parameters –alpha 0.8 –trim-threshold 0.3 and defaults otherwise (parameter alpha is now known as lambda). Due to the competitive nature of motifs in the linear modelling approach, we ran Fi-NeMo a second time with a reduced set of 436 motifs after excluding composite motifs. We performed post-hoc filtering of motifs to obtain a high-confidence annotation for downstream analysis. To evaluate the quality of instance calls for a given motif, Fi-NeMo computes the correlation between the input CWM and the CWM derived from averaging contribution scores for all Fi-NeMo-identified instances for that motif. For each cell type, instances from the two Fi-NeMo runs were concatenated, and motifs where correlation between the instance-CWM and the input CWM was less than 0.9 were flagged. Next, all instances of these flagged motifs were filtered out. Finally, to reduce redundancy, if multiple instances with the same annotation overlapped by more than 3 bp, only the instance with the highest ‘hit_correlation’ value was retained, representing the instance with the contribution scores having the highest similarity with the corresponding input CWM. This step resulted in final motif annotation for each cell type consisting of their predictive motif instances.

### Inference of nucleosome positions from ATAC modality

We used NucleoATAC^34^ to infer nucleosome position and occupancy from the SHARE-seq ATAC modality data. In brief, to determine nucleosome occupancy, NucleoATAC models the observed size distribution of ATAC fragments as a mixture of nucleosomal and nucleosome-free fragments, and the maximum likelihood fraction of nucleosomal fragments at a given position is used as a continuous occupancy signal. We adapted the original code to take fragments as input (https://github.com/sjessa/NucleoATAC, v0.4.1), and ran the NucleoATAC workflow for each cluster pseudobulk fragment file in the same peak regions used to train ChromBPNet models. The outputs of the nucleoatac occ command were used downstream: the nucleosome dyad position calls were used for analysis of motif instances, and the per-nucleotide maximum likelihood fraction of nucleosomal fragments were used for visualization of nucleosome occupancy as genomic tracks.

### Annotation of motif instances

To annotate predictive motif instances with respect to genomic features, instances were assigned as occurring in promoters if they were within 2 kb upstream or downstream of TSSs, exonic if they overlapped exons, intronic if they overlapped gene bodies but not exons, and distal otherwise. Genomic features definitions were based on the Bioconductor TxDb.Hsapiens.UCSC.hg38.knownGene (v3.14.0) annotation, corresponding to the UCSC knownGene track from GENCODE V38, and assembled using the createGeneAnnotation function in the ArchR package. Motif instance distance to TSS was computed as the distance between instance centre positions and TSS defined in the same annotation. Similarly, for each cell type, motif distance to the nucleosome dyad or peak summit was computed by calculating the distance between instance centre positions and the nearest dyad inferred with NucleoATAC, or the peak summit, respectively. For analysis, we counted motif instances in 10 bp bins from 0 to 250 bp from the dyad or peak summit.

### Motif co-occurrence analysis

To identify co-enriched motifs, we tested for pairwise motif co-occurrence enrichment within the ChromBPNet training peaks for each cell type. We restricted the analysis to the set of base motifs within the de novo compendium, and grouped motifs by their broad annotations. For each motif pair, in each cell type, we populated a 2 × 2 contingency table with the number of peaks containing both, one, or neither motif. We then performed a one-sided Fisher’s exact test to calculate a *P* value for enrichment. To correct for multiple hypothesis testing across all pairs, we adjusted the *P* values using the Benjamini–Hochberg method. Significant co-occurrence was assigned for motif pairs with adjusted *P* values < 0.05.

### In silico marginalizations to assess motif synergy

We used the trained ChromBPNet models to assess motif synergy following previous approaches^19,42^. In this in silico marginalization strategy, the predicted effect of a short sequence on chromatin accessibility is quantified by inserting the sequence into a library of background, non-accessible genomic regions (replacing the central nucleotides); and predicting accessibility for each background and edited region with a forward pass through a trained ChromBPNet model. By averaging the difference in predicted natural log counts between the edited and background regions over many regions, we estimate the ‘marginal’ effect of a sequence. Two or more sequences can be inserted to estimate joint effects of those sequences on accessibility. Specifically, for two motifs A and B, we define the predicted log counts for a region with one motif or both inserted as ${y}_{{\rm{A}}}$, ${y}_{{\rm{B}}}$ and ${y}_{{\rm{j}}}$ respectively; and the predicted log counts for an unedited background region as ${y}_{0}$. The marginal effects, in log counts, of motif A and B are ${\Delta }_{{\rm{A}}}={y}_{{\rm{A}}}-{y}_{0}$ and ${\Delta }_{{\rm{B}}}={y}_{{\rm{B}}}-{y}_{0}$, respectively, and their joint effect is ${\Delta }_{{\rm{J}}}={y}_{{\rm{J}}}-{y}_{0}$. We define the independent effects of motifs A and B as ${\Delta }_{{\rm{S}}}={\Delta }_{{\rm{A}}}+{\Delta }_{{\rm{B}}}$, corresponding to a log-additive model for independent effects, or multiplicative model in units of counts. Synergy can then be defined as a significant increase in the joint effects ${\Delta }_{{\rm{J}}}$ of two sequences relative to their independent effects ${\Delta }_{{\rm{S}}}$.

To implement the synergy analysis, we used the tangermeme package^41^ (https://github.com/jmschrei/tangermeme, v0.4.3). We first filtered the set of de novo composite motifs such that each composite was composed of a unique pair of constituent motifs, obtaining 138 composite motifs. For each constituent, we identified the base de novo motif with the highest number of motif instances, trimmed the CWM as above, and defined the consensus sequence as the nucleotide with the highest contribution score at each position in the trimmed motif. The motifs and associated sequences tested are presented in Supplementary Table 7. Sequences were manually adjusted to further remove uninformative flanks or better match the composite motif, and deduplicated so that a pair of the same sequences was only tested once. One hundred background regions were randomly selected from GC-matched background regions for each cell type. For a pair of two of the same motifs, there are three unique orientations, and for a pair of two distinct motifs, there are four unique orientations. We considered each combination of orientation and distance between motifs an ‘arrangement’ of motifs. For each composite motif, the constituent motif sequences A and B were inserted at all possible orientations and distances (from 0 to 200 bp) in the 100 background sequences, and accessibility predicted for background and edited sequences using all five ChromBPNet model folds (for the cell type with the most predictive instances of that composite motif) to compute ${\Delta }_{{\rm{J}}}$ (Supplementary Note 3). Similarly, A and B were inserted alone to compute independent and sum of independent effects ${\Delta }_{{\rm{A}}}$, ${\Delta }_{{\rm{B}}}$, and ${\Delta }_{{\rm{S}}}$. Effects are computed for each sequence and model fold and the mean effect is reported (Supplementary Table 7). For joint effects, the effect of the motif pair at their optimal arrangement (that is, the combination of orientation and distance with the greatest mean effect ${\Delta }_{{\rm{J}}}$) is reported. We considered each motif pair at their optimal arrangement and used a Wilcoxon signed-rank test to test whether the paired differences in joint and independent effects at that arrangement (${\Delta }_{{\rm{J}}}-{\Delta }_{{\rm{S}}}$) were significantly greater than 0. Multiple testing correction was performed using the Benjamini–Hochberg method. Composite motifs with adjusted *P* value < 0.001 and ${(\Delta }_{{\rm{J}}}-{\Delta }_{{\rm{S}}})$ > 0.15 were annotated as synergistic. To confirm that inserted sequences were driving the predicted synergistic effects, we performed model interpretation using DeepLIFT as above on edited sequences, and verified that the sequences predictive of accessibility corresponded to the sequences we inserted. This identified 18 composite motifs where predicted effects were driven by different nucleotides than the ones inserted; and we abstained from classifying synergy for these motifs, resulting in 120 motifs with synergy classifications (Supplementary Table 7).

To define synergistic motifs with syntax preferences (that is, with synergy limited to or increased at specific binding site arrangements), for each composite motif, we computed *z*-scores across the joint effects ${\Delta }_{{\rm{J}}}$ at all arrangements. Composite motifs that had any arrangement with an effect greater than four standard deviations from the mean (*z*-score > 4) were annotated as having hard syntax. We also considered that motifs could have weaker long-range preferences, or soft syntax. Composite motifs with ${(\Delta }_{{\rm{J}}}-{\Delta }_{{\rm{S}}})$ > 0.15 at any arrangement where constituent motifs were between 20 and 150 bp apart were annotated as having soft syntax.

To validate the specificity of our predictions, we repeated the in silico marginalization experiments for the 138 motif pairs in all 189 cell types with ChromBPNet models passing QC (Supplementary Note 3). For each cell type, sequences were inserted into its respective background regions, and mean effects were computed across 100 sequences and 5 model folds. Effects were computed as above, and multiple testing correction was performed using the Benjamini–Hochberg method. To assess cell-type specificity of predicted synergistic effects, for select composite motifs, we analysed the in silico marginalization for the optimal arrangement across all cell types.

### In silico motif ablations

Similarly, for select motifs, we implemented in silico ablations using tangermeme, using trained ChromBPNet models for one cell type (Heart_c3, fibroblasts). For each motif, we computed quartiles of the motif instances based on the Fi-NeMo hit correlation score, and randomly sampled 250 motif instances from each quartile (1,000 sequences total). To ablate motifs in silico, we ‘neutralized’ each instance by replacing the base at each position with the base that had the smallest absolute value contribution score in the hypothetical DeepLIFT scores—that is, the most neutral base. The hypothetical contribution scores are counterfactual estimates of the contributions of all three alternate bases at each position, had it been observed in the sequence context, and are computed using DeepLIFT as part of the ChromBPNet model interpretation workflow.

### Ranking of motifs by prevalence and importance

To assess motif prevalence and importance, we focused on motifs learned in each cell type in the cell-type-specific TF-MoDISco motif discovery step, and only considered positive motifs. To compute motif prevalence, for each cell type, we obtained the set of motifs learned in that cell type, and the number of motif instances in that cell type for the corresponding lexicon motifs. We ranked motifs within the cell type based on their number of instances, and normalized ranks by dividing each rank by the maximum rank in that cell type such that normalized ranks fell in the range [0, 1]. Similarly, to compute motif importance, for each cell type, we obtained the set of motifs learned in that cell type and summed the contribution scores across nucleotides and positions for the corresponding trimmed CWM. Finally, to compare prevalence and importance of different classes of motifs defined in the synergy analysis, we grouped motifs based on whether they had hard syntax, soft syntax, no predicted synergy, or were not tested (meaning the motif was not a composite motif, or filtered out of the synergy analysis as described above). Motifs with both hard and soft syntax were grouped with hard syntax motifs. We then computed the mean normalized rank across motifs in each group, within each cell type.

### Motif footprinting

For select broad motifs (CTCF, ZEB/SNAIL, NFY, the NFY negative motif, YY1/2 and the YY1/2 negative motif), we used our deep learning-derived motif instances, filtered to the most common variant of the motif, and then split instances into quartiles based on the Fi-NeMo hit correlation score. To compute motif footprint metaplots from the SHARE-seq ATAC modality, we used the BPCells footprint function, which counts insertions (that is, fragment starts and ends) that fall within each position in 500-bp windows centred at motif instances. The insertion counts at each position are then divided by the mean insertion count in the outer 10% on each side of the window, taken as a measure of the local background accessibility.

### Enrichment of eQTL variants in motifs

We obtained tissue-specific GTEx v8 eQTL data^54^ and concatenated them by organ source to match our fetal organs, aggregating the tissues as follows: adrenal gland, brain (amygdala, anterior cingulate cortex BA24, caudate basal ganglia, cerebellar hemisphere, cerebellum, cortex, frontal cortex BA9, hippocampus, hypothalamus, nucleus accumbens basal ganglia, putamen basal ganglia, spinal cord cervical c-1, substantia nigra), heart (artery aorta, artery coronary, atrial appendage, left ventricle), lung, liver, muscle (artery tibial, skeletal), skin (not sun exposed suprapubic, sun exposed lower leg), spleen, stomach/oesophagus (stomach, oesophagus gastro-oesophageal junction, oesophagus mucosa, oesophagus muscularis) and thyroid. We obtained unique lists of variant-gene pairs per organ by selecting the variant with the higher posterior inclusion probability score in case of duplicates.

For each organ, the log_2_ allelic fold change effect sizes (aFCs) for each variant-gene pair was used to determine the direction of variant effect on gene expression (upregulating or downregulating expression). Separately for each organ, we concatenated all motif instances from each cell type, then deduplicated entries with identical motif names and genomic positions. The number of upregulating and downregulating variants that overlapped positive or negative motif instances from the matched fetal organ were counted separately to obtain observed counts. aFCs were then randomly shuffled and direction of effect reassigned before the counting was repeated. We performed 100,000 shuffles per organ. Enrichment scores were defined as observed counts divided by the mean of the 100,000 shuffled counts. Enrichment *P* values were calculated as the proportion of shuffles where shuffled counts were larger than observed counts. Multiple testing correction using the Benjamini–Hochberg method was applied to the *P* values and a motif was considered significantly enriched with upregulating or downregulating variants if the FDR was <0.05 and the observed count was above the 95% confidence interval of shuffled counts for that type of eQTL variant. Two-sided Fisher’s exact tests were performed separately for positive and negative motifs to compare the number of motifs with or without significant enrichment with upregulating or downregulating eQTL variants (Supplementary Table 8).

### Enrichment of cell types with disease variants using g-chromVAR

We used g-chromVAR^60^ to compute cell-type-specific enrichment of disease variants. As all genetic variants in CAUSALdb^59^ are reported in hg19 coordinates, ChromBPNet model input peak sets from fetal cell types were lifted over from hg38 to hg19 coordinates, and these were used as input to g-chromVAR. All coordinate conversions were performed using liftOver^111^ as implemented in the rtracklayer (v1.54.0) R package^112^ and the hg38ToHg19.over.chain.gz or hg19ToHg38.over.chain.gz chain files obtained from the UCSC Genome Browser. SuSiE scores for each variant listed in CAUSALdb were separately collated for all 13,710 studies in the database. CAUSALdb contains variants where linkage disequilibrium information was missing in some GWASs, which affects the causal variant estimation in the process of fine-mapping. These variants were given default PIP values of −1 in the database and were removed from our analysis. We separately ran g-chromVAR (v0.3.2) for each organ to obtain *z*-scores and *P* values of cell type enrichments with credible variants per study. We used the PIP values generated by SuSiE^69^ and filtered out studies where the sums of the PIP values across credible variants in that study were <5 to remove studies wherein we would be underpowered, retaining results from 13,194 studies. Multiple testing correction using the Benjamini–Hochberg method was applied to all *P* values and a cell type was considered significantly enriched with variants from a particular study if the FDR was <0.05. We next manually extracted the subset of the significant results corresponding to disease traits relevant to the organs in HDMA (see Supplementary Table 10 for the list of studies). Potentially causal variants (SuSiE PIP ≥ 0.8) were lifted over from hg19 to hg38 coordinates and overlapped with fetal motif coordinates. For each fetal cell type in our dataset, we then manually identified matching adult tissues and cell types with snATAC–seq data in ENCODE, and obtained their snATAC–seq pseudoreplicated peak sets from ENCODE^70^ (Supplementary Table 12). Causal variants overlapping fetal motif instances were subsequently overlapped with the relevant adult peak sets (peaks with −log_10_ *P* value > 2) if available. Only variants found in fewer than two matched adult peak sets were considered as ‘fetal-only’ hits.

### Prediction of variant effect using ChromBPNet models

We predicted and interpreted effects of specific non-coding variants on chromatin accessibility using trained ChromBPNet models, as we have done previously^7^. We used the tangermeme package for predictions and model interpretation. For each variant, we used the 1,000 bp model training peaks for the relevant cell type to extract the reference genome sequence for the peak which the variant overlapped. This sequence (extended equally on either side to 2,114 bp) was fed to all fivefold trained ChromBPNet models for the relevant cell type, to obtain predicted accessibility profile and aggregate log counts in the peak. For each fold, to transform predicted profile logits into accessibility profiles, the profile logits were softmaxed and scaled by the exponentiated predicted log counts; and model interpretation with respect to the counts output was performed using DeepLIFT. Next, the effect allele was substituted into the sequence at the variant position, and predictions and contribution scores were obtained as for the reference sequence. For each model, we computed the variant effect as the sum of differences in per-base predicted read counts in the 100 bp window centred at variant, and computed the mean effect score across folds. We also computed the log2 fold change between predicted counts for the effect versus the non-effect allele for the peak region, where a log2 fold change >0 indicates the effect allele was predicted to increase accessibility. In figures, the mean predicted profiles and contribution scores across folds are shown.

### Genome browser visualizations

For all data visualization at specific genomic loci, we used the BPCells R package^113^ (https://github.com/bnprks/BPCells) along with custom scripts included in our code repository. All genomic tracks are hosted online for interactive visualization with the WashU Genome Browser (https://epigenomegateway.wustl.edu/browser2022/?genome=hg38&hub=https://human-dev-multiome-atlas.s3.amazonaws.com/tracks/HDMA_trackhub.json).

### Statistics and reproducibility

No statistical method was used to predetermine sample size. No data were excluded from the analyses. The experiments were not randomized. The investigators were not blinded to allocation during experiments and outcome assessment. For box plots throughout the figures, the elements represent the following: centre line, median; box limits, upper and lower quartiles; whiskers, minima and maxima that are no further than 1.5× interquartile range.

### Reporting summary

Further information on research design is available in the Nature Portfolio Reporting Summary linked to this article.

## Online content

Any methods, additional references, Nature Portfolio reporting summaries, source data, extended data, supplementary information, acknowledgements, peer review information; details of author contributions and competing interests; and statements of data and code availability are available at 10.1038/s41586-026-10326-9.

## Supplementary information

Supplementary Notes

Reporting Summary

Supplementary Tables Supplementary Tables 1–14

## Data Availability

All processed data (including fragment files, counts matrices, cell annotations, global caCRE annotations, ChromBPNet models, motif lexicon, motif instances, and genomic tracks) are deposited at https://zenodo.org/communities/hdma. Raw, anonymized sequencing data have been deposited to the Sequence Read Achive under BioProject PRJNA1402391. Metadata for raw genomic data produced in our study has been deposited on Zenodo at 10.5281/zenodo.17259745 (ref. ^114^). A description of all data types and the corresponding URLs is provided in Supplementary Table 14. ENCODE v4 cCREs were downloaded from the publicly available database at https://downloads.wenglab.org/Registry-V4/GRCh38-cCREs.bed.

## Code availability

All analysis code is available at https://github.com/GreenleafLab/HDMA and archived on Zenodo at 10.5281/zenodo.17298234 (ref. ^115^).
