# Supplementary material for: Multiomics and deep learning dissect regulatory syntax in human development
Source: Nature. 2026 Apr 8;653(8116):1240–53. doi: 10.1038/s41586-026-10326-9 (PMC13216069; doi:10.1038/s41586-026-10326-9)
Supplement: Supplementary file 1 — Supplementary Notes [file 41586_2026_10326_MOESM1_ESM.pdf]

nature portfolio
https://doi.org/10.1038/s41586-026-10326-9

Supplementary information

Multiomics and deep learning dissect regulatory syntax in human development

In the format provided by the authors and unedited

Supplementary Note 1: SHARE-seq protocol

General notes

- Before starting the experiment, clean materials with RNase Away and 70% ethanol, use autoclaved tips and tubes. Maintain samples and solutions ice-cold during the whole experiment if not specified otherwise.
- Default spin for cells at 500xg for 5 min w/ swing bucket centrifuge.
- Start with 100k-1M cells for fixation (more the better but ensure enough combinatorial space for final sublibraries)
    - Expect ~20% of original starting number of cells by library prep stage.
- Resuspend oligos to appropriate concentrations in IDTE buffer:
    - 1mM = linker oligos
    - 100μM = RT primer, TSO, barcoded oligos
- Add all RNase inhibitors ***fresh*** to mastermix***.***
- Enzymatics RI is the only RNase Inhibitor that seems to be compatible with Tn5 activity, so we switch cells from the PBS-2RI into PBS-RI buffer before ATAC.
- Some washing steps specifies keeping pellet/beads stable and adding buffer directly ***without*** disturbing the pellet/beads. This does affect final recovery.
- When removing supernatant, use a two-step removal method: first remove the majority of supernatant with P1000, and then remove the remaining amount with P200. It may help to tilt the 2mL tube completely horizontally as you get close to removing all the liquid to avoid removing the pellet while removing as much liquid as possible.
- 2mL lo-bind Eppendorf tubes has better cell/nuclei recovery than 1.5mL tubes only when centrifugation is performed in a swing bucket because it's easier to completely aspirate the supernatant from 2mL tubes without disturbing the pellet at the tip of the 2mL tube.
- We split the bulk ATAC and RT reaction per sample into smaller tubes because the reaction efficiency is better with a smaller volume (even under the same concentration of reaction components).
- Where possible, check cell/nuclei quality and count using Countess at every major steps (ideally: pre-fix, pre-ATAC, pre-splitpool, post-ligation. If necessary, can get away without counting at pre-fix).
- Check before every run to ensure sufficient reagents in stock.

Stock Buffer recipes:

These buffers can be made in big batch ahead of time and stored for a few months

Nuclei Isolation Buffer (NIB) (Keep at 4˚C)

Stock solution	Final conc.	Vol to add (mL)
1M Tris HCl **pH 7.5**	10mM	0.5
5M NaCl	10mM	0.1
1M MgCl2	3mM	0.15
10% NP40 (IGEPAL CA-630)	0.1%	0.5
Ultrapure water		48.75
	Total	50

2x RCB (Keep at room temp)

Stock solution	Final conc.	Vol to add (mL)
1M Tris HCl **pH 8.0**	100mM	1
5M NaCl	100mM	0.2
20% SDS	0.4%	0.2
Ultrapure water		8.58
	Total	10

2x BW (Keep at 4˚C)

Stock solution	Final conc.	Vol to add (mL)
1M Tris HCl **pH 8.0**	10mM	0.5
5M NaCl	2M	20
0.5M EDTA	1mM	0.1
Ultrapure water		29.4
	Total	50

1x B&W-T (Keep at 4˚C)

Stock solution	Final conc.	Vol to add (mL)
1M Tris HCl **pH 8.0**	5mM	0.25
5M NaCl	1M	10
0.5M EDTA	0.4%	0.05
10% Tween 20	0.05%	0.25
Ultrapure water		39.45
	Total	50

IDTE (oligo resuspension) (Keep at room temp)

Stock solution	Final conc.	Vol to add (mL)
1M Tris HCl **pH 8.0**	10mM	0.5
0.5M EDTA	0.1mM	0.01
Ultrapure water		49.5
	Total	50

STE (Keep at room temp)

**Stock solution**	**Final conc.**	**Vol to add (mL)**
1M Tris HCl **pH 8.0**	10mM	0.5
5M NaCl	50mM	0.5
0.5M EDTA	1mM	0.1
Ultrapure water		48.9
	Total	50

2x TD buffer (Keep at -20C)

**Stock solution**	**Final conc.**	**Vol to add (mL)**
1 M Tris-HCl pH 7.6	20 mM	2 mL
1 M MgCl2	10 mM	1 mL
*If preparing Tris from solid base, adjust pH to 7.6 before the addition of DMF		
Dimethyl Formamide (DMF)	20%	20 mL
Ultrapure water	NA	Bring up to 100 mL
	Total	100 mL

Split pool oligo plate preparation:

Can be made in big batch ahead of time and stored at -20C for a few months

1. Thaw Round1, 2, 3 barcode oligo plates at room temp and let completely thaw (few hours or also try water bath).
   a. Very critical step that all oligos are at room temp before use.
   b. Spin the plates down using a plate adapter on a swing bucket or plate-compatible centrifuge.

2. Prepare each linker oligo dilution in tubes as below:
   a. 120μL Round 1 linker oligo (1mM) + 11,880μL STE buffer
   b. 120μL Round 2 linker oligo (1mM) + 9,480μL STE buffer
   c. 144μL Round 3 linker oligo (1mM) + 9,360μL STE buffer

3. Dispense following amounts of linker oligos into each 96 well PCR plate:
   a. 90μL diluted Round 1 linker oligo
   b. 88μL diluted Round 2 linker oligo
   c. 86μL diluted Round 3 linker oligo

4. Use a multichannel pipette to transfer the barcode oligo plates to the appropriate Round linker plate:
   a. 10μL Round 1 barcode oligos
   b. 12μL Round 2 barcode oligos
   c. 14μL Round 3 barcode oligos

5. Seal plates with aluminum adhesive cover and ensure that each well is well protected.

   Set up thermocycler as below:

Temp	Time
95˚C	2:00 min
-1˚C/1 min	
20˚C	2:00 min
4˚C	Hold
Total	~1h 26min

6. Check each well to make sure that there was not significant evaporation. If so, add appropriate amount of water to compensate.

7. Aliquot 10μL of the annealed oligos to a new plate (should be enough for 9x plates for each round). Use a liquidator or a liquid handling platform for distribution. Seal carefully to ensure proper seal at each well with aluminum foil seal and store at -20˚C.

Buffer preparation

Prepare following buffers fresh on the day of experiment, keep on ice unless otherwise specified.
Recommended order to prepare buffers: PBS-2RI, 2x TB Omni, 1x TB Omni, PBS-RI, NIB-RI.

Scale according to the number of samples and number of reactions needed

**PBS-2RI**	**Vol for 1 sample (µL)**
1xPBS	3000
7.5% BSA	16.05
Enzymatics RI	7.5
SUPERase RI	3.75
Total	3027.3

**PBS-RI**	**Vol for 1 rxn (µL)**
1x PBS	5
Enzymatics RI	0.0125
Total	5.0125

**NIB-RI**	**Vol for 1 sample (µL)**
NIB	8323
Enzymatics RI	21
SUPERase RI	21
Total	8365

*6.565mL fixed volume of NIB-RI required given round 1 barcoding uses a single 96 well plate, another 1.8mL per sample required

**2x TB (Omni)**	**Vol for 1 rxn (µL)**
0.2M Tris-acetate	8.25
5M K-acetate	0.66
1M Mg-acetate	0.5
10% Tween-20	0.5
1% Digitonin	0.5
100% DMF	8
Ultrapure water	6.59
Total	25

ATAC (~20min)

* Includes time-sensitive steps, make sure the appropriate buffers are ready ahead of time.
* This protocol assumes nuclei isolation, permeabilization, and fixation has already occurred during tissue dissociation.

1. Set swing bucket centrifuge to 4C.
2. Prepare 1xTB:

**1x TB Omni**	**Vol for 1 rxn (µL)**
2x TB (Omni)	25
Ultrapure water	16.45
PIC	0.2
Enzymatics RI	0.85
Total	42.5

3. After appropriate nuclei dissociation protocol and fixation, mix the desired amount of cells with 500µL of PBS-RI and mix thoroughly, especially if it contains iodixanol.
4. Spin down at 500xg for 5 min at 4C in swing bucket centrifuge.
5. Prepare bulk transposition as follows, each reaction is ~40k cells. Prepare just enough based on the total number of ATAC reactions needed.

**ATAC rxn**	**Vol for 1 rxn (µL)**
Sample resuspended in PBS-RI	5
1xTB (Omni)	42.5
SHARE-ATAC Tn5 WJG Mar 2023	2.5
Total	50

   a. Resuspend each sample pellet in PBS-RI in appropriate volumes (5 µL per ATAC reaction).
   b. Assemble the ATAC reactions per sample with appropriate volume of 1xTB and Tn5. Tn5 is viscous and should be pipette mixed in slowly.
   c. Mix well and distribute 50µL per well to a lo-bind 96 well plate on a cooling block on ice (it’s ok if the last well gets <50µL). Keeping plate cold is essential to control the Tn5 activity before the reaction starts. Seal plate, and incubate at 37˚C for 30 min with shaking at 500 rpm.
   d. At this time, thaw the 5x RT buffer, RT primer (100µM), and dNTPs.
6. Pool ATAC reactions by sample in 2mL lo-bind tubes, keeping the plate and tubes on ice as much as possible.
7. Spin down at 500xg for 5 min at 4C, remove supernatant.
8. Wash pellet gently ***without*** resuspending with 1mL NIB-RI.
9. Spin down at 500xg for 5 min at 4C.
10. Remove supernatant and resuspend pellet ***thoroughly*** in appropriate volumes of EB per sample (10µL per RNA RT reaction).

<u>RT (~45 min)</u>

1. Prepare RT mix (optimized for 100k cells per 50µL rxn):

**RT mix**	**Vol for 1 rxn (µL)**
5x RT buffer	10
Ultrapure water	1.56
dNTPs	2.5
Enzymatics RI	0.31
SUPERase RI	0.63
RT primer (100µM)	5
50% PEG (wide bore tips)	15
Maxima H Minus RT (add right before rxn)	5
Total	40

2. Start the RT protocol on thermocycler – the protocol should have a 50˚C hold so that when the samples are put in the machine, the hold is released and the 10min at 50˚C can be started right away.
3. Add appropriate volumes of RT mix to cells in EB (40uL RT mix + 10uL sample per reaction).
4. Split RT mix into PCR strips with 50µL/tube.
5. Set up thermocycler as below:

<table>
<tr><th>Temp</th><th>Time</th><th></th></tr>
<tr><td>50˚C</td><td>Hold</td><td></td></tr>
<tr><td>50˚C</td><td>10 min</td><td></td></tr>
<tr><td>8˚C</td><td>12s</td><td rowspan="6">3 cycles</td></tr>
<tr><td>15˚C</td><td>45s</td></tr>
<tr><td>20˚C</td><td>45s</td></tr>
<tr><td>30˚C</td><td>30s</td></tr>
<tr><td>42˚C</td><td>2 min</td></tr>
<tr><td>50˚C</td><td>3 min</td></tr>
<tr><td>50˚C</td><td>5 min</td><td></td></tr>
<tr><td>Total</td><td>37 min</td><td></td></tr>
</table>

6. All steps below are at room temperature.
7. Thaw barcode oligos plates, block 1, 2, 3 oligos and T4 DNA ligase buffer at room temp. Place NIB-RI at room temp. Set centrifuge to room temp.
8. Add 300µL NIB-RI to a new 2ml lobind tube per sample and pool RT reactions into it, pipette mix well.
9. Spin down at 500xg for 5 min at room temp in swing bucket centrifuge.
10. Wash with 500µL NIB-RI **without** disturbing the pellet.
11. Spin down at 500xg for 5 min at room temp in swing bucket centrifuge.
12. Remove supernatant and resuspend pellet ***thoroughly*** with appropriate volumes of NIB-RI. The total volume for a single 96 well plate of hybridization is 1080µL NIB-RI. Divide by the number of independent samples.
    a. Count nuclei to confirm nuclei quality and cell count (pre-splitpool)

Hybridization-ligation (~5hrs)

1. Make sure that all oligo plates are thawed to room temp and spun down before hybridization.
2. Prepare hybridization buffer in 5mL tube:

Hybridization buffer	Vol for 96 wells (µL)
Ultrapure water	2589.3
T4 ligase buffer (10x)	540
SUPERase RI	13.5
Enzymatics RI	43.2
10% NP-40	54
Total	3240

3. Add appropriate volumes of hybridization buffer to resuspended samples in NIB-RI, mix well. If there are multiple samples, then divide the hybridization buffer appropriately for number of sample.
4. Using a multichannel trough, aliquot 40µL sample mixture to Round 1 plate, mix, and seal plate.
5. Shake at 300rpm for 30min at 25°C.

6. Prepare blocking oligo 1 mixture:

Blocking oligo 1	Vol (µL)
Round 1 blocking (1mM)	25.3
T4 ligase buffer (10x)	211.2
Ultrapure water	915.5
Total	1152

   a. Distribute 90µL each to a PCR strip (12x) and use a P20 multichannel to distribute blocking oligo and mix.
7. Using a whole new P10 pipette tip box, add 10µL blocking oligo 1 mixture to each well, mix, and seal plate.
8. Shake at 300 rpm for 30 min at 25°C.

9. Pool samples into a trough, and using a whole new P200 pipette tip box, aliquot 50µL of mixture to Round 2 plate.
   a. Use a P200 multichannel to aspirate and pool into a new basin and redistribute using a multichannel.
10. Mix, seal plate, and shake at 300rpm for 30min at 25°C.

11. Prepare blocking oligo 2 mixture:

Blocking oligo 2	Vol (µL)
Round 2 blocking (1mM)	30.4
T4 ligase buffer (10x)	211.2
Ultrapure water	910.4
Total	1152

   a. Distribute 90µL each to a PCR strip (12x) and use a P20 multichannel to distribute blocking oligo and mix.
12. Using a whole new P10 pipette tip box, add 10µL blocking oligo 2 mixture to each well, mix, and seal plate.
13. Shake at 300rpm for 30min at 25°C.

14. Pool samples into a trough, using a whole new P200 pipette tip box, aliquot 60µL of mixture to Round 3 plate.
   a. Use a P200 multichannel to aspirate and pool into a new basin and redistribute using a multichannel.
15. Mix, seal plate, and shake at 300rpm for 30min at 25°C.

16. Prepare blocking oligo 3 mixture:

Blocking oligo 3	Vol (µL)
Round 3 blocking (1mM)	26.5
10% NP-40 (can be replaced with water)	11.5
Ultrapure water	1114
Total	1152

   b. Distribute 90µL each to a PCR strip (12x) and use a P20 multichannel to distribute blocking oligo and mix.
17. Using a whole new P10 pipette tip box, add 10µL blocking oligo 3 mixture to each well. mix, and seal plate.
18. Shake at 300rpm for 30min at 25°C.
19. Pool samples to a trough and add 70µL 7.5% BSA to the solution and mix well.
   c. Can filter while transferring using a FlowMi 40µm filter to remove multiplets.

20. Transfer to 4x lo-bind 2mL tubes, spin down at 500xg for 5 min at room temp in a swing bucket centrifuge.
21. Remove supernatant and add ***gently*** 1mL NIB-RI to pellet **without** resuspending.

22. Spin down at 500xg for 5 min at room temp, remove supernatant.
23. Resuspend with 85µL NIB-RI: start with 40µL, pool all tubes, measure volume and top up to 85 µL.
24. Prepare Ligation mix:

**Ligation buffer**	**Vol (µL)**
Ultrapure water	251.8
T4 ligase buffer (10x)	40
SUPERase RI	1
Enzymatics RI	3.2
10% NP-40	4
T4 ligase	20
Total	320

25. Mix 80µL sample to 320µL ligation mix.
26. Distribute 50µL to 8 PCR tubes.
27. Shake at 300rpm for 30 min at 25°C.

28. Pool samples, spin down at 500xg for 5 min at room temp.
29. Remove supernatant and wash pellet with 1mL NIB-RI.
30. Spin down at 500xg for 5 min at room temp.
31. Resuspend ***thoroughly*** (10x pipetting) with **200µL NIB-RI and count nuclei (post-ligation).
    d. Volume of final resuspension depends on expected nuclei recovery. Generally, can assume between 100-200k but will change depending on cell type and the number of cells at the start of split and pool.
    e. **400µL assumes 200k final recovery. Adjust appropriately for desired number of cells per library.

<u>Reverse crosslinking & pull down (2:45 hrs)</u>

1. Based on desired sub-library size (# cells), dilute the cells appropriately for each 50µL reaction.
    a. Range: 2,000-20,000 cells / 50µL.
    b. Each sub-library is processed independently.
    c. N = number of sublibraries
2. Prepare reverse crosslinking master mix as follows.

**RC MM**	**Vol for 1 sublib (µL)**
2x RCB Buffer	50
proteinase K (20mg/mL)	2
SUPERase RI	1
Total	53

3. For each tube of 50µL sample, add 53 µL of RC MM.

4. Incubate at 55˚C for 1h.
    a. Do not incubate longer, decreases yield

5. Recommended stopping point, store product at -80˚C for few weeks

6. Thaw libraries, if necessary, PMSF/IPA, equilibrate B&W buffers to room temp.
7. After thaw, stop proteinase K by adding 5µL 100mM PMSF/IPA (don't freeze thaw) and incubate at room temp for 10 min.
8. Prepare following buffers (N = number of sublibraries):

**1x B&W-T/RI**	**Vol for 1 sublib (µL)**
1x B&W-T	400
SUPERase RI	4
Total	404

2x B&W/RI	Vol for 1 sublib (µL)
2x BW	100
SUPERase RI	2
Total	102

1x STE/RI	Vol for 1 sublib (µL)
STE	200
SUPERase RI	1
Total	201

9. For n number of sublibraries, mix 11*n µL Streptavidin Dynabeads with 110*n µL 1x B&W-T and place it on a magnetic rack
10. Remove supernatant, wash ***twice*** with 110*n µL 1x B&W-T (no RNase inhibitors).
11. Remove supernatant, wash ***once*** with 110*n µL 1x B&W-T/RI (w/ RNase inhibitors).
12. Resuspend beads in 110*n µL **2x** B&W/RI.
13. Add 100µL beads to each sample and rotate over head at 10 rpm for 60 min at room temp. Ensure lids are closed.
    a. Prepare tubes for ATAC supernatant and remember that you need the SUPERNATANT.
    b. Thaw ATAC PCR reagents, thaw reagents + make TSO mix (need it immediately in Template Switching step).
14. Put on a magnetic rack, transfer supernatant containing chromatin fragments to a new tube for library preparation.
    a. Supernatant = ATAC (stable for few hours at room temp, ideally on ice, also can be processed in parallel)
        i. **DO NOT THROWAWAY SUPERNATANT**
        ii. **Refer to ATAC library preparation section for next steps with supernatant**
    b. Beads = RNA
15. Wash beads with 100µL B&W-T/RI ***three times***.
16. Put sample on the magnetic rack, add 100µL 1x STE/RI **without resuspending beads**.

RNA library preparation – template switching (2.5hrs)

1. Prepare template switch mix, use wide bore tips for Ficoll and 50% PEG:

Template switch mix	Vol for 1 sublib (µL)
Ultrapure water	1.25
5x RT buffer	10
50% PEG 6000	15
Ficoll PM-400 (20%)	10
10mM dNTPs (each)	5
NxGen RNAse inhibitor	5
TSO 100µM	1.25
Maxima H Minus RT (add right before rxn)	2.5
Total	50

2. Remove all supernatant (100µL 1x STE/RI) and resuspend beads in 50µL template switch mix
    a. Avoid letting beads dry. Do so by first quickly adding the template switch mix to each sample to coat the bead before going back and resuspending all samples
    b. Ensure the beads are thoroughly resuspended. Can be helpful to start with a multichannel at first, and then also pipette each sample individually. Bubbles are inevitable at this stage and during TSO, so the main focus is to get everything resuspended.
3. Rotate samples at room temp for 30 min at 10 rpm.
4. Set thermomixer to 42˚C before step 3 ends. Incubate samples at 42˚C for 90 min shaking at 700rpm.
    a. Resuspend beads every 30 min by pipetting up and down.

RNA library preparation – on bead cDNA amplification/clean up (2.5hrs)

1. Prepare cDNA amplification PCR mastermix

**cDNA PCR mastermix**	**Vol for 1 sublib (µL)**
Ultrapure water	25.74
KAPA HiFi 2x MM	27.5
RNA PCR primer 25µM	0.88
P7 primer 25µM	0.88
Total	55

2. Add 100µL of STE to each sample and mix to make viscous solution more pipettable.
3. Place on magnet and discard supernatant.
4. Wash bound beads with 200µL STE (**do not resuspend and do not disturb pellet**) and let sit for a bit.
5. Remove STE and add 55µL PCR mix to beads.
6. Set up thermocycler as below:

**Temp**	**Time**	
95˚C	3 min	
98˚C	20s	5 cycles
65˚C	45s	
72˚C	3 min	
4˚C	Hold	
Total	30 min	

7. For each sublibrary, check by qPCR for additional cycles as follows:
    a. Prepare qPCR mastermix:

**qPCR mastermix for RNA**	**Vol for 1 sublib (µL)**
KAPA HIFI 2x MM	3.75
RNA PCR primer 25µM	0.12
P7 primer 25µM	0.12
EVAgreen 20x	0.5
Ultrapure water	3.01
Total	7.5

    b. Put samples on magnet, mix 2.5µL sample supernatant with 7.5µL qPCR mix.
    c. Run thermocycler as below for qPCR:

**Temp**	**Time**	
95˚C	3 min	
98˚C	20s	20 cycles for qPCR / 0.33Ct for add PCR
65˚C	20s	
72˚C	3 min	
4˚C	Hold	

8. Perform additional cycles based on 0.33Ct of maximum saturation.
    a. Expect 6-7 addl cycles for 2k cells, ~4 for 10k cells.
9. Magnetically separate out the Dynabeads, purify the supernatant of each sample with 40µL SPRI beads (0.8x) in new tubes, making sure no Dynabeads come through.
    a. Make fresh 80% EtOH (400µL per sublib).
    b. Add 40µL SPRI beads to each 50µL reaction.
    c. Incubate for 5min at room temp.
    d. Put on magnet and remove supernatant.
    e. Wash with 200µL 80% EtOH directly on magnet, waiting 30s each. Repeat for a total of two washes.
    f. Carefully remove all supernatant and air dry the beads until they just turn matte.
    g. Remove from magnet, thoroughly resuspend in 15µL EB to elute. Let sit for 2min before briefly centrifuging and putting back on magnet. Transfer supernatant to a new tube.
10. cDNA can be stored at -20˚C indefinitely until tagmentation.

RNA library preparation – cDNA Tagmentation

1. Quantify cDNA concentration by Qubit using 1µL cDNA.
    a. If necessary, dilute 50ng cDNA to 5 ng/µL in water.
    b. Can get away with 20ng only if necessary.
2. Start thermocyler protocol to ensure the machine is at 55˚C hold before samples go in.
3. Mix following together:

cDNA tagmentation	Vol for 1 sublib (µL)
2x TD buffer	25
SHARE-cDNA Tn5 WJG Mar 2023	1
Subtotal	26
cDNA (5ng/µL)	10
Ultrapure water	14
Total	50

4. Incubate at 55˚C on thermocycler for 5 min.
5. Purify library with Zymo DNA Clean and Concentrate (250µL binding buffer). Try to get the samples to binding buffer asap to minimize over tagmentation.
    a. Elute twice with 11µL EB each time (22µL total).
6. Prepare post-tagmentation PCR mix, record the i5 index used for each sublib.

Post tagmentation PCR mix	Vol for 1 sublib (µL)
NEBnext 2x MM	25
P7 primer 25µM	1
Ultrapure water	3
Subtotal	29
Eluted sample	20
i5 index primer (Ad 1.xx) 25µM	1
Total	50

7. Set up thermocycler for the sample as below:

Temp	Time	
72˚C	5 min	
98˚C	30s	
98˚C	10s	10 cycles (11 cycles if cDNA input is ~20ng)
65˚C	30s	
72˚C	60s	
4˚C	Hold	
Total	20 min	

8. Purify each sample with 40µL SPRI beads (0.8x) and elute library to 15µL EB.
    a. If there is a significant amount of large fragments present in tapestation after this step, can redo purification with a double sided SPRI (0.6x-0.8x) or gel extraction.
9. Libraries can be stored at -20˚C indefinitely.

ATAC library preparation

1. Clean the supernatant with Zymo DNA clean and concentrate kit by adding 5x DNA binding buffer (200µL rxn = 1mL of binding buffer, spin twice because the column only holds 0.7mL).
   a. Elute in 11µL EB then add 11µL EB again for a total elution volume of 22µL.
2. Prepare ATAC PCR mix, record the i5 index used for each sublib

ATAC PCR mastermix	Vol for 1 sublib (µL)
NEBnext 2x MM	25
P7 primer 25µM	1
Ultrapure water	3
Subtotal	29
Eluted sample	20
i5 index primer (Ad 1.xx) 25µM	1
Total	50

3. Mix and set up thermocycler as shown below:

Temp	Time	
72˚C	5 min	
98˚C	30s	
98˚C	10s	5 cycles
65˚C	30s	
72˚C	30s	
4˚C	Hold	
Total	15 min	

4. Check by qPCR as usual for ATAC:
   a. 5µL sample + 10µL qPCR mix:

qPCR mastermix for ATAC	Vol for 1 sublib (µL)
NEBnext 2x MM	5
Ad 1.1 25µM	0.2
P7 primer 25µM	0.2
10x SYBRgreen	0.9
Ultrapure water	3.7
Total	10

      i. Dilute 100x SYBR to 10x before use fresh

   b. Run qPCR as below

Temp	Time	
98˚C	30s	
98˚C	10s	20 cycles for qPCR / 0.33Ct for addl PCR
65˚C	30s	
72˚C	30s	
4˚C	Hold	

   c. Perform additional cycles based on 1/3 of the plateau of maximum fluorescence from qPCR (0.33Ct) (usually ~8 additional cycles for 2k cells).
5. Clean up with Zymo DNA clean and concentrate (225µL binding buffer if qPCR) and elute twice 8µL each for ~15µL total.
6. Libraries can be stored at -20˚C indefinitely.

Library quantification and sequencing

1. Measure concentration of libraries using Qubit
2. Run ATAC library on D5000 tapestation (10μL buffer + 1μL sample). Expected ATAC tapestation trace:

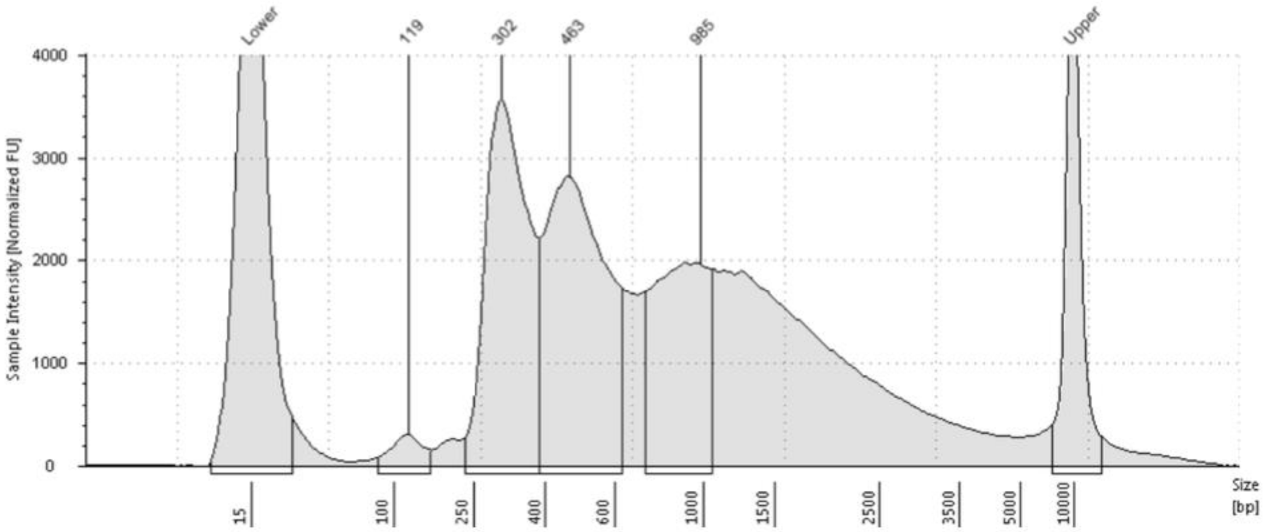

3. Run RNA library on D1000 tapestation (3μL buffer + 1μL sample). Expected RNA tapestation trace:

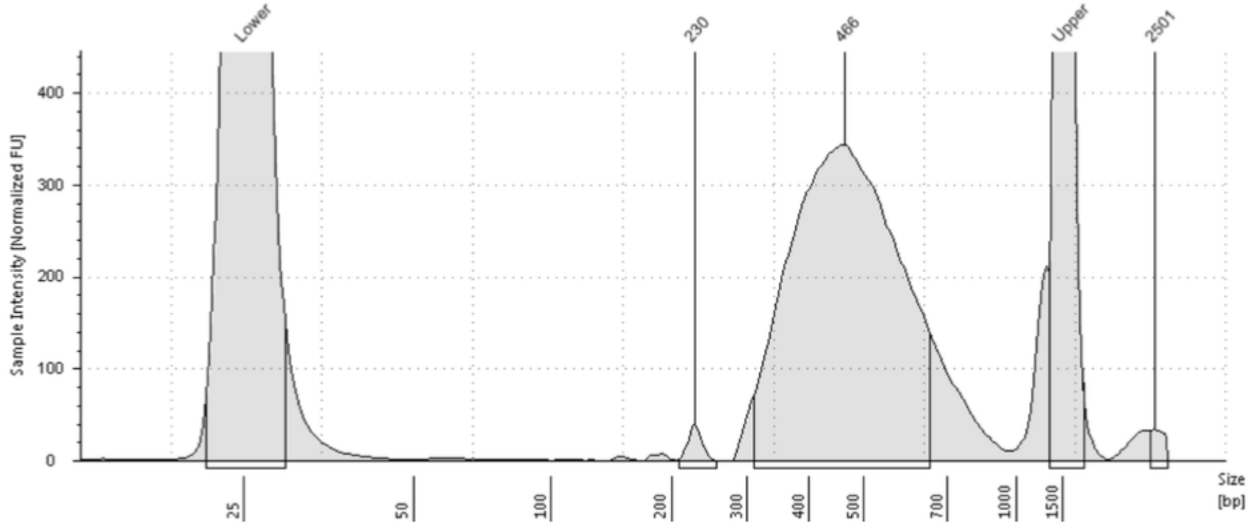

4. Sequencing configuration

If sequencing ATAC only or ATAC+RNA together:

# cycles in seq kit	R1	I1	I2	R2
150 cycle	30	99	8	30
300 cycle	96	99	8	96

If sequencing RNA only:

# cycles in seq kit	R1	I1	I2	R2
150 cycle	50	99	8	10
300 cycle	162	99	8	30

The general sequencing depth recommendation is 50,000 reads per cell per modality (ATAC or RNA). For example, for a cell sublibrary of 20,000 cells, use 2 billion reads for both ATAC and RNA sublibraries.

Materials and equipment

*The volumes shown in the table below does not include amount needed for pre-made buffers

category	material	vendor	catalog #	vol needed for this exp (µL)	storage location	Notes (including vol per sample/rxn in µL)
Enzymes	SHARE-ATAC Tn5	In house	WJG Mar 2023	312.5	-20C	2.5/ATAC rxn
	SHARE-cDNA Tn5	In house	WJG Mar 2023	17.6	-20C	1/sublib
	Maxima H Minus RT and buffer	Thermo	EP0753	319	-20C	5/RT rxn+2.5/sublib
	T4 DNA Ligase	NEB	M0202L	20	-20C	20 fixed
	20mg/mL Proteinase K	NEB	P8107S	35.2	-20C	2/sublib
	Kapa Hifi Hotstart Ready Mix	Roche	7958935001	550	-20C	31.25/sublib
	NEBNext 2x MM	NEB	M0541L	968	-20C	55/sublib
RNase inhibitors	Enzymatics RI	Enzymatics	Y9240L	314.1758	-20C	17.52/sample + 0.8625/ ATAC rxn + 0.31/RT rxn + 62.9 fixed
	Superase RI	Thermo	AM2696	261.01	-20C	8.27/sample + 0.63/RT rxn + 8/sublib + 30.98 fixed
	NxGen RNAse inhibitor	Lucigen	30281-1	88	-20C	5/sublib
Beads	Dynabeads MyOne Streptavidin C1	Thermo	65002	176	4C	10/sublib
	SPRI beads	Beckman	B23318	1408	25C	80/sublib
Chemicals	7.5% BSA	Thermo	15260037	1275.36	4C	182.63/sample, 70 fixed
	10% Tween-20	Biorad	1662404	141.4	25C	11/sample, 0.5/ATAC rxn
	10% NP-40	Thermo	85124	76.1	25C	1/sample, 69.5 fixed
	1% Digitonin	Promega	G9441	75.4	-20C	Dilute to 1%, 1/sample +0.5/ATAC rxn
	1M DTT	Sigma	646563	7.3	-20C	1.1/sample
	16% Formaldehyde (FA)	Thermo	28906	66	25C	10/sample
	100% DMF	Thermo	20673	1100	25C	8/ATAC rxn
	PIC (protease inhibitor cocktail)	Sigma	P8340	27.5	-20C	0.2/ATAC rxn
	dNTPs (10mM each)	NEB	N0447L	225.5	-20C	2.5/RT rxn, 5/sublib
	50% PEG 6000			1089	25C	15/RT rxn, 15/sublib
	Buffer EB	Qiagen	19086	lots	25C	
	10x T4 DNA Ligase Buffer	NEB	B0202S	1002.4	-20C	1002.4 fixed
	PMSF 100mM	Thermo	36978	88	-20C	5/sublib
	Ficoll PM-400 (20%)	Sigma	F5415	176	4C	10/sublib
	10x SYBRgreen	Thermo	S7563	15.8	-20C	Dilute stock to 10x, 0.9/sublib
	20x EVAgreen	Biotium	31000	8.8	-20C	0.5/sublib
	5M NaCl	Any			25C	
	1M MgCl2				25C	
	1M Tris HCl pH 7.5				25C	
	1M Tris HCl pH 8.0				25C	
	2.5M Glycine				25C	
	0.2M Tris-acetate				4C	
	5M K-acetate				25C	
	1M Mg-acetate				25C	
	1x PBS				25C	
	20% SDS				25C	
	0.5M EDTA				25C	
Oligos	RT primer 100µM	See Sai Ma et al Cell 2020 Supp Table for all oligo sequences		275	-20C	5/RT rxn
	RNA PCR Primer 25µM			17.6	-20C	1/sublib
	P7 primer 25µM			56.3	-20C	3.2/sublib
	Nextera i5 index primers Ad 1.xx 25µM			32 x 1	-20C	1/atac or rna sublib, all unique
	TSO 100µM			22	-20C	1.25/sublib
	Round 1 blocking 1mM			25.3	-20C	25.3 fixed
	Round 2 blocking 1mM			30.4	-20C	30.4 fixed
	Round 3 blocking 1mM			26.5	-20C	26.5 fixed
Kits	DNA clean and concentrate kit	Zymo	D4014	48 assays	25C	
	Qubit dsDNA HS assay	Thermo	Q32854	32 assays	25C /4C	
	D5000 ScreenTape	Agilent	5067-5588	17 assays	4C	
	D5000 Reagents	Agilent	5067-5589	17 assays	4C	
	D1000 ScreenTape	Agilent	5067-5582	17 assays	4C	
	D1000 Reagents	Agilent	5067-5583	17 assays	4C	
Equipment	Centrifuge	Any				
	FlowMi 40um filter					
	Wide bore p200 and p1000 tips					
	Tube rotator					

Supplementary Note 2: Quality control per organ.

Quality control for Adrenal (AG), Brain (BR), Eye (EY), Heart (HT), Liver (LI), Lung (LU), Muscle (MU), Skin (SK), Spleen (SP), StomachEsophagus (ST), Thymus (TM), and Thyroid (TR). a) UMAP of organ and gestational age per cluster in post-conception weeks. **b)** RNA marker gene dot plot for each cell cluster. **c)** RNA QC metrics (# UMIs, # Genes) and ATAC QC metrics (# Fragments, TSS enrichment ratio) for each cluster. **d)** ATAC marker peaks (left) and motif enrichment within marker peaks (right), columns sorted by hierarchical clustering of motif enrichments. **e)** Top TF motifs with the highest variability across clusters from chromVAR deviation calculations. **f)** Normalized (RPKM) accessibility tracks centered at housekeeping gene GAPDH.

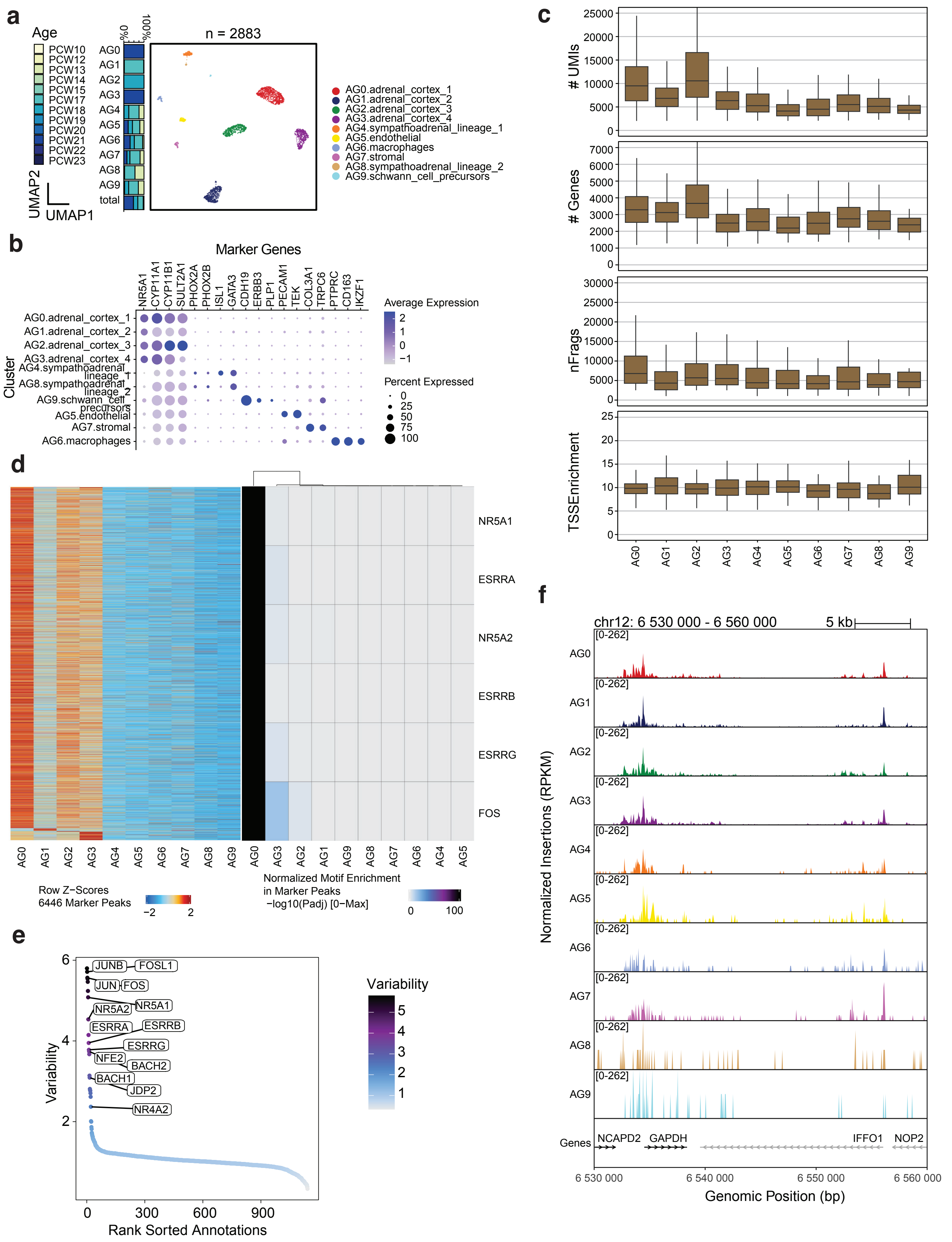
a
Age
PCW10
PCW12
PCW13
PCW14
PCW15
PCW17
PCW18
PCW19
PCW20
PCW21
PCW22
PCW23
UMAP2
UMAP1
0%
100%
n = 2883
AG0.adrenal_cortex_1
AG1.adrenal_cortex_2
AG2.adrenal_cortex_3
AG3.adrenal_cortex_4
AG4.sympathoadrenal_lineage_1
AG5.endothelial
AG6.macrophages
AG7.stromal
AG8.sympathoadrenal_lineage_2
AG9.schwann_cell_precursors
b
Marker Genes
NR5A1
CYP11A1
CYP11B1
SULT2A1
PHOX2A
PHOX2B
ISL1
GATA3
CDH19
ERBB3
PLP1
PECAM1
TEK
COL3A1
TRPC6
PTPRC
CD163
IKZF1
Cluster
Average Expression
Percent Expressed
c
UMIs
Genes
nFrags
TSSEnrichment
d
NR5A1
ESRRA
NR5A2
ESRRB
ESRRG
FOS
Row Z-Scores
6446 Marker Peaks
Normalized Motif Enrichment
in Marker Peaks
−log10(Padj) [0−Max]
e
JUNB
FOSL1
JUN
FOS
NR5A2
NR5A1
ESRRA
ESRRB
ESRRG
NFE2
BACH2
BACH1
JDP2
NR4A2
Variability
Rank Sorted Annotations
f
chr12: 6 530 000 - 6 560 000
5 kb
[0-262]
Normalized Insertions (RPKM)
Genes
NCAPD2
GAPDH
IFFO1
NOP2
Genomic Position (bp)

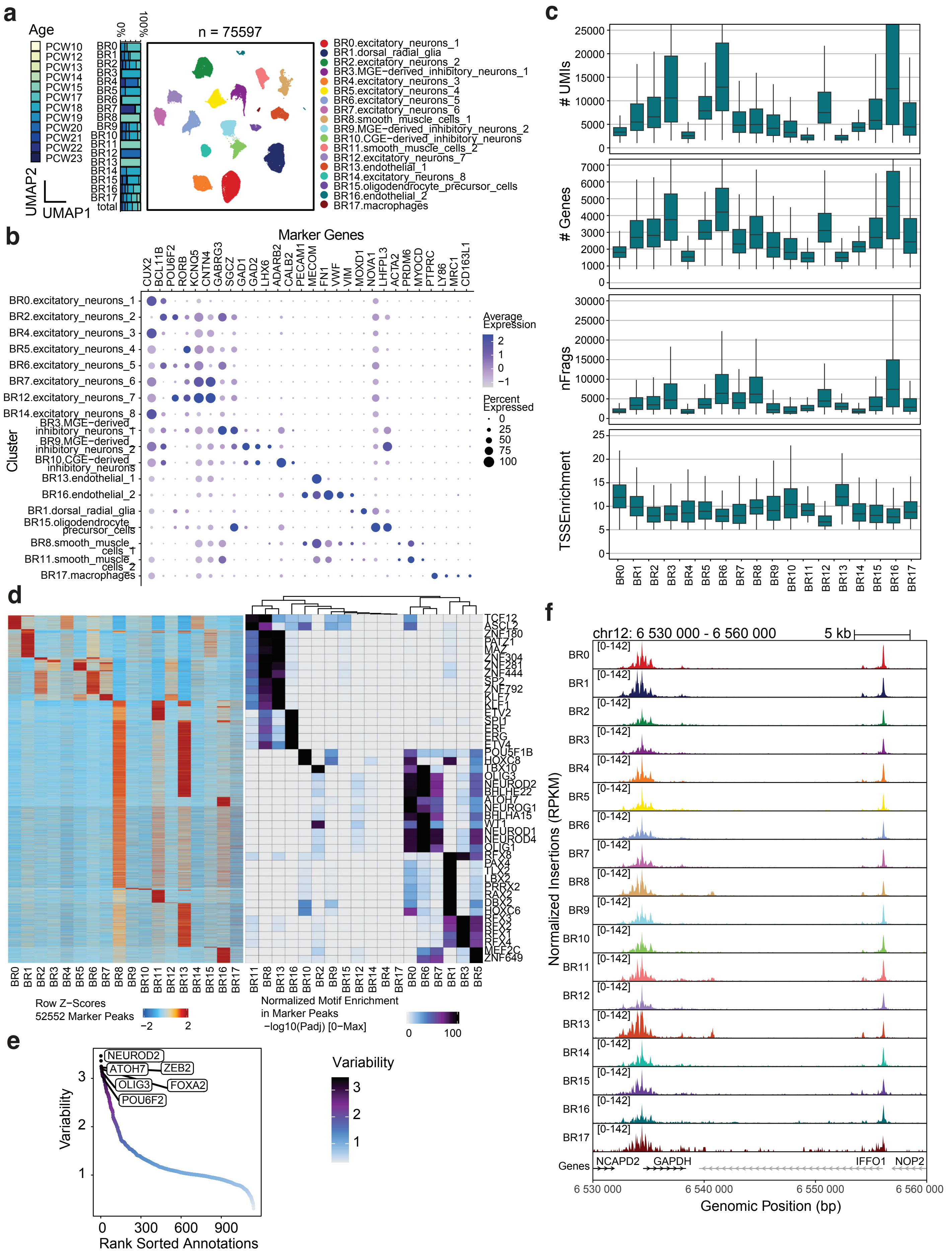
a
Age
PCW10
PCW12
PCW13
PCW14
PCW15
PCW17
PCW18
PCW19
PCW20
PCW21
PCW22
PCW23
UMAP2
UMAP1
n = 75597
BR0.excitatory_neurons_1
BR1.dorsal_radial_glia
BR2.excitatory_neurons_2
BR3.MGE−derived_inhibitory_neurons_1
BR4.excitatory_neurons_3
BR5.excitatory_neurons_4
BR6.excitatory_neurons_5
BR7.excitatory_neurons_6
BR8.smooth_muscle_cells_1
BR9.MGE−derived_inhibitory_neurons_2
BR10.CGE−derived_inhibitory_neurons
BR11.smooth_muscle_cells_2
BR12.excitatory_neurons_7
BR13.endothelial_1
BR14.excitatory_neurons_8
BR15.oligodendrocyte_precursor_cells
BR16.endothelial_2
BR17.macrophages
b
Marker Genes
Cluster
Average Expression
Percent Expressed
c
UMIs
Genes
nFrags
TSSEnrichment
d
Row Z−Scores
52552 Marker Peaks
Normalized Motif Enrichment in Marker Peaks −log10(Padj) [0−Max]
e
Variability
Rank Sorted Annotations
NEUROD2
ATOH7
ZEB2
OLIG3
FOXA2
POU6F2
f
chr12: 6 530 000 - 6 560 000
5 kb
Normalized Insertions (RPKM)
Genes
NCAPD2
GAPDH
IFFO1
NOP2
Genomic Position (bp)

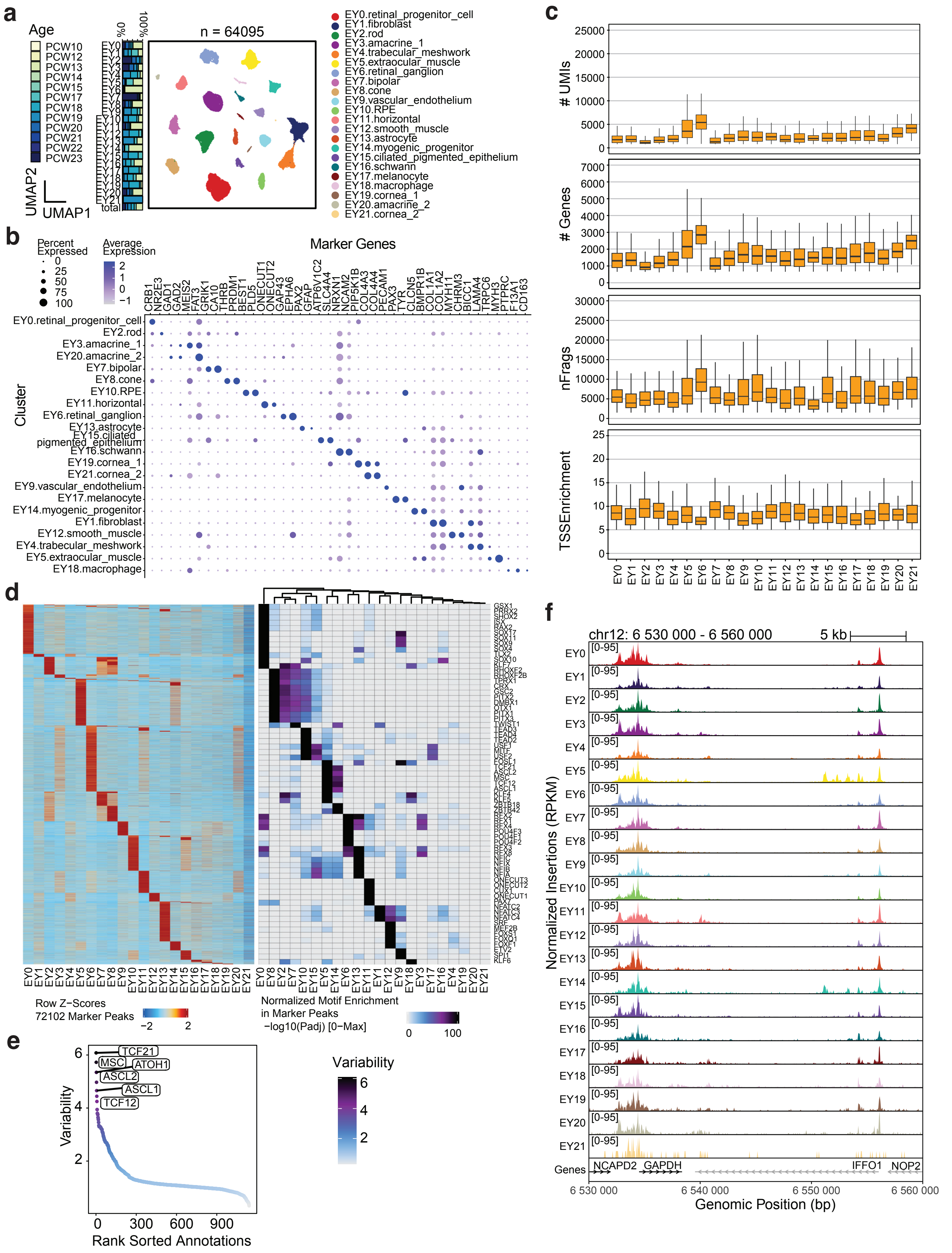
a
Age
PCW10
PCW12
PCW13
PCW14
PCW15
PCW17
PCW18
PCW19
PCW20
PCW21
PCW22
PCW23
0%
100%
total
n = 64095
UMAP2
UMAP1
EY0.retinal_progenitor_cell
EY1.fibroblast
EY2.rod
EY3.amacrine_1
EY4.trabecular_meshwork
EY5.extraocular_muscle
EY6.retinal_ganglion
EY7.bipolar
EY8.cone
EY9.vascular_endothelium
EY10.RPE
EY11.horizontal
EY12.smooth_muscle
EY13.astrocyte
EY14.myogenic_progenitor
EY15.ciliated_pigmented_epithelium
EY16.schwann
EY17.melanocyte
EY18.macrophage
EY19.cornea_1
EY20.amacrine_2
EY21.cornea_2
b
Percent Expressed
Average Expression
Marker Genes
Cluster
CRB1 NR2E3 GAD1 GAD2 MEIS2 FAT3 GRIK1 CA10 THRB PRDM1 BEST1 PLD5 ONECUT1 ONECUT2 GAP43 EPHA6 PAX2 GFAP ATP6V1C2 SLC4A4 NRXN1 NCAM2 PIP5K1B COL4A3 COL4A4 PECAM1 PAX3 TYR CLCN5 BMPR1B COL1A1 COL1A2 MYH11 CHRM3 BICC1 LAMA4 TRPC6 MYH3 PTPRC F13A1 CD163
c
UMIs
Genes
nFrags
TSSEnrichment
d
Row Z−Scores
72102 Marker Peaks
Normalized Motif Enrichment in Marker Peaks
−log10(Padj) [0−Max]
GSX1 CRX SHOX2 ISX RAX2 SOX17 SOX11 SOX9 SOX4 TLX2 SOX10 KLF1 RHOXF2 RHOXF2B TPRX1 CRX GSC2 PITX2 DMBX1 OTX1 PITX1 PITX3 TWIST1 TEAD3 TEAD4 TEAD2 USF1 MITF USF2 FOSL1 TCF21 ASCL2 MSC TCF12 ASCL1 KLF4 KLF5 ZBTB18 ZBTB42 RFX2 RFX1 RFX4 POU4F3 POU4F1 POU4F2 RFX3 RFX8 NFIC NFIX NFIB NFIA ONECUT3 ONECUT2 CUX1 ONECUT1 PAX7 NFATC2 NFATC3 NFATC4 SRF MEF2B FOXS1 FOXO1 FOXF1 ETV2 SPI1 KLF6
e
Variability
Rank Sorted Annotations
TCF21
MSC
ATOH1
ASCL2
ASCL1
TCF12
f
chr12: 6 530 000 - 6 560 000
5 kb
[0-95]
Normalized Insertions (RPKM)
Genes
NCAPD2
GAPDH
IFFO1
NOP2
Genomic Position (bp)

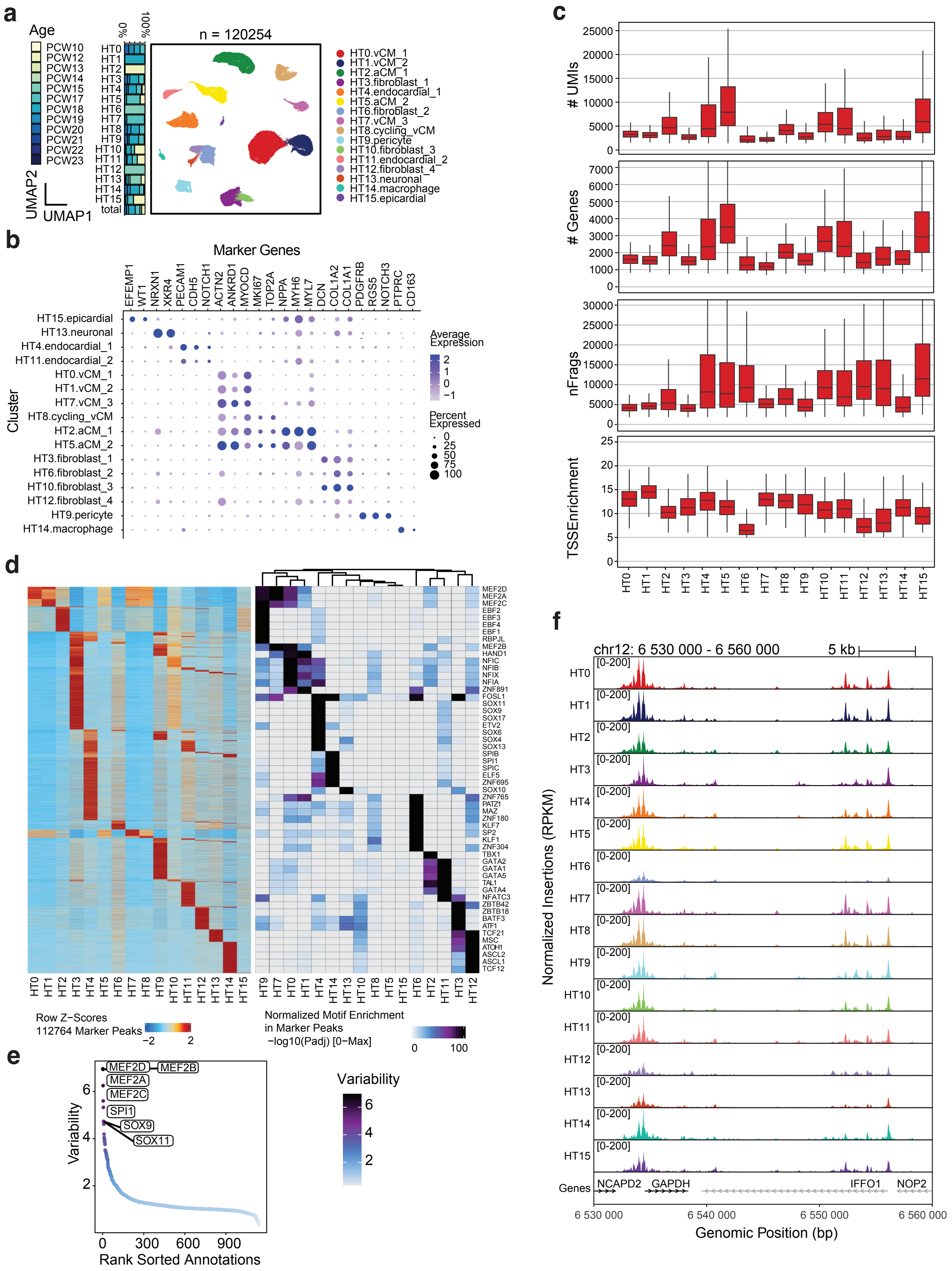
a
Age
PCW10
PCW12
PCW13
PCW14
PCW15
PCW17
PCW18
PCW19
PCW20
PCW21
PCW22
PCW23
0%
100%
HT0
HT1
HT2
HT3
HT4
HT5
HT6
HT7
HT8
HT9
HT10
HT11
HT12
HT13
HT14
HT15
total
n = 120254
UMAP2
UMAP1
HT0.vCM_1
HT1.vCM_2
HT2.aCM_1
HT3.fibroblast_1
HT4.endocardial_1
HT5.aCM_2
HT6.fibroblast_2
HT7.vCM_3
HT8.cycling_vCM
HT9.pericyte
HT10.fibroblast_3
HT11.endocardial_2
HT12.fibroblast_4
HT13.neuronal
HT14.macrophage
HT15.epicardial
b
Marker Genes
EFEMP1
WT1
NRXN1
XKR4
PECAM1
CDH5
NOTCH1
ACTN2
ANKRD1
MYOCD
MKI67
TOP2A
NPPA
MYH6
MYL7
DCN
COL1A2
COL1A1
PDGFRB
RGS5
NOTCH3
PTPRC
CD163
Cluster
HT15.epicardial
HT13.neuronal
HT4.endocardial_1
HT11.endocardial_2
HT0.vCM_1
HT1.vCM_2
HT7.vCM_3
HT8.cycling_vCM
HT2.aCM_1
HT5.aCM_2
HT3.fibroblast_1
HT6.fibroblast_2
HT10.fibroblast_3
HT12.fibroblast_4
HT9.pericyte
HT14.macrophage
Average Expression
2
1
0
−1
Percent Expressed
0
25
50
75
100
c
UMIs
25000
20000
15000
10000
5000
0
Genes
7000
6000
5000
4000
3000
2000
1000
0
nFrags
30000
25000
20000
15000
10000
5000
0
TSSEnrichment
25
20
15
10
5
0
HT0
HT1
HT2
HT3
HT4
HT5
HT6
HT7
HT8
HT9
HT10
HT11
HT12
HT13
HT14
HT15
d
MEF2D
MEF2A
MEF2C
EBF2
EBF3
EBF4
EBF1
RBPJL
MEF2B
HAND1
NFIC
NFIB
NFIX
NFIA
ZNF891
FOSL1
SOX11
SOX9
SOX17
ETV2
SOX6
SOX4
SOX13
SPIB
SPI1
SPIC
ELF5
ZNF695
SOX10
ZNF765
PATZ1
MAZ
ZNF180
KLF7
SP2
KLF1
ZNF304
TBX1
GATA2
GATA1
GATA5
TAL1
GATA4
NFATC3
ZBTB42
ZBTB18
BATF3
ATF1
TCF21
MSC
ATOH1
ASCL2
ASCL1
TCF12
HT0
HT1
HT2
HT3
HT4
HT5
HT6
HT7
HT8
HT9
HT10
HT11
HT12
HT13
HT14
HT15
HT9
HT7
HT0
HT1
HT4
HT14
HT13
HT10
HT8
HT5
HT15
HT6
HT2
HT11
HT3
HT12
Row Z−Scores
112764 Marker Peaks
−2
2
Normalized Motif Enrichment
in Marker Peaks
−log10(Padj) [0−Max]
0
100
e
Variability
MEF2D
MEF2B
MEF2A
MEF2C
SPI1
SOX9
SOX11
6
4
2
0
300
600
900
Rank Sorted Annotations
Variability
6
4
2
f
chr12: 6 530 000 - 6 560 000
5 kb
[0-200]
Normalized Insertions (RPKM)
HT0
HT1
HT2
HT3
HT4
HT5
HT6
HT7
HT8
HT9
HT10
HT11
HT12
HT13
HT14
HT15
Genes
NCAPD2
GAPDH
IFFO1
NOP2
6 530 000
6 540 000
6 550 000
6 560 000
Genomic Position (bp)

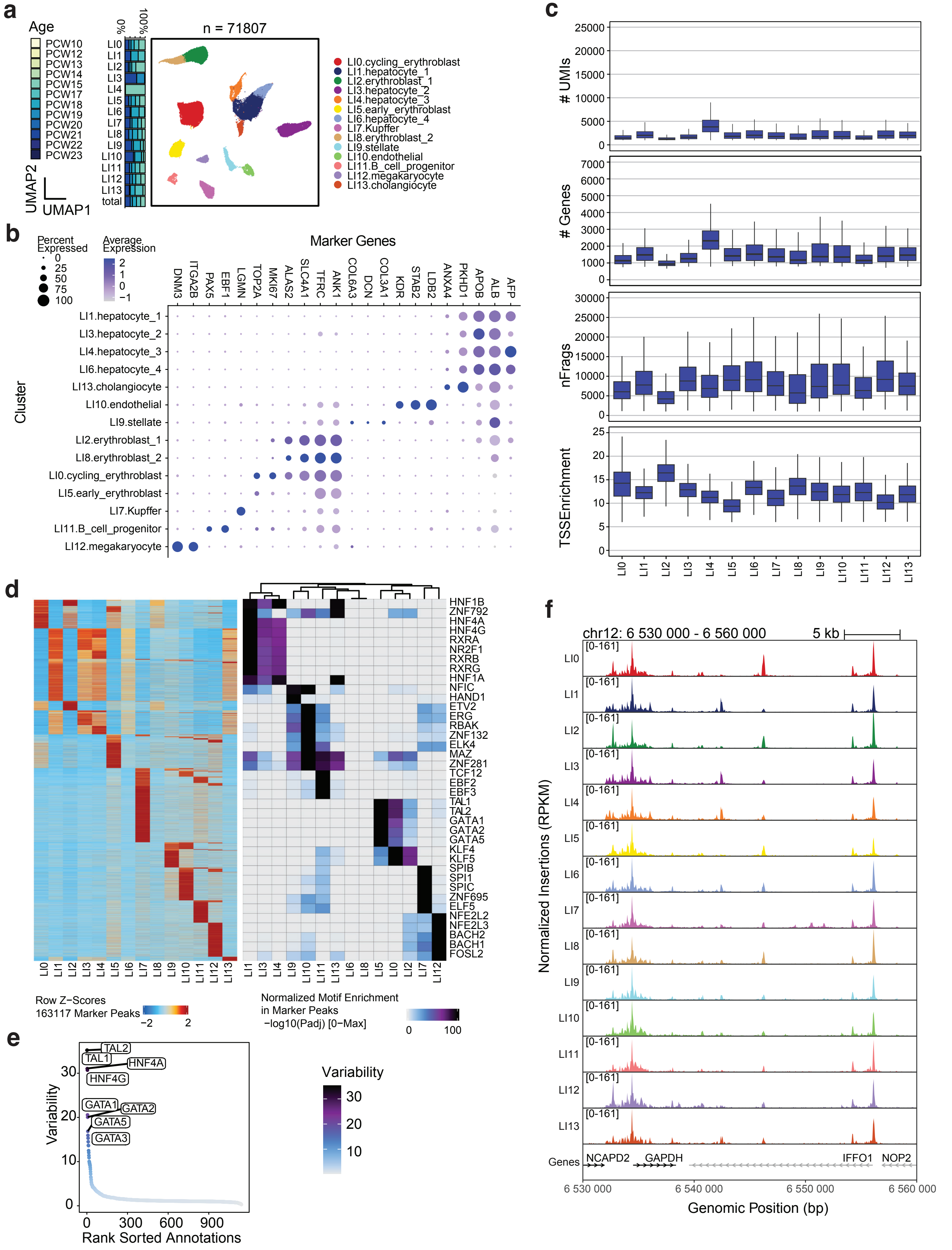
a
Age
PCW10
PCW12
PCW13
PCW14
PCW15
PCW17
PCW18
PCW19
PCW20
PCW21
PCW22
PCW23
UMAP2
UMAP1
0%
100%
LI0
LI1
LI2
LI3
LI4
LI5
LI6
LI7
LI8
LI9
LI10
LI11
LI12
LI13
total
n = 71807
LI0.cycling_erythroblast
LI1.hepatocyte_1
LI2.erythroblast_1
LI3.hepatocyte_2
LI4.hepatocyte_3
LI5.early_erythroblast
LI6.hepatocyte_4
LI7.Kupffer
LI8.erythroblast_2
LI9.stellate
LI10.endothelial
LI11.B_cell_progenitor
LI12.megakaryocyte
LI13.cholangiocyte
b
Percent Expressed
0
25
50
75
100
Average Expression
2
1
0
−1
Marker Genes
DNM3
ITGA2B
PAX5
EBF1
LGMN
TOP2A
MKI67
ALAS2
SLC4A1
TFRC
ANK1
COL6A3
DCN
COL3A1
KDR
STAB2
LDB2
ANXA4
PKHD1
APOB
ALB
AFP
Cluster
LI1.hepatocyte_1
LI3.hepatocyte_2
LI4.hepatocyte_3
LI6.hepatocyte_4
LI13.cholangiocyte
LI10.endothelial
LI9.stellate
LI2.erythroblast_1
LI8.erythroblast_2
LI0.cycling_erythroblast
LI5.early_erythroblast
LI7.Kupffer
LI11.B_cell_progenitor
LI12.megakaryocyte
c
UMIs
Genes
nFrags
TSSEnrichment
LI0
LI1
LI2
LI3
LI4
LI5
LI6
LI7
LI8
LI9
LI10
LI11
LI12
LI13
d
HNF1B
ZNF792
HNF4A
HNF4G
RXRA
NR2F1
RXRB
RXRG
HNF1A
NFIC
HAND1
ETV2
ERG
RBAK
ZNF132
ELK4
MAZ
ZNF281
TCF12
EBF2
EBF3
TAL1
TAL2
GATA1
GATA2
GATA5
KLF4
KLF5
SPIB
SPI1
SPIC
ZNF695
ELF5
NFE2L2
NFE2L3
BACH2
BACH1
FOSL2
Row Z−Scores
163117 Marker Peaks
−2
2
Normalized Motif Enrichment
in Marker Peaks
−log10(Padj) [0−Max]
0
100
e
TAL2
TAL1
HNF4A
HNF4G
GATA1
GATA2
GATA5
GATA3
Variability
Rank Sorted Annotations
f
chr12: 6 530 000 - 6 560 000
5 kb
[0-161]
Normalized Insertions (RPKM)
Genes
NCAPD2
GAPDH
IFFO1
NOP2
6 530 000
6 540 000
6 550 000
6 560 000
Genomic Position (bp)

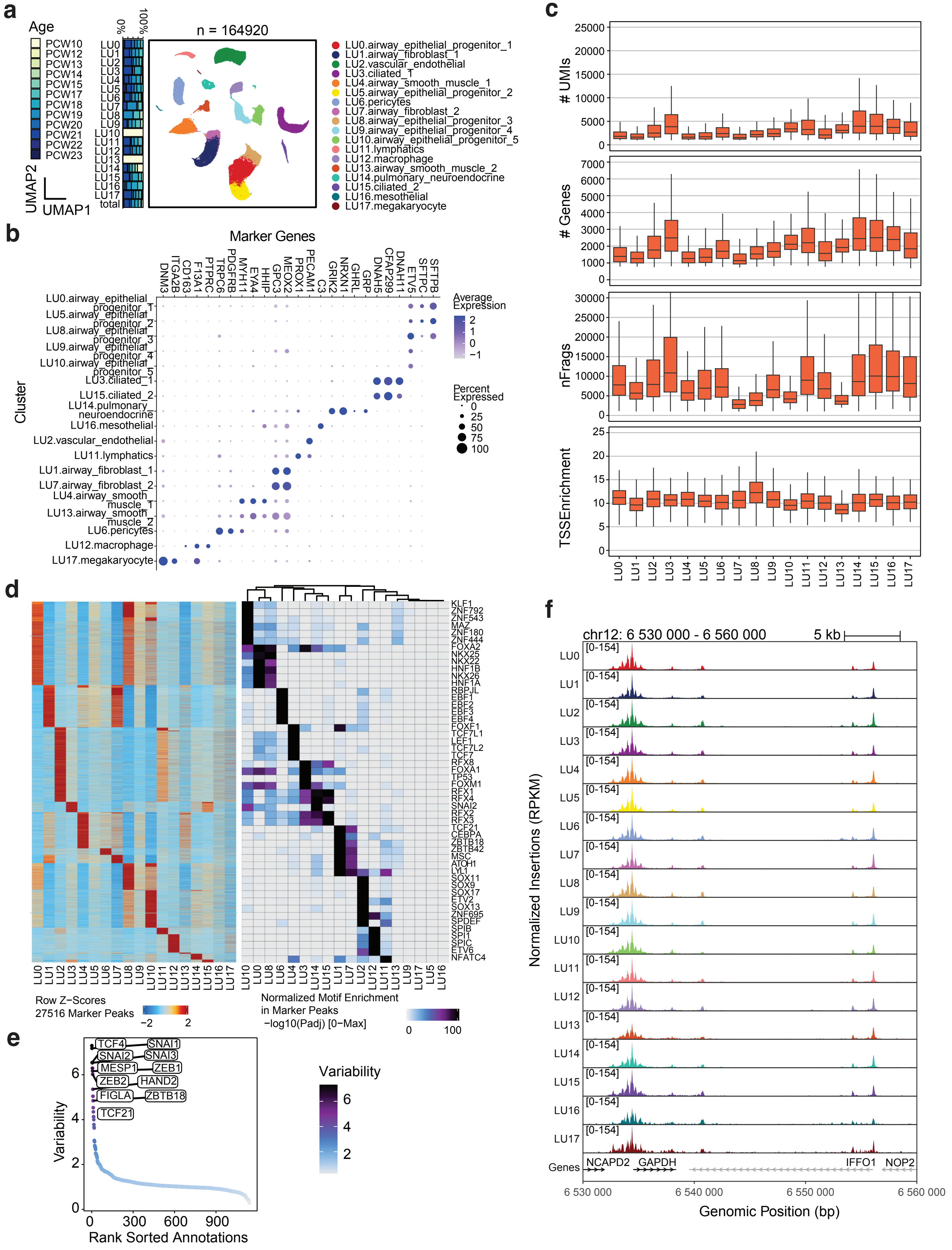
a
Age
PCW10
PCW12
PCW13
PCW14
PCW15
PCW17
PCW18
PCW19
PCW20
PCW21
PCW22
PCW23
0%
100%
UMAP2
UMAP1
n = 164920
LU0.airway_epithelial_progenitor_1
LU1.airway_fibroblast_1
LU2.vascular_endothelial
LU3.ciliated_1
LU4.airway_smooth_muscle_1
LU5.airway_epithelial_progenitor_2
LU6.pericytes
LU7.airway_fibroblast_2
LU8.airway_epithelial_progenitor_3
LU9.airway_epithelial_progenitor_4
LU10.airway_epithelial_progenitor_5
LU11.lymphatics
LU12.macrophage
LU13.airway_smooth_muscle_2
LU14.pulmonary_neuroendocrine
LU15.ciliated_2
LU16.mesothelial
LU17.megakaryocyte
total
b
Marker Genes
Cluster
Average Expression
Percent Expressed
c
UMIs
Genes
nFrags
TSSEnrichment
d
Row Z-Scores
27516 Marker Peaks
Normalized Motif Enrichment in Marker Peaks
−log10(Padj) [0−Max]
e
Variability
Rank Sorted Annotations
TCF4
SNAI1
SNAI2
SNAI3
MESP1
ZEB1
ZEB2
HAND2
FIGLA
ZBTB18
TCF21
f
chr12: 6 530 000 - 6 560 000
5 kb
[0-154]
Normalized Insertions (RPKM)
Genes
NCAPD2
GAPDH
IFFO1
NOP2
6 530 000
6 540 000
6 550 000
6 560 000
Genomic Position (bp)

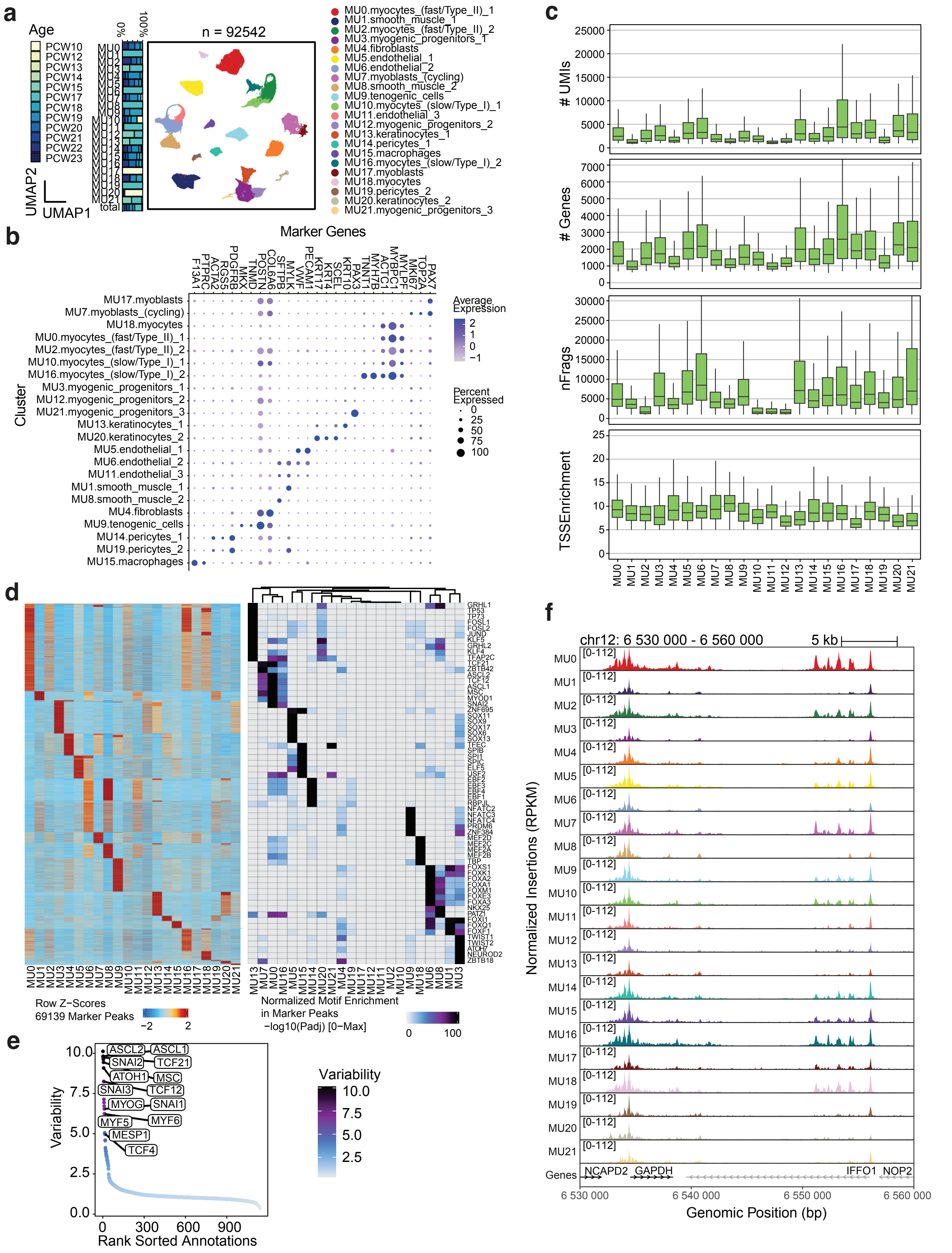
a
Age
PCW10
PCW12
PCW13
PCW14
PCW15
PCW17
PCW18
PCW19
PCW20
PCW21
PCW22
PCW23
0%
100%
n = 92542
UMAP2
UMAP1
MU0.myocytes_(fast/Type_II)_1
MU1.smooth_muscle_1
MU2.myocytes_(fast/Type_II)_2
MU3.myogenic_progenitors_1
MU4.fibroblasts
MU5.endothelial_1
MU6.endothelial_2
MU7.myoblasts_(cycling)
MU8.smooth_muscle_2
MU9.tenogenic_cells
MU10.myocytes_(slow/Type_I)_1
MU11.endothelial_3
MU12.myogenic_progenitors_2
MU13.keratinocytes_1
MU14.pericytes_1
MU15.macrophages
MU16.myocytes_(slow/Type_I)_2
MU17.myoblasts
MU18.myocytes
MU19.pericytes_2
MU20.keratinocytes_2
MU21.myogenic_progenitors_3
b
Marker Genes
Cluster
Average Expression
Percent Expressed
c
UMIs
Genes
nFrags
TSSEnrichment
d
Row Z-Scores
69139 Marker Peaks
Normalized Motif Enrichment in Marker Peaks
−log10(Padj) [0−Max]
e
Variability
Rank Sorted Annotations
ASCL2
ASCL1
SNAI2
TCF21
ATOH1
MSC
SNAI3
TCF12
MYOG
SNAI1
MYF5
MYF6
MESP1
TCF4
f
chr12: 6 530 000 - 6 560 000
5 kb
Normalized Insertions (RPKM)
Genes
NCAPD2
GAPDH
IFFO1
NOP2
Genomic Position (bp)

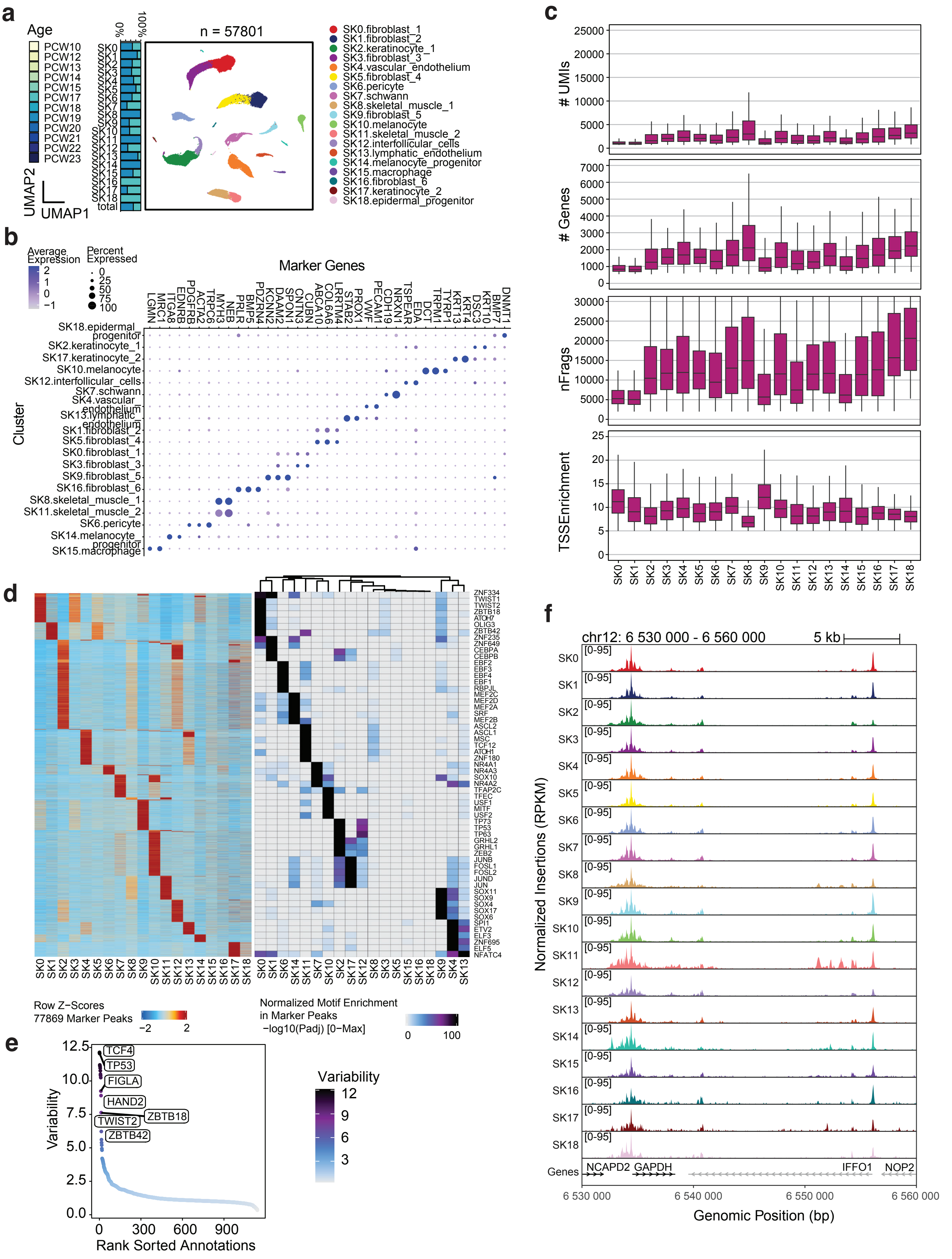

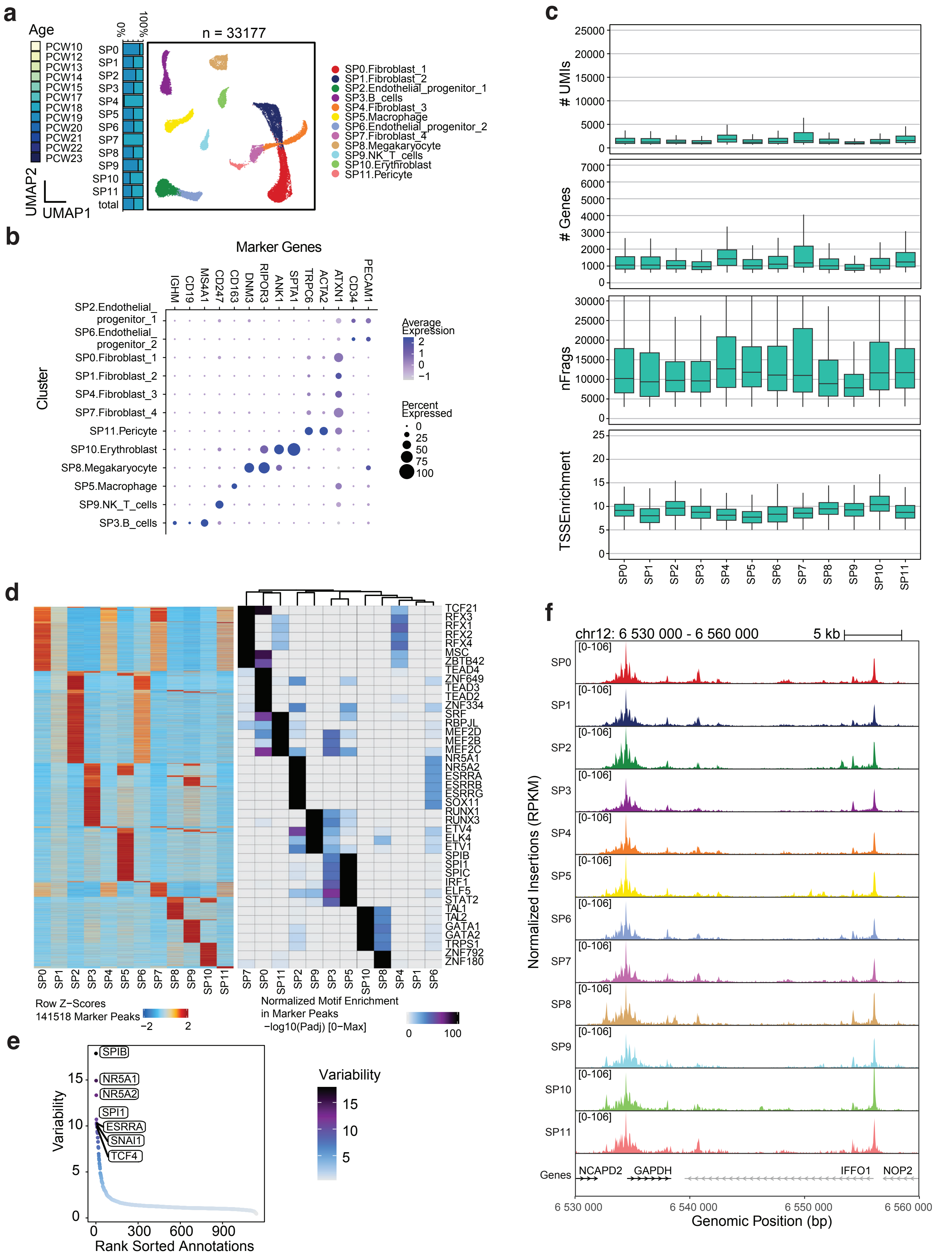
a
Age
PCW10
PCW12
PCW13
PCW14
PCW15
PCW17
PCW18
PCW19
PCW20
PCW21
PCW22
PCW23
0%
100%
SP0
SP1
SP2
SP3
SP4
SP5
SP6
SP7
SP8
SP9
SP10
SP11
total
n = 33177
UMAP2
UMAP1
SP0.Fibroblast_1
SP1.Fibroblast_2
SP2.Endothelial_progenitor_1
SP3.B_cells
SP4.Fibroblast_3
SP5.Macrophage
SP6.Endothelial_progenitor_2
SP7.Fibroblast_4
SP8.Megakaryocyte
SP9.NK_T_cells
SP10.Erythroblast
SP11.Pericyte
b
Marker Genes
IGHM
CD19
MS4A1
CD247
CD163
DNM3
RIPOR3
ANK1
SPTA1
TRPC6
ACTA2
ATXN1
CD34
PECAM1
Cluster
SP2.Endothelial_progenitor_1
SP6.Endothelial_progenitor_2
SP0.Fibroblast_1
SP1.Fibroblast_2
SP4.Fibroblast_3
SP7.Fibroblast_4
SP11.Pericyte
SP10.Erythroblast
SP8.Megakaryocyte
SP5.Macrophage
SP9.NK_T_cells
SP3.B_cells
Average Expression
2
1
0
−1
Percent Expressed
0
25
50
75
100
c
UMIs
Genes
nFrags
TSSEnrichment
SP0
SP1
SP2
SP3
SP4
SP5
SP6
SP7
SP8
SP9
SP10
SP11
d
TCF21
RFX3
RFX1
RFX2
RFX4
MSC
ZBTB42
TEAD4
ZNF649
TEAD3
TEAD2
ZNF334
SRF
RBPJL
MEF2D
MEF2B
MEF2C
NR5A1
NR5A2
ESRRA
ESRRB
ESRRG
SOX11
RUNX1
RUNX3
ETV4
ELK4
ETV1
SPIB
SPI1
SPIC
IRF1
ELF5
STAT2
TAL1
TAL2
GATA1
GATA2
TRPS1
ZNF792
ZNF180
Row Z−Scores
141518 Marker Peaks
−2
2
Normalized Motif Enrichment
in Marker Peaks
−log10(Padj) [0−Max]
0
100
e
Variability
SPIB
NR5A1
NR5A2
SPI1
ESRRA
SNAI1
TCF4
Rank Sorted Annotations
f
chr12: 6 530 000 - 6 560 000
5 kb
[0-106]
Normalized Insertions (RPKM)
Genes
NCAPD2
GAPDH
IFFO1
NOP2
6 530 000
6 540 000
6 550 000
6 560 000
Genomic Position (bp)

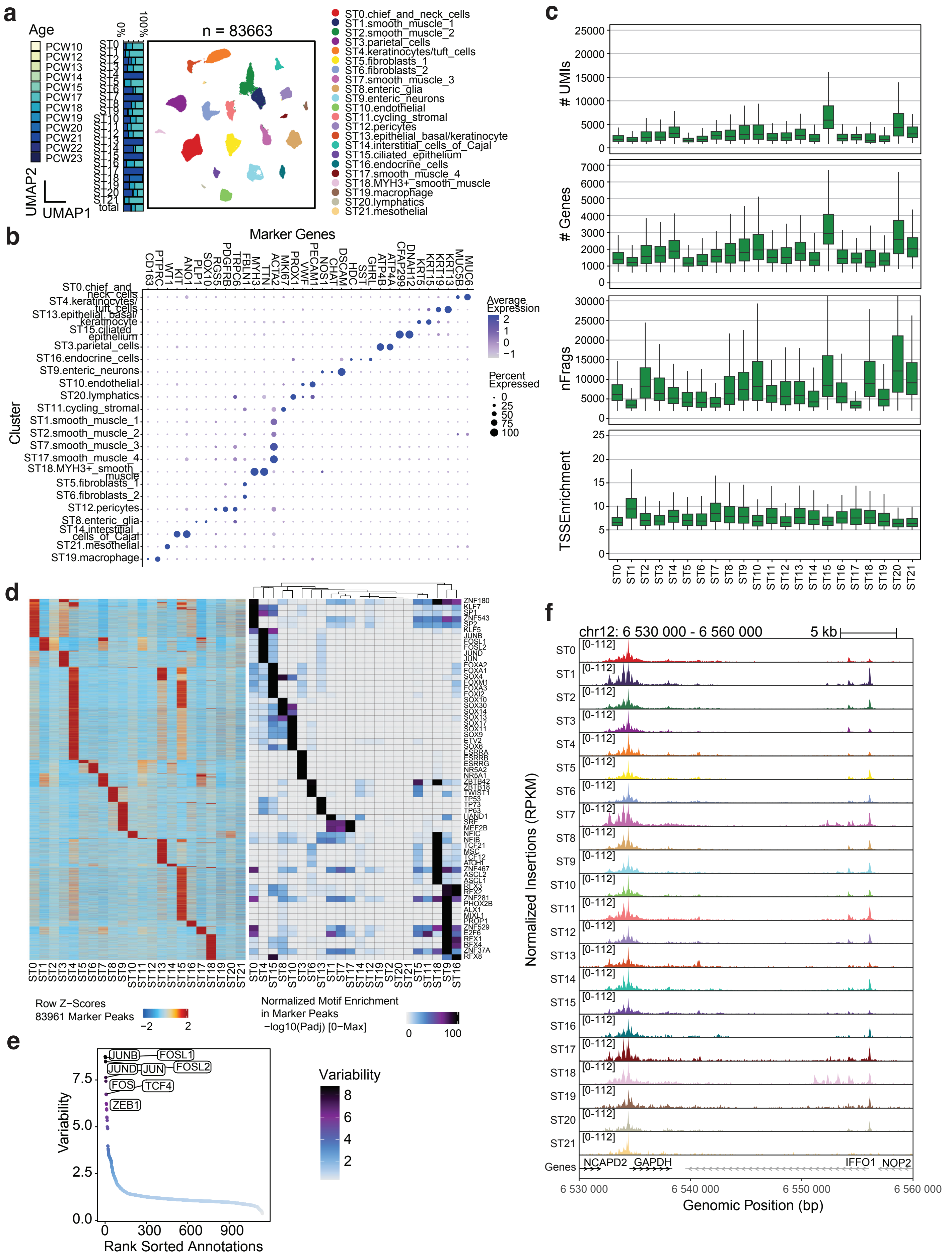
a
Age
PCW10
PCW12
PCW13
PCW14
PCW15
PCW17
PCW18
PCW19
PCW20
PCW21
PCW22
PCW23
0%
100%
n = 83663
UMAP2
UMAP1
ST0.chief_and_neck_cells
ST1.smooth_muscle_1
ST2.smooth_muscle_2
ST3.parietal_cells
ST4.keratinocytes/tuft_cells
ST5.fibroblasts_1
ST6.fibroblasts_2
ST7.smooth_muscle_3
ST8.enteric_glia
ST9.enteric_neurons
ST10.endothelial
ST11.cycling_stromal
ST12.pericytes
ST13.epithelial_basal/keratinocyte
ST14.interstitial_cells_of_Cajal
ST15.ciliated_epithelium
ST16.endocrine_cells
ST17.smooth_muscle_4
ST18.MYH3+_smooth_muscle
ST19.macrophage
ST20.lymphatics
ST21.mesothelial
b
Marker Genes
Cluster
Average Expression
Percent Expressed
c
UMIs
Genes
nFrags
TSSEnrichment
d
Row Z-Scores
83961 Marker Peaks
Normalized Motif Enrichment in Marker Peaks
−log10(Padj) [0–Max]
e
Variability
Rank Sorted Annotations
JUNB
FOSL1
JUND
JUN
FOSL2
FOS
TCF4
ZEB1
f
chr12: 6 530 000 - 6 560 000
5 kb
[0-112]
Normalized Insertions (RPKM)
Genes
NCAPD2
GAPDH
IFFO1
NOP2
Genomic Position (bp)
6 530 000
6 540 000
6 550 000
6 560 000

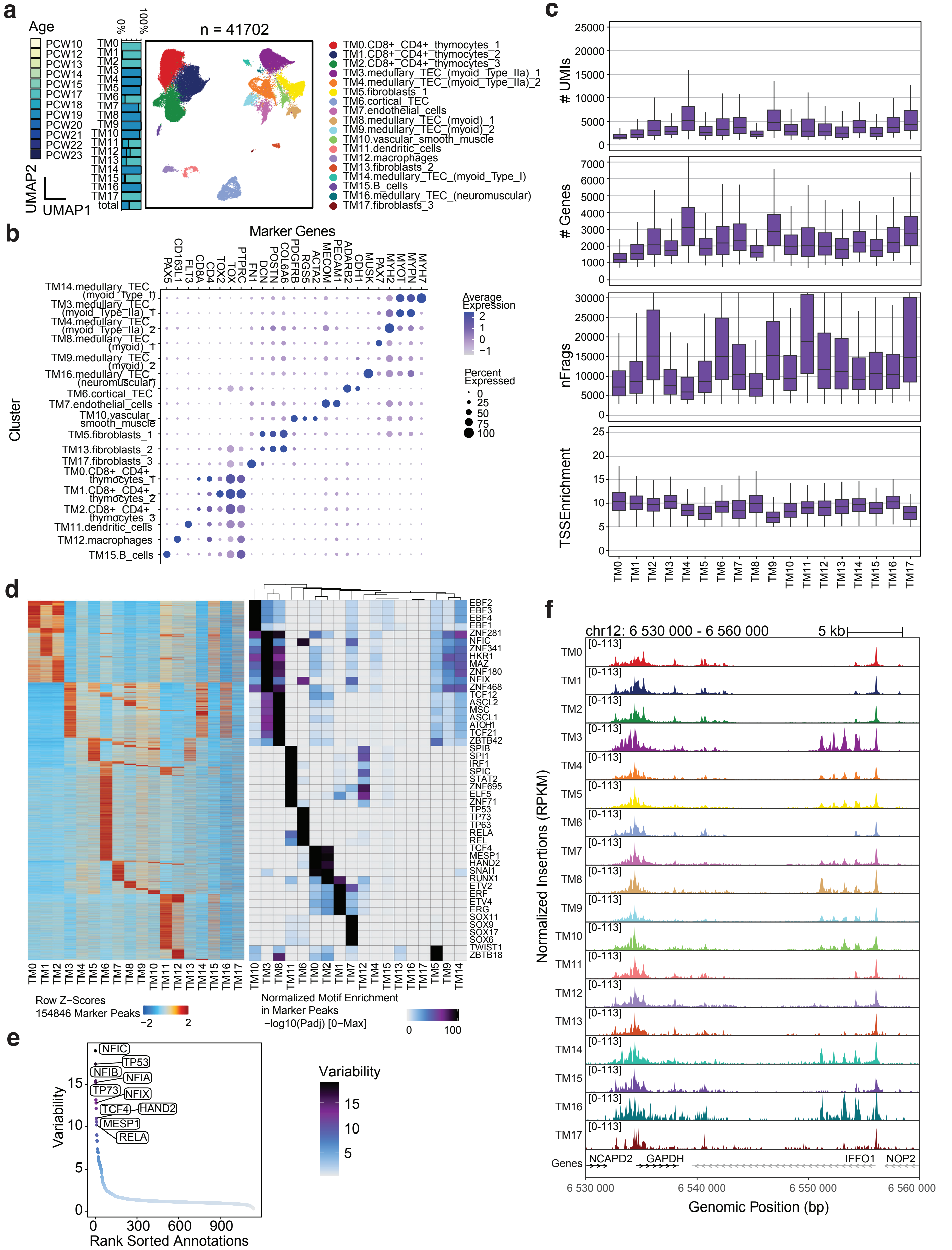
a
Age
PCW10
PCW12
PCW13
PCW14
PCW15
PCW17
PCW18
PCW19
PCW20
PCW21
PCW22
PCW23
0%
100%
TM0
TM1
TM2
TM3
TM4
TM5
TM6
TM7
TM8
TM9
TM10
TM11
TM12
TM13
TM14
TM15
TM16
TM17
total
UMAP2
UMAP1
n = 41702
TM0.CD8+_CD4+_thymocytes_1
TM1.CD8+_CD4+_thymocytes_2
TM2.CD8+_CD4+_thymocytes_3
TM3.medullary_TEC_(myoid_Type_IIa)_1
TM4.medullary_TEC_(myoid_Type_IIa)_2
TM5.fibroblasts_1
TM6.cortical_TEC
TM7.endothelial_cells
TM8.medullary_TEC_(myoid)_1
TM9.medullary_TEC_(myoid)_2
TM10.vascular_smooth_muscle
TM11.dendritic_cells
TM12.macrophages
TM13.fibroblasts_2
TM14.medullary_TEC_(myoid_Type_I)
TM15.B_cells
TM16.medullary_TEC_(neuromuscular)
TM17.fibroblasts_3
b
Marker Genes
PAX5
CD163L1
FLT3
CD8A
CD4
TOX2
TOX
PTPRC
FN1
DCN
POSTN
COL6A6
PDGFRB
RGS5
ACTA2
MECOM
PECAM1
ADARB2
CDH1
MUSK
PAX7
MYH2
MYOT
MYPN
MYH7
Cluster
Average Expression
2
1
0
−1
Percent Expressed
0
25
50
75
100
c
UMIs
Genes
nFrags
TSSEnrichment
d
Row Z-Scores
154846 Marker Peaks
−2
2
Normalized Motif Enrichment in Marker Peaks
−log10(Padj) [0−Max]
0
100
EBF2
EBF3
EBF4
EBF1
ZNF281
NFIC
ZNF341
HKR1
MAZ
ZNF180
NFIX
ZNF468
TCF12
ASCL2
MSC
ASCL1
ATOH1
TCF21
ZBTB42
SPIB
SPI1
IRF1
SPIC
STAT2
ZNF695
ELF5
ZNF71
TP53
TP73
TP63
RELA
REL
TCF4
MESP1
HAND2
SNAI1
RUNX1
ETV2
ERF
ETV4
ERG
SOX11
SOX9
SOX17
SOX6
TWIST1
ZBTB18
e
Variability
Rank Sorted Annotations
NFIC
TP53
NFIB
NFIA
TP73
NFIX
TCF4
HAND2
MESP1
RELA
f
chr12: 6 530 000 - 6 560 000
5 kb
[0-113]
Normalized Insertions (RPKM)
Genes
NCAPD2
GAPDH
IFFO1
NOP2
6 530 000
6 540 000
6 550 000
6 560 000
Genomic Position (bp)

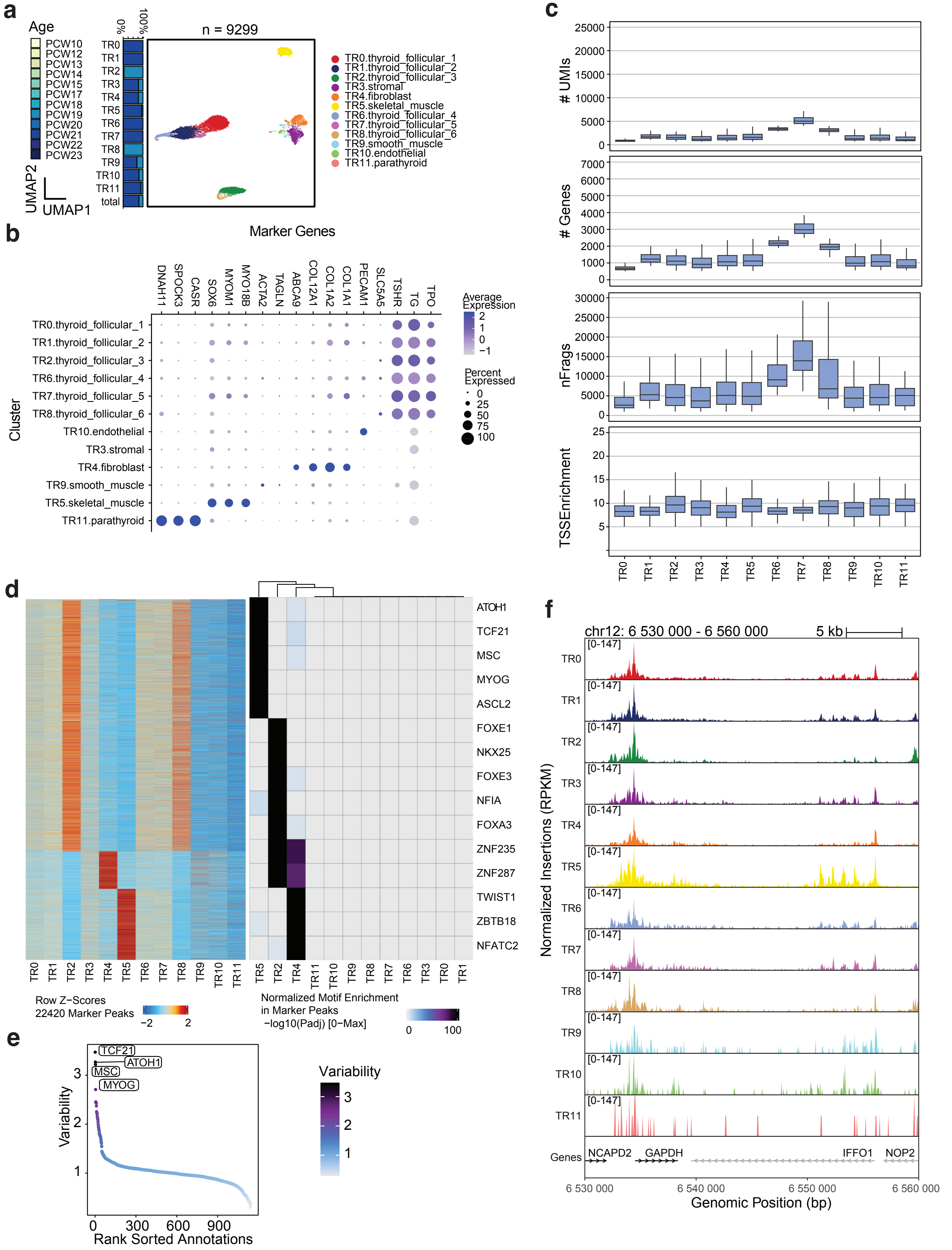

a
Age
PCW10
PCW12
PCW13
PCW14
PCW15
PCW17
PCW18
PCW19
PCW20
PCW21
PCW22
PCW23
0%
100%
TR0
TR1
TR2
TR3
TR4
TR5
TR6
TR7
TR8
TR9
TR10
TR11
total
n = 9299
UMAP2
UMAP1
TR0.thyroid_follicular_1
TR1.thyroid_follicular_2
TR2.thyroid_follicular_3
TR3.stromal
TR4.fibroblast
TR5.skeletal_muscle
TR6.thyroid_follicular_4
TR7.thyroid_follicular_5
TR8.thyroid_follicular_6
TR9.smooth_muscle
TR10.endothelial
TR11.parathyroid
b
Marker Genes
DNAH11
SPOCK3
CASR
SOX6
MYOM1
MYO18B
ACTA2
TAGLN
ABCA9
COL12A1
COL1A2
COL1A1
PECAM1
SLC5A5
TSHR
TG
TPO
Cluster
TR0.thyroid_follicular_1
TR1.thyroid_follicular_2
TR2.thyroid_follicular_3
TR6.thyroid_follicular_4
TR7.thyroid_follicular_5
TR8.thyroid_follicular_6
TR10.endothelial
TR3.stromal
TR4.fibroblast
TR9.smooth_muscle
TR5.skeletal_muscle
TR11.parathyroid
Average Expression
2
1
0
−1
Percent Expressed
0
25
50
75
100
c
UMIs
Genes
nFrags
TSSEnrichment
TR0
TR1
TR2
TR3
TR4
TR5
TR6
TR7
TR8
TR9
TR10
TR11
d
ATOH1
TCF21
MSC
MYOG
ASCL2
FOXE1
NKX25
FOXE3
NFIA
FOXA3
ZNF235
ZNF287
TWIST1
ZBTB18
NFATC2
Row Z−Scores
22420 Marker Peaks
−2
2
Normalized Motif Enrichment
in Marker Peaks
−log10(Padj) [0−Max]
0
100
e
TCF21
ATOH1
MSC
MYOG
Variability
Rank Sorted Annotations
0
300
600
900
Variability
3
2
1
f
chr12: 6 530 000 - 6 560 000
5 kb
[0-147]
Normalized Insertions (RPKM)
Genes
NCAPD2
GAPDH
IFFO1
NOP2
6 530 000
6 540 000
6 550 000
6 560 000
Genomic Position (bp)

Supplementary Note 3: *In silico* marginalization for motif synergy across cell types.

In the main analysis of motif synergy, we evaluated synergy for each composite motif in the cell type with the most predictive instances of that composite motif. To assess the specificity of our synergy predictions and evaluate performance on negative controls, we also performed the *in silico* marginalization experiments on all 138 candidate motif pairs across all 189 cell types (26,082 total tests).

The effect of a given motif or motif pair is defined as the difference in predicted accessibility (in log counts) for edited versus reference non-accessible sequences in the *in silico* analysis. Our analysis identified motif pairs where the joint effect was significantly greater than the sum of independent effects. Therefore, we first examined the distribution of differences between the joint effect and the sum of independent effects, for the background set (all 26,082 tests), compared to our foreground set (67 synergistic pairs we reported as synergistic) (**Note S3 Fig. a**). In the background, the median difference was 0.04 log counts. This confirms that even when testing motifs which are candidates for cooperativity because they form composite motifs, the models predicted a negligible synergistic effect for the majority of motif-cell type combinations.

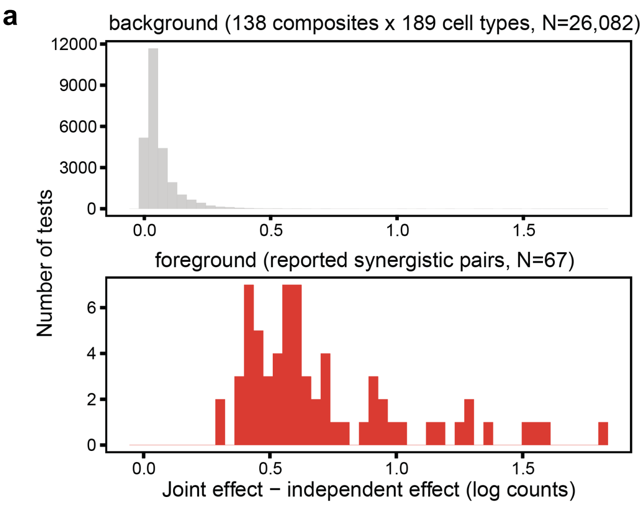

a. Distribution of difference in joint vs independent effects across the background set of 26,082 tests for synergy (138 motif pairs x 189 cell types), versus the reported synergistic pairs (with hard and/or soft synergy).

The biological patterns observed within these 2,317 high-confidence hits further validate our approach. A subset of motif pairs are ubiquitously predicted to be synergistic (e.g., pairs containing ETS, BZIP, SP/KLF and NFY motifs), while many others exhibit striking cell-type specificity that aligns with known TF expression. For example, strong synergy was predicted for motif pairs containing GATA, HNF4 and FOX motifs in liver; HD, MEIS and TBX motifs in eye and neural cells; RUNX and IKZF1 motifs in immune cells; FOX, GRHL and NKX motifs in lung and stomach cell types; and MEF2 and MEIS motifs cardiomyocytes and other muscle cell types. In contrast, these same motifs pairs showed limited predicted synergy in other lineages where the TFs are not expressed or and/or not co-expressed, providing hundreds of implicit negative controls.

This analysis has some limitations. First, while we manually verified that the inserted sequences were driving the predicted synergistic effects for every motif/cell type combination for the main analysis (Fig. 4) based on model interpretation using DeepLIFT on edited sequences, it was not feasible to confirm sequences driving synergistic predictions across all tests. Second, the pleiotropic roles of TFs and the similarity of their motifs make it challenging to create a comprehensive gold-standard set of true negatives. This prevents a systematic quantification of false positive rates across all cell types and motif pairs.

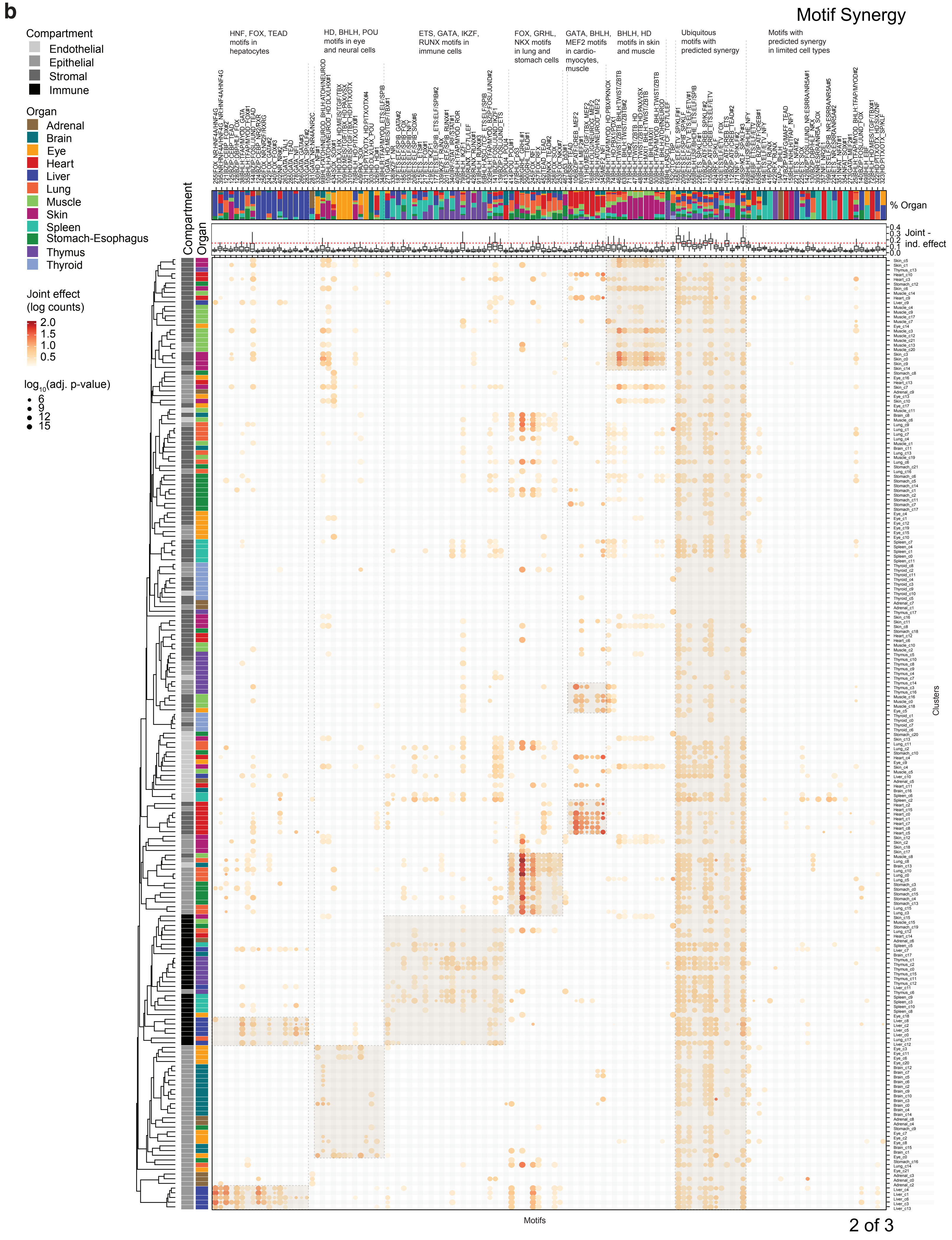
b
Motif Synergy
Compartment
Endothelial
Epithelial
Stromal
Immune
Organ
Adrenal
Brain
Eye
Heart
Liver
Lung
Muscle
Skin
Spleen
Stomach-Esophagus
Thymus
Thyroid
Joint effect
(log counts)
2.0
1.5
1.0
0.5
log10(adj. p-value)
6
9
12
15
HNF, FOX, TEAD motifs in hepatocytes
HD, BHLH, POU motifs in eye and neural cells
ETS, GATA, IKZF, RUNX motifs in immune cells
FOX, GRHL, NKX motifs in lung and stomach cells
GATA, BHLH, MEF2 motifs in cardio-myocytes, muscle
BHLH, HD motifs in skin and muscle
Ubiquitous motifs with predicted synergy
Motifs with predicted synergy in limited cell types
Compartment
Organ
% Organ
Joint - ind. effect
0.4
0.3
0.2
0.1
0.0
Clusters
Motifs
2 of 3

b. Dotplot showing the results of performing in silico marginalization experiments to test synergy between all 138 candidate motif pairs in all 189 cell types with ChromBPNet models. Columns represent motifs, and rows represent cell type clusters in order of the dendrogram from Fig. 1c. Dots are coloured by the joint effect (in log counts) of the two motifs at their optimal arrangement in that cell type, and size is proportional to the -log10 adjusted p-value for the effect. Row annotations indicate tissue compartment and organ. Barplots for columns depict the distribution of cell types in which each motif pair is predicted to be synergistic, broken down by organ. Boxplots for columns depict the distribution of difference between joint and independent motif effects (in log counts) across all 189 cell types, per motif. Dots are shown for the 2,317 (out of 138 x 189 = 26,082) experiments predicted to be synergistic based on adjusted p-value and difference in joint and independent effects (see Methods).

For each motif pair predicted to be synergistic with hard syntax, we report the results of the *in silico* marginalization experiments in the cell type with the most predictive instances for the corresponding composite motif. The results are summarized in Supplementary Table 7 and **Note S3 Fig. c**.

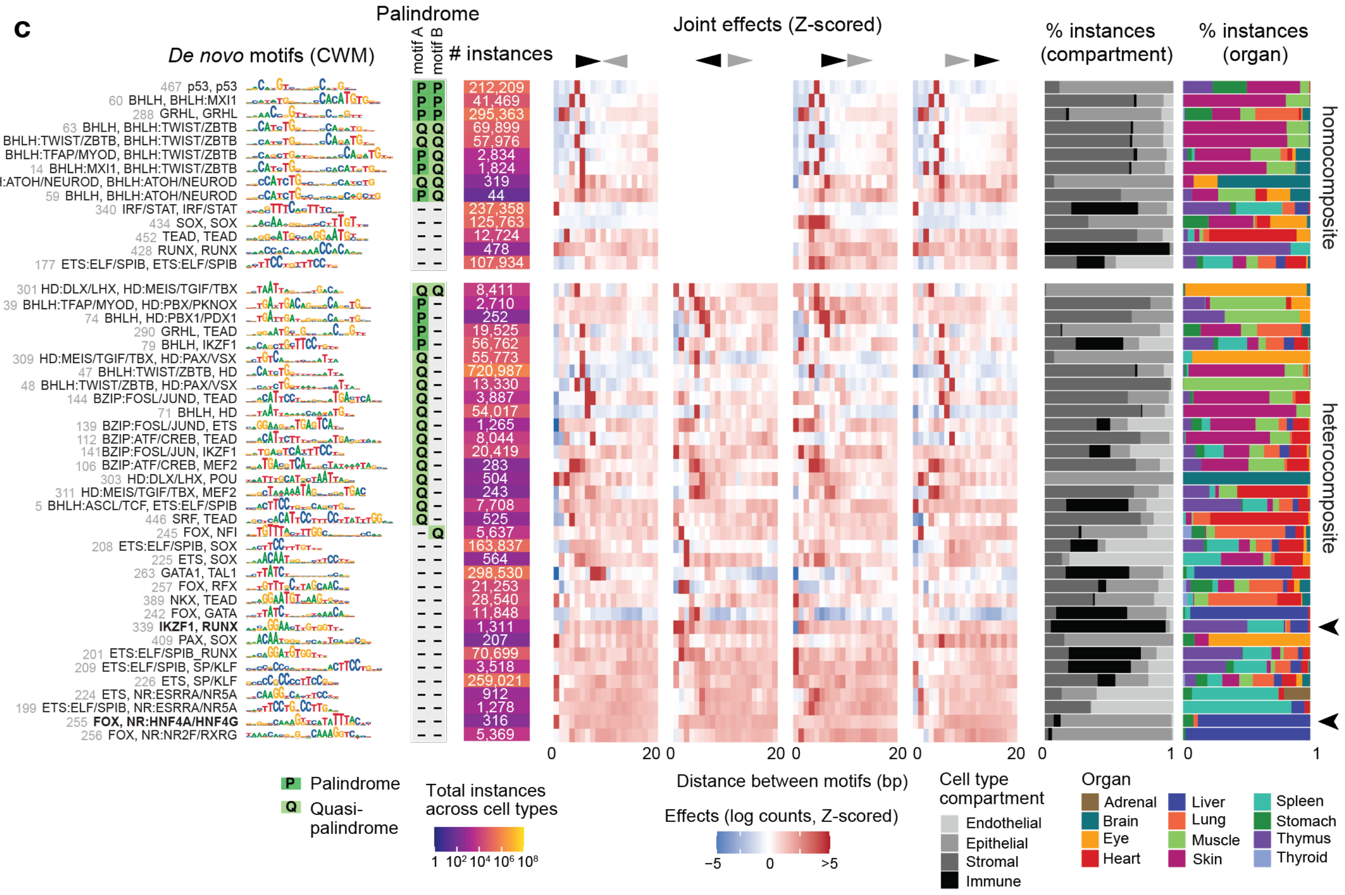

c. Summary of N=48 composite motifs inferred to be synergistic with hard syntax preferences. Left to right: de novo CWM-representation, annotation of palindromic sequences tested, total number of motif instances across cell types, predicted joint effects of inserted motifs at all arrangements at up to 20 bp (Z-scores were computed across all 200 bp), and distribution of motif instances across tissue compartments and organs. Arrowheads and boldface indicate motifs shown in Fig. 4l-m.
