# Supplementary material for: Multiomics and deep learning dissect regulatory syntax in human development
Source: Nature. 2026 Apr 8;653(8116):1240–53. doi: 10.1038/s41586-026-10326-9 (PMC13216069; doi:10.1038/s41586-026-10326-9)
Supplement: Supplementary file 2 — Reporting Summary [file 41586_2026_10326_MOESM2_ESM.pdf]

nature portfolio

Corresponding author(s): William J. Greenleaf, Kyle K. H. Farh, Anshul Kundaje

Last updated by author(s): 01/16/2026

Reporting Summary

Nature Portfolio wishes to improve the reproducibility of the work that we publish. This form provides structure for consistency and transparency in reporting. For further information on Nature Portfolio policies, see our Editorial Policies and the Editorial Policy Checklist.

Statistics

For all statistical analyses, confirm that the following items are present in the figure legend, table legend, main text, or Methods section.

n/a	Confirmed	
☐	☒	The exact sample size (*n*) for each experimental group/condition, given as a discrete number and unit of measurement
☐	☒	A statement on whether measurements were taken from distinct samples or whether the same sample was measured repeatedly
☐	☒	The statistical test(s) used AND whether they are one- or two-sided<br>*Only common tests should be described solely by name; describe more complex techniques in the Methods section.*
☐	☒	A description of all covariates tested
☐	☒	A description of any assumptions or corrections, such as tests of normality and adjustment for multiple comparisons
☐	☒	A full description of the statistical parameters including central tendency (e.g. means) or other basic estimates (e.g. regression coefficient) AND variation (e.g. standard deviation) or associated estimates of uncertainty (e.g. confidence intervals)
☐	☒	For null hypothesis testing, the test statistic (e.g. *F*, *t*, *r*) with confidence intervals, effect sizes, degrees of freedom and *P* value noted<br>*Give P values as exact values whenever suitable.*
☒	☐	For Bayesian analysis, information on the choice of priors and Markov chain Monte Carlo settings
☒	☐	For hierarchical and complex designs, identification of the appropriate level for tests and full reporting of outcomes
☐	☒	Estimates of effect sizes (e.g. Cohen's *d*, Pearson's *r*), indicating how they were calculated

Our web collection on statistics for biologists contains articles on many of the points above.

Software and code

Policy information about availability of computer code

Data collection	No software was used in the data collection.
Data analysis	All analysis co de is available at https://github.com/GreenleafLab/HDMA and archived on Zenodo at https://doi.org/10.5281/zenodo.17298234. We used the following open source software:<br>Snakemake (v7.15.1)<br>bcl2fastq (v2.20.0.422)<br>fastp (v0.23.2)<br>bowtie2 (v2.5.0)<br>STAR (v2.5.4b)<br>featureCounts (v2.0.1)<br>UMI-tools (v1.1.2)<br>Seurat (v4.3.0)<br>R (v4.1.2)<br>celda (v1.6.1)<br>ArchR (v1.0.2)<br>Macs2 (v2.2.2.7.1, v2.2.9.1)<br>ABC (adapted at https://github.com/GreenleafLab/ABC-Enhancer-Gene-Prediction-CustomRegions, commit b3d2156)<br>ChromBPNet (commit a5c231)<br>deeplift (0.6.13.0)<br>tfmodisco-lite (v2.0.7)<br>gimmemotifs (v0.18.0)

BAMboozle (v0.5.0)
TOMTOM (v4.111.2)
Fi-NeMo (v0.23, commit b81876d)
NucleoATAC (adapted at https://github.com/sjessa/NucleoATAC, v0.4.1)
tangermeme (v0.4.3)
g-chromVAR (v0.3.2)
rtracklayer (v1.54.0)
BPCells (v0.2.0)

For manuscripts utilizing custom algorithms or software that are central to the research but not yet described in published literature, software must be made available to editors and reviewers. We strongly encourage code deposition in a community repository (e.g. GitHub). See the Nature Portfolio guidelines for submitting code & software for further information.

Data

Policy information about availability of data

All manuscripts must include a data availability statement. This statement should provide the following information, where applicable:

- Accession codes, unique identifiers, or web links for publicly available datasets
- A description of any restrictions on data availability
- For clinical datasets or third party data, please ensure that the statement adheres to our policy

All processed data (including fragment files, counts matrices, cell annotations, global caCRE annotations, ChromBPNet models, motif lexicon, motif instances, and genomic tracks) are deposited at https://zenodo.org/communities/hdma. Raw, anonymized sequencing data have been deposited to SRA (PRJNA1402391). Metadata for raw genomic data produced in our study has been deposited at https://doi.org/10.5281/zenodo.17259745. A description of all data types and the corresponding URLs is provided in Table S14. ENCODE v4 cCREs were downloaded from the publicly available database: https://downloads.wenglab.org/Registry-V4/GRCh38-cCREs.bed.

Research involving human participants, their data, or biological material

Policy information about studies with human participants or human data. See also policy information about sex, gender (identity/presentation), and sexual orientation and race, ethnicity and racism.

Reporting on sex and gender	For human tissue samples, sex was defined based on the expression levels of genes on the Y chromosome, and is reported in sample meta data and figures where appropriate.
Reporting on race, ethnicity, or other socially relevant groupings	No information on race, ethnicity, or other social groupings was obtained.
Population characteristics	Age (post-conception weeks) and sex are reported for all samples. Age for all samples spanned 10-23 post-conception weeks, and samples were obtained from male and female embryos. No additional population characteristics were obtained.
Recruitment	De-identified tissue samples were collected at Stanford University School of Medicine from elective termination of pregnancy procedures with informed consent for the research use of tissues in observance of relevant legal and institutional ethical regulations.
Ethics oversight	De-identified tissue samples were collected at Stanford University School of Medicine from elective termination of pregnancy procedures with informed consent for the research use of tissues in observance of relevant legal and institutional ethical regulations. No demographic information was collected. Consent was obtained by the medical team. The relevant tissue sample processing and analyses were performed under protocol SCRO-796, approved by the Stem Cell Research Oversight Panel (SCRO) at Stanford.

Note that full information on the approval of the study protocol must also be provided in the manuscript.

Field-specific reporting

Please select the one below that is the best fit for your research. If you are not sure, read the appropriate sections before making your selection.

☒ Life sciences ☐ Behavioural & social sciences ☐ Ecological, evolutionary & environmental sciences

For a reference copy of the document with all sections, see nature.com/documents/nr-reporting-summary-flat.pdf

Life sciences study design

All studies must disclose on these points even when the disclosure is negative.

Sample size	No statistical measures were used to determine sample size. For human samples, sample size was determined by the availability of tissue. For each tissue, we verified presence of the main canonical tissue-specific cell types. No claims were made relying on quantities of samples from each tissue.

Data exclusions	All of the data acquired was utilized for analysis, and filtering of single cells and clusters not retained for downstream analysis is specified in the Methods.
Replication	For each of the 12 organs, multiple samples per organ were collected and studied in aggregate. Direct replication was not possible since age, sex, and tissue type was dependent on tissue availability. No claims were made regarding quantities or proportions of cell types.
Randomization	Samples were allocated to groups according to organs from which they were derived. No randomization was required because sequencing and data analysis methods are not affected by sample randomization.
Blinding	Blinding was not applicable to this study as no effect of treatment or perturbations were assessed.

Reporting for specific materials, systems and methods

We require information from authors about some types of materials, experimental systems and methods used in many studies. Here, indicate whether each material, system or method listed is relevant to your study. If you are not sure if a list item applies to your research, read the appropriate section before selecting a response.

Materials & experimental systems

n/a	Involved in the study
☒	☐ Antibodies
☒	☐ Eukaryotic cell lines
☒	☐ Palaeontology and archaeology
☒	☐ Animals and other organisms
☒	☐ Clinical data
☒	☐ Dual use research of concern
☒	☐ Plants

Methods

n/a	Involved in the study
☒	☐ ChIP-seq
☒	☐ Flow cytometry
☒	☐ MRI-based neuroimaging

Plants

Seed stocks	*Report on the source of all seed stocks or other plant material used. If applicable, state the seed stock centre and catalogue number. If plant specimens were collected from the field, describe the collection location, date and sampling procedures.*
Novel plant genotypes	*Describe the methods by which all novel plant genotypes were produced. This includes those generated by transgenic approaches, gene editing, chemical/radiation-based mutagenesis and hybridization. For transgenic lines, describe the transformation method, the number of independent lines analyzed and the generation upon which experiments were performed. For gene-edited lines, describe the editor used, the endogenous sequence targeted for editing, the targeting guide RNA sequence (if applicable) and how the editor was applied.*
Authentication	*Describe any authentication procedures for each seed stock used or novel genotype generated. Describe any experiments used to assess the effect of a mutation and, where applicable, how potential secondary effects (e.g. second site T-DNA insertions, mosiacism, off-target gene editing) were examined.*
